# Interfacing with the Brain: How Nanotechnology Can Contribute

**DOI:** 10.1021/acsnano.4c10525

**Published:** 2025-03-10

**Authors:** Abdullah A. A. Ahmed, Nuria Alegret, Bethany Almeida, Ramón Alvarez-Puebla, Anne M. Andrews, Laura Ballerini, Juan J. Barrios-Capuchino, Charline Becker, Robert H. Blick, Shahin Bonakdar, Indranath Chakraborty, Xiaodong Chen, Jinwoo Cheon, Gerwin Chilla, Andre Luiz Coelho Conceicao, James Delehanty, Martin Dulle, Alexander L. Efros, Matthias Epple, Mark Fedyk, Neus Feliu, Miao Feng, Rafael Fernández-Chacón, Irene Fernandez-Cuesta, Niels Fertig, Stephan Förster, Jose A. Garrido, Michael George, Andreas H. Guse, Norbert Hampp, Jann Harberts, Jili Han, Hauke R. Heekeren, Ulrich G. Hofmann, Malte Holzapfel, Hessam Hosseinkazemi, Yalan Huang, Patrick Huber, Taeghwan Hyeon, Sven Ingebrandt, Marcello Ienca, Armin Iske, Yanan Kang, Gregor Kasieczka, Dae-Hyeong Kim, Kostas Kostarelos, Jae-Hyun Lee, Kai-Wei Lin, Sijin Liu, Xin Liu, Yang Liu, Christian Lohr, Volker Mailänder, Laura Maffongelli, Saad Megahed, Alf Mews, Marina Mutas, Leroy Nack, Nako Nakatsuka, Thomas G. Oertner, Andreas Offenhäusser, Martin Oheim, Ben Otange, Ferdinand Otto, Enrico Patrono, Bo Peng, Alessandra Picchiotti, Filippo Pierini, Monika Pötter-Nerger, Maria Pozzi, Arnd Pralle, Maurizio Prato, Bing Qi, Pedro Ramos-Cabrer, Ute Resch Genger, Norbert Ritter, Marten Rittner, Sathi Roy, Francesca Santoro, Nicolas W. Schuck, Florian Schulz, Erkin Şeker, Marvin Skiba, Martin Sosniok, Holger Stephan, Ruixia Wang, Ting Wang, K. David Wegner, Paul S. Weiss, Ming Xu, Chenxi Yang, Seyed Shahrooz Zargarian, Yuan Zeng, Yaofeng Zhou, Dingcheng Zhu, Robert Zierold, Wolfgang J. Parak

**Affiliations:** 1 Fachbereich Physik, Universität Hamburg, 22761 Hamburg, Germany; 2 Department of Physics, Faculty of Applied Science, Thamar University, Dhamar 87246, Yemen; 3 Biogipuzkoa HRI, Paseo Dr. Begiristain s/n, 20014 Donostia-San Sebastián, Spain; 4 Department of Chemical and Biomolecular Engineering, Clarkson University, Potsdam, New York 13699, United States; 5 Universitat Rovira i Virgili, 43007 Tarragona, Spain; 6 ICREA, 08010 Barcelona, Spain; 8 Department of Chemistry and Biochemistry, University of California, Los Angeles, Los Angeles, California 90095, United States; 9 Neuroscience Interdepartmental Program, University of California, Los Angeles, Los Angeles, California 90095, United States; 10 Department of Psychiatry and Biobehavioral Sciences, Semel Institute for Neuroscience & Human Behavior, and Hatos Center for Neuropharmacology, University of California, Los Angeles, Los Angeles, California 90095, United States; 11 California Nanosystems Institute, University of California, Los Angeles, Los Angeles, California 90095, United States; 12 Neuroscience Area, International School for Advanced Studies (SISSA/ISAS), Trieste 34136, Italy; 13 National Cell Bank Department, Pasteur Institute of Iran, P.O. Box 1316943551, Tehran, Iran; 14 School of Nano Science and Technology, Indian Institute of Technology Kharagpur, Kharagpur 721302, India; 15 Innovative Center for Flexible Devices (iFLEX), Max Planck − NTU Joint Lab for Artificial Senses, School of Materials Science and Engineering, Nanyang Technological University, Singapore 639798, Singapore; 16 Institute for Basic Science Center for Nanomedicine, Seodaemun-gu, Seoul 03722, Korea; 17 Advanced Science Institute, Yonsei University, Seodaemun-gu, Seoul 03722, Korea; 18 Department of Chemistry, Yonsei University, Seodaemun-gu, Seoul 03722, Korea; 19 Deutsches Elektronen-Synchrotron DESY, 22607 Hamburg, Germany; 20 U.S. Naval Research Laboratory, Washington, D.C. 20375, United States; 21 JCNS-1, Forschungszentrum Jülich, 52428 Jülich, Germany; 22 Inorganic Chemistry and Center for Nanointegration Duisburg-Essen (CeNIDE), University of Duisburg-Essen, 45117 Essen, Germany; 23 Center for Neuroengineering and Medicine, UC Davis, Sacramento, California 95817, United States; 24 Zentrum für Angewandte Nanotechnologie CAN, Fraunhofer-Institut für Angewandte Polymerforschung IAP, 20146 Hamburg, Germany; 25 Instituto de Biomedicina de Sevilla (IBiS), Hospital Universitario Virgen del Rocío/Consejo Superior de Investigaciones Científicas/Universidad de Sevilla, 41013 Seville, Spain; 26 Departamento de Fisiología Médica y Biofísica, Facultad de Medicina, Universidad de Sevilla, CIBERNED, ISCIII, 41013 Seville, Spain; 27 Nanion Technologies GmbH, 80339 München, Germany; 28 Catalan Institute of Nanoscience and Nanotechnology (ICN2), CSIC and BIST, 08193 Bellaterra, Spain; 29 The Calcium Signaling Group, Department of Biochemistry and Molecular Cell Biology, University Medical Center Hamburg-Eppendorf, 20251 Hamburg, Germany; 30 Fachbereich Chemie, Universität Marburg, 35032 Marburg, Germany; 31 Drug Delivery, Disposition and Dynamics, Monash Institute of Pharmaceutical Sciences, Monash University, Parkville, Victoria 3052, Australia; 32 Melbourne Centre for Nanofabrication, Victorian Node of the Australian National Fabrication Facility, Clayton, Victoria 3168, Australia; 33 Executive University Board, Universität Hamburg, 20148 Hamburg Germany; 34 Section for Neuroelectronic Systems, Department for Neurosurgery, University Medical Center Freiburg, 79108 Freiburg, Germany; 35 Faculty of Medicine, University of Freiburg, 79110 Freiburg, Germany; 36 Institute for Materials and X-ray Physics, Hamburg University of Technology, 21073 Hamburg, Germany; 37 Center for X-ray and Nano Science CXNS, Deutsches Elektronen-Synchrotron DESY, 22607 Hamburg, Germany; 38 Center for Nanoparticle Research, Institute for Basic Science (IBS), Seoul 08826, Republic of Korea; 39 School of Chemical and Biological Engineering, and Institute of Chemical Processes, Seoul National University, Seoul 08826, Republic of Korea; 40 Institute of Materials in Electrical Engineering 1, RWTH Aachen University, 52074 Aachen, Germany; 41 Institute for Ethics and History of Medicine, School of Medicine and Health, Technische Universität München (TUM), 81675 München, Germany; 42 Fachbereich Mathematik, Universität Hamburg, 20146 Hamburg, Germany; 43 Centre for Nanotechnology in Medicine, Faculty of Biology, Medicine & Health and The National Graphene Institute, University of Manchester, Manchester M13 9PL, United Kingdom; 44 State Key Laboratory of Environmental Chemistry and Ecotoxicology, Research Center for Eco-Environmental Sciences, Chinese Academy of Sciences, Beijing 100085, China; 45 University of the Chinese Academy of Sciences, Beijing 100049, China; 46 Fachbereich Biologie, Universität Hamburg, 20146 Hamburg, Germany; 47 Department of Dermatology, Center for Translational Nanomedicine, Universitätsmedizin der Johannes-Gutenberg, Universität Mainz, 55131 Mainz, Germany; 48 Institute of Medical Psychology, University of Lübeck, 23562 Lübeck, Germany; 49 Physics Department, Faculty of Science, Al-Azhar University, 4434104 Cairo, Egypt; 50 Fachbereich Chemie, Universität Hamburg, 20146 Hamburg, Germany; 51 Laboratory of Chemical Nanotechnology (CHEMINA), Neuro-X Institute, École Polytechnique Fédérale de Lausanne (EPFL), Geneva CH-1202, Switzerland; 52 Institute for Synaptic Neuroscience, University Medical Center Hamburg-Eppendorf, 20251 Hamburg, Germany; 53 Institute of Biological Information Processing - Bioelectronics, Forschungszentrum Jülich, 52425 Jülich, Germany; 54 Université Paris Cité, CNRS, Saints Pères Paris Institute for the Neurosciences, 75006 Paris, France; 55 Institute of Physiology, Czech Academy of Sciences, Prague 12000, Czech Republic; 56 Department of Biosystems and Soft Matter, Institute of Fundamental Technological Research, Polish Academy of Sciences, 02-106 Warsaw, Poland; 57 Head and Neurocenter, Department of Neurology, University Medical Center Hamburg-Eppendorf, 20246 Hamburg, Germany; 58 University at Buffalo, Department of Physics, Buffalo, New York 14260, United States; 59 CIC biomaGUNE, Basque Research and Technology Alliance (BRTA), 20014 Donostia-San Sebastián, Spain; 60 Department of Chemical and Pharmaceutical Sciences, University of Trieste, 34127 Trieste, Italy; 61 Basque Foundation for Science, Ikerbasque, 48013 Bilbao, Spain; 62 Division Biophotonics, Federal Institute for Materials Research and Testing (BAM), 12489 Berlin, Germany; 63 Executive Faculty Board, Faculty for Mathematics, Informatics and Natural Sciences, Universität Hamburg, 20345 Hamburg, Germany; 64 Department of Mechanical Engineering, Indian Institute of Technology Kharagpur, Kharagpur 721302, India; 65 Faculty of Electrical Engineering and Information Technology, RWTH Aachen, 52074 Aachen, Germany; 66 Institute of Psychology, Universität Hamburg, 20146 Hamburg, Germany; 67 Max Planck Research Group NeuroCode, Max Planck Institute for Human Development, 14195 Berlin, Germany; 68 Max Planck UCL Centre for Computational Psychiatry and Ageing Research, 14195 Berlin, Germany; 69 University of California, Davis, Davis, California 95616, United States; 70 Helmholtz-Zentrum Dresden-Rossendorf, Institute of Radiopharmaceutical Cancer Research, 01328 Dresden, Germany; 71 State Key Laboratory of Organic Electronics and Information Displays & Jiangsu Key Laboratory for Biosensors, Institute of Advanced Materials (IAM), Jiangsu National Synergetic Innovation Center for Advanced Materials (SICAM), Nanjing University of Posts and Telecommunications, Nanjing 210023, China; 72 Department of Bioengineering, University of California, Los Angeles, Los Angeles, California 90095, United States; 73 Department of Materials Science and Engineering, University of California, Los Angeles, Los Angeles, California 90095, United States; 74 College of Material, Chemistry and Chemical Engineering, Key Laboratory of Organosilicon Chemistry and Material Technology, Ministry of Education, Key Laboratory of Organosilicon Material Technology, Hangzhou Normal University, Hangzhou 311121, China; 75 Basque Foundation for Science, Ikerbasque, 48013 Bilbao, Spain; 76 Max Planck Institute for Polymer Research, Ackermannweg 10, 55129 Mainz, Germany; 77 School of Life Sciences, Southern University of Science and Technology, Shenzhen, 518055, China

**Keywords:** Nanoneuro interface, brain-on-a-chip, brain−machine interfaces, neuronal communication, nanostructured interface, extracellular recordings, electrode arrays, control of ion channels, neuro-implants, deep brain stimulation

## Abstract

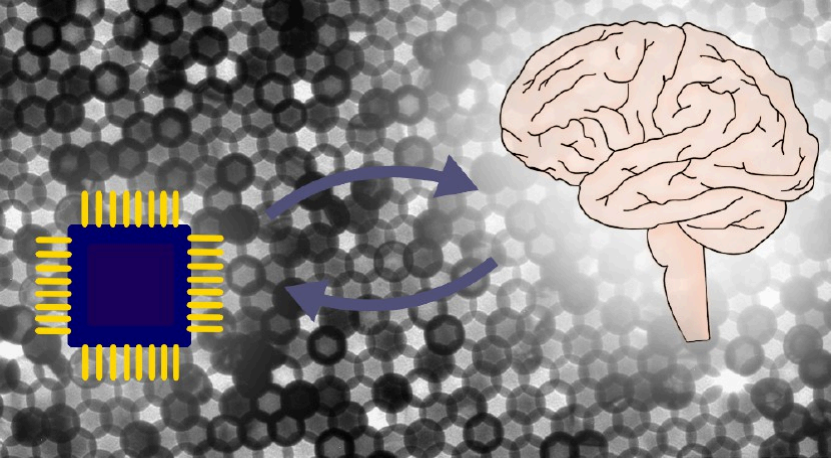

Interfacing artificial devices with the human brain is the central goal of neurotechnology. Yet, our imaginations are often limited by currently available paradigms and technologies. Suggestions for brain–machine interfaces have changed over time, along with the available technology. Mechanical levers and cable winches were used to move parts of the brain during the mechanical age. Sophisticated electronic wiring and remote control have arisen during the electronic age, ultimately leading to plug-and-play computer interfaces. Nonetheless, our brains are so complex that these visions, until recently, largely remained unreachable dreams. The general problem, thus far, is that most of our technology is mechanically and/or electrically engineered, whereas the brain is a living, dynamic entity. As a result, these worlds are difficult to interface with one another. Nanotechnology, which encompasses engineered solid-state objects and integrated circuits, excels at small length scales of single to a few hundred nanometers and, thus, matches the sizes of biomolecules, biomolecular assemblies, and parts of cells. Consequently, we envision nanomaterials and nanotools as opportunities to interface with the brain in alternative ways. Here, we review the existing literature on the use of nanotechnology in brain–machine interfaces and look forward in discussing perspectives and limitations based on the authors’ expertise across a range of complementary disciplines—from neuroscience, engineering, physics, and chemistry to biology and medicine, computer science and mathematics, and social science and jurisprudence. We focus on nanotechnology but also include information from related fields when useful and complementary.

One of the greatest questions of our time is how the human brain encodes information.^1−3^ It has been a dream for centuries to connect the human brain with external devices. A classic example is the “Nuremberg Funnel” (Nürnberger Trichter),^4^ which was proposed as a method for storing data and knowledge in our brain, without the effort of having to study. There are also dystopian scenarios, for example along the lines of thought control outlined in George Orwell’s novel 1984,^5^ in which surveillance is made by classic “telescreens”, which can transmit and receive, a potential future interface allowing one to “read out” and to “write into” the brain directly so that it could be abused by “Big Brother”. On the other hand, brain interfaces could have extremely positive impacts. Current developments target medical aid, for example, neuroprostheses for paralyzed people to control disabled limbs or even to re-enable walking.^6−10^

While strictly speaking, with current technologies, we are far from fully interfacing the brain, there are already quite a few medical devices connecting to neurons, and more generally, connecting to electrically excitable and electrogenic cells. Just as technologies that have become standard, such as pacemakers, that provide electrical stimuli to trigger contractions of the heart,^11^ electrodes implanted deep in the brain in the basal ganglia reduce movement disorder symptoms (such as Parkinson’s tremors),^12^ cochlear implants restore hearing,^13^ etc. In the quest for stable routine use, there are retinal implants to recover eyesight,^14−16^ and electrode implants to aid the movement of paraplegic patients.^17^ Most of these examples of neuromodulation are based on electric stimulation and/or detection, *i.e.*, they are multielectrode devices that stimulate voltage-gated ion channels, and electrically read out changes in membrane potential or currents through ion channels. At present, closed-loop deep-brain stimulation (DBS) with simultaneous sensing and stimulation is not available in Europe, but has been tested in the US and Japan. Conventionally, DBS is only used for stimulation. However, there are also devices for simultaneous sensing and stimulation toward closed-loop DBS.^18^

Work on basic devices for sensing and stimulation started decades ago.^19−27^ There remain several general technical challenges standing in the way of further applications. These technological needs involve achieving high integration density of many electrodes in small devices, biocompatibility to let neurons (or more generally electrically excitable and electrogenic cells) grow tightly on the active electrode sites (note that it is not trivial to quantify the neuron-electrode distance^28^), achieving long-term stability, and implementing feedback-loop control and bidirectional communication between the cells and the device. Moreover, from the biological side, even the optimal design for interfacing with the brain is not known. For example, for DBS, the mechanisms of action are not well understood; the modulation of voltage-gated ion channels seems to play a minor role.^29,30^ It appears that DBS acts not only by a single mechanism, but through several different mechanisms simultaneously, at the neuronal, populational, and systems physiological levels.

In the first section of this article, we outline how nanotechnology can help to improve cell-electrode interfaces, for example by making appropriate surface coatings of electrodes and by using materials that match the mechanical properties of the brain better than rigid, solid inorganic electrodes.

There are not only voltage-gated ion channels, but also a plethora of other ion channels, such as ligand-gated, mechanically gated (*e.g.*, in our ears), or light-gated (*e.g.*, in our eyes) ion channels. Certain electrically excitable cells can thus be excited by other than electrical stimuli. In turn, the electric signals of neurons (*i.e.*, action potentials and ionic currents) can be detected by nonelectronic readouts. Classic examples are voltage-sensitive fluorophores, which translate an electrical signal (membrane potential) into an optical readout.^31,32^ Nanotechnology offers enormous potential in this direction.^33^ Colloidal nanoparticles (NPs) can be used as local transducers, enabling translation between different excitation and readout methods. The use of NPs as signal transducers optimized for different types of ion channels will be discussed below.

There are many ethical restrictions concerning the testing and implementation of technologies for interfacing electrically excitable cells *in vivo*. While animal models are common practice, they are increasingly restricted in numbers for ethical reasons. In addition, taking the brain of an animal as an entire system allows for complexity, but may hamper direct observations of details, such as the direct interfaces between a cell and an electrode at the molecular level. On the other hand, experiments with single cultured cells or cellular networks are well suited for studying the interfacing of cells at the molecular levels.^34,35^ The drawback is that in such two-dimensional (2D) cultured systems, neurons are far from their native environments. Neurophysiology has introduced a number of intermediate options, such as cultures of slices of tissue, even from humans^36^ or more recently organoids.^37−40^ In a later section of this article, we highlight potential contributions of nanotechnology (and related fields) for making more realistic model systems. This direction will involve three-dimensional (3D) patterning (*i.e.*, printing) of supports and scaffolds for the 2.5–3D growth of neuronal cultures together with advanced microfluidics, or direct 3D printing of neurons, ultimately targeting a “brain-on-a-chip” (BoC).

Current technologies enable interactions with the human brain, but remain highly limited. Standard clinical platforms include multielectrode arrays, functional magnetic resonance imaging (MRI), and other methods. In another section of this article, some of the technical implementations interfacing to the human brain will be described, outlining the potential roles that nanotechnology could play in future approaches.

Finally, we will discuss ethical and philosophical considerations. How much do we *want* to interface with our brains? What should be the limitations? While medical progress leads to strong arguments for driving such technologies forward, it is also important to consider where the ethical borders lie. There remains ambiguity, as we have considered for millenia with technology; one can harness fire to warm one’s home or use it in a destructive way to burn down someone else’s house. Interfacing the brain requires strict ethical guidelines, which should be discussed before the technologies are fully available, and specifically before and while they are being developed. In addition, technical outlooks on related developments will be discussed, for example, how unique the brain is and whether artificial intelligence or computers in the future may exceed the capabilities of the human brain in a broad range of applications. The outline of this foward-looking review follows.

Improving Cell–Electrode Interfaces: Materials and Coatings

- Nanostructured Metal Electrode Arrays for Recording Electric Signals
- Nanowires for Template-Guided *In Vivo* Applications
- Carbon Nanotube-Based Neuronal Substrates and Scaffolds
- Graphene-Based Neuronal Interfaces
- Hydrogel-Based Interfaces
- Improving Biocompatibility *via* Surface Coatings
- Roles of Non-Neuronal Cells

Colloidal Nanoparticles as Transducers for Communication with Different Ion Channels or Neurotransmitters

- Different Gating Mechanisms of Ion Channels
- Nanoparticles as Transducers for the Stimulation of Ion Channels
- Photoelectric Stimulation Using Nanoparticles
- Photooptical Stimulation Using Nanoparticles
- Photothermal Stimulation Using Nanoparticles
- Magnetothermal Stimulation Using Nanoparticles
- Magnetomechanical Stimulation Using Nanoparticles
- Optomechanical Stimulation Using Nanoparticles
- Nanoparticles for Transducing Electrical Signals into an Optical Readout
- Nanoparticles and Nanomaterials for Transducing Chemical Signals
- Traversing the Blood–Brain Barrier
- Challenge of Specific Interfacing

Advanced Test Platforms to Model Aspects of the Brain

- Organotypic Slice Culture of Rodent Hippocampus
- 3D Organoids as *in Vitro* Brain Models
- Brain-on-a-Chip to Model Information Exchange between Brain Regions
- Nano- and Microfluidics with Tailored Porous Materials for Brain Interfacing
- 3D Printing toward Brain-on-a-Chip Structures

Interfacing the Human Brain: Technical Implementations

- Brain–Machine Interfaces (BMIs)—From State-of-the-Art to the Future
- Going beyond Classical Head-Mounted EEG Recording Devices
- Flexible Nanomaterial-Based Neural Interfaces
- Toward High-Throughput Recording Approaches
- Functional Magnetic Resonance Imaging (fMRI)
- Mapping Brain Neuronal Structure from the Nanoscale to the Whole Brain

Ethical, Philosophical, and Legal Considerations: Do We Want to Interface Our Brain and How Far Can We Go?

- How Do We Develop Approaches for “Neuroethics?”
- Good Scientific Practice: What Rules and Limits Should Be Considered?
- Some Thoughts about the Exploitation of Machine Learning in Bidirectional Brain–Computer Interfaces
- Will the Human Brain Be Outperformed by Artificial Neuronal Networks?
- Some Speculative (and Provocative) Thoughts about Interfacing Neurons with Traditional Medicine

Conclusions and Prospects

## Improving Cell–Electrode Interfaces: Materials and Coatings

In order to interface neurons with the external world, the interface between the neurons and devices for stimulation/readout is crucial. For intimate contact, these interfaces need to be tailored on the nanoscale. Here, we highlight several different approaches.

### Nanostructured Metal Electrode Arrays for Recording Electric Signals

Despite numerous advances in the fields of microelectronics and nanotechnology, it remains a challenge to record neuronal activity *ex vivo* continuously for real-time studies of neuronal networks. For example, patch-clamp electrophysiology remains the gold standard^41^ for electrophysiological characterization of single neurons,^42^ due to its low noise and high resolution, but suffers from a high workload, complex experimental procedures, and low throughput. In addition, macroscopic patch electrodes and the bulky experimental setup only allow for the possibility of measuring at a few sites of a neuronal network simultaneously, and the mechanical damage caused by contact with the electrodes limits the possible measurement times to a few hours. To record from numerous neurons in a network simultaneously over time without damaging the cells, microelectrode arrays (MEAs) have been developed.^24,36,43^ The MEAs have evolved over time, particularly in terms of spatial resolution, using sophisticated electronics. Compared to patch-clamp recordings, MEAs do not measure intercellular signals, but instead record extracellular signals, losing sensitivity and resulting in lower signal-to-noise ratios (SNRs). With MEAs, measurements have been expanded to several thousand channels.^44,45^

**Figure 1:**
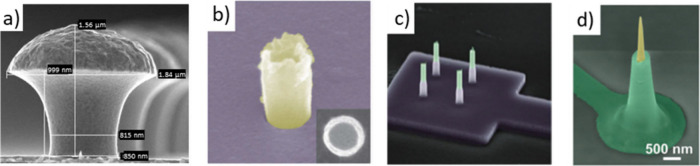
Nanoelectrodes made with different fabrication approaches and shapes. (a) Scanning electron microscopy (SEM) image of so-called gold-spine electrodes (adapted with permission from Hai *et al*.^67^ Copyright 2009 The Royal Society). The height of the structure is 1.56 μm. (b) SEM image of an iridium oxide nanotube electrode (adapted with permission from Lin *et al*.^74^ Copyright 2014 Springer Nature Limited). (c) SEM image of core–shell-type nanowires connected toward external contacts with encapsulated conductive lines (adapted with permission from Casanova *et al*.^75^ Copyright 2018 IOP Publishing Ltd). (d) SEM image of a silicon-based ultrasharp nanowire with an exposed Pt tip (adapted with permission from Liu *et al*.^76^ Copyright 2022 Wiley-VCH).

Many approaches have been taken to overcome some of the above-mentioned limitations. These strategies include the selection of electrode materials to reduce the impedance of the electrodes,^46−50^ and electrode geometries to improve the effective contact areas between the cells and the electrodes.^51−59^ Many groups have recently moved away from conventional two-dimensional (2D) electrodes to 3D electrodes fabricated using nanotechnology (see the section on “Advanced Test Platforms to Model Aspects of the Brain”). On the one hand, this change leads to larger surface areas and thus, reduced electrode impedance, which in turn leads to better SNR and higher specificity. On the other hand, when nanostructured 3D electrodes come in close contact with the membranes of micrometer-sized cells, highly localized interactions occur at the cellular nanointerface. By various mechanisms, cells can be spontaneously perforated by these nanoelectrodes.^60−63^ By applying physical stimuli such as mechanical forces, heat, or electric fields *via* the nanoelectrodes, highly localized interactions and associated stronger focusing of these stimuli on the cells are possible, which can be used to perforate the cell membrane. These nanoelectrodes have been fabricated from different materials, such as Au, Si, carbon nanotubes, indium tin oxide (ITO), titanium nitride (TiN),^64,65^ and graphene by bottom-up or top-down approaches,^66,67^ (see Figure 1). In addition to recording intracellular and extracellular electrophysiological signals, spontaneous or force-assisted perforation of cells with various nanostructures can be used to detect intracellular substances,^68^ for drug or biomolecular payload delivery,^69−72^ and real-time monitoring of intracellular biochemical signals.^73^

Ultrasmall and flexible devices have distinct advantages in terms of their size.^77^ They minimize invasiveness, can be fabricated in large numbers and densities, and thus significantly increase the number of recording sites.^53,78−81^ The use of planar technology allows for a wide range of materials and the structures can be made using nanotechnology. In particular, the improved understanding and development of nanobiointerfaces and strategies to insert nanostructures into the cell membrane also greatly improves the intracellular recording performance of nanodevices. Furthermore, the combination of nanoelectrodes with extracellular MEA^82^ structures enables easy parallel data acquisition with good signal quality, which can be extended to highly parallel platforms when combined with CMOS-based HD MEAs.^83^

Currently, nanoelectrode fabrication materials are mainly composed of biocompatible conductive, semiconductive, and insulating materials. Bottom-up synthesis techniques such as chemical vapor deposition (CVD),^84^ atomic layer deposition (ALD),^85^ and metal–organic vapor phase epitaxy (MOVPE) are used in the fabrication of these nanoelectrodes. Top-down fabrication strategies are used on bulk substrates that are formed or etched stepwise to obtain the corresponding nanostructures. In this process, masking with a target pattern is first performed by lithography, mainly using photo, colloid, lift-off, nanoimprint, electron beam (e-beam), and focused ion beam (FIB) processes.^86,87^ A dry etching process, typically reactive ion etching (RIE), is often then used to selectively remove the material from the substrate.^88^ This allows outstanding control of the nanoelectrodes to be fabricated. We also refer to the section “Advanced Test Platforms to Model Aspects of the Brain” for fabrication techniques.

Due to intrinsic noise in recording instrumentation, smaller electrodes result in lower SNRs, while larger electrode geometries cannot easily penetrate the cell membrane. However, the intracellular signals have large amplitudes compared to the extracellular signals, so better SNR can be expected. In addition, noise reduction can be achieved by strategies for surface enlargement of the nanoelectrode. It is known from many studies that the electrolyte-filled gap between the cell membrane and planar MEA electrode leads to low sealing resistance due to lose coupling, and consequently to low signal quality. Therefore, when designing the nanoelectrode geometry, one primarily tries to improve the coupling between cells and electrodes. Spira’s group designed a 3D electrode mimicking the shape of dendritic spines, which led to improved coupling between cell and electrode (Figure 1a^89^). Other groups made similar mushroom-like geometries from other materials.^82^ However, the 3D gold spine electrode^89^ had a large and round tip that was difficult to be enclosed by cells. To overcome this drawback and to gain access to the cell interior, one-dimensional (1D) nanomaterials or nanostructures (*e.g.*, nanowires and nanopillars) were subsequently developed and integrated into MEA electrodes.^60^ These sharp 3D nanodevices resulted in improved recorded signals compared with planar MEAs and allow temporary intracellular access after electro- or optoporation, but could not record full action potentials presumably due to leakage current. Cui’s group observed above 10 mV intracellular-like action potential (AP) recordings after electroporation, but that was attenuated to 30% of the original amplitude after 2 min and to 1% after 10 min, finally reaching an extracellular potential of 200 μV.^90^ Similar results were obtained by Park *et al*., who used a 4096 nanoelectrode array with CMOS control and recording electronics.^83^ Most promising for intracellular-like recordings are nanoelectrodes with small tip diameters and large aspect ratios.^91,92^ Deforming the plasma membrane is key to establishing intracellular access, which is also predicted by simulations,^93^ and indicates the importance of the interelectrode distance. The mechanical properties of the nanoelectrodes, which are largely determined by the material composition, also affect the response to cellular and external forces at the interface, as stiffer materials are more likely to rupture the membrane when force is applied, leading to intracellular access. The stiffness plays a role, especially in neuronal cells with their soft membranes.^94^

Most nanoelectrodes come almost exclusively into contact with the cytomembrane and generate mechanical forces at the cellular interface that causes cytomembrane curvature and subsequently activates curvature-sensitive proteins and controls actin polymerization and mechanotransduction in the intracellular space.^95^ Intracellular access is typically achieved by assisted penetration, which requires external factors such as chemical modification, electroporation, or plasmonic optoporation, while spontaneous penetration mainly depends on cell phagocytosis and cell adhesion and only happens for a limited fraction of nanoelectrodes.

**Figure 2:**
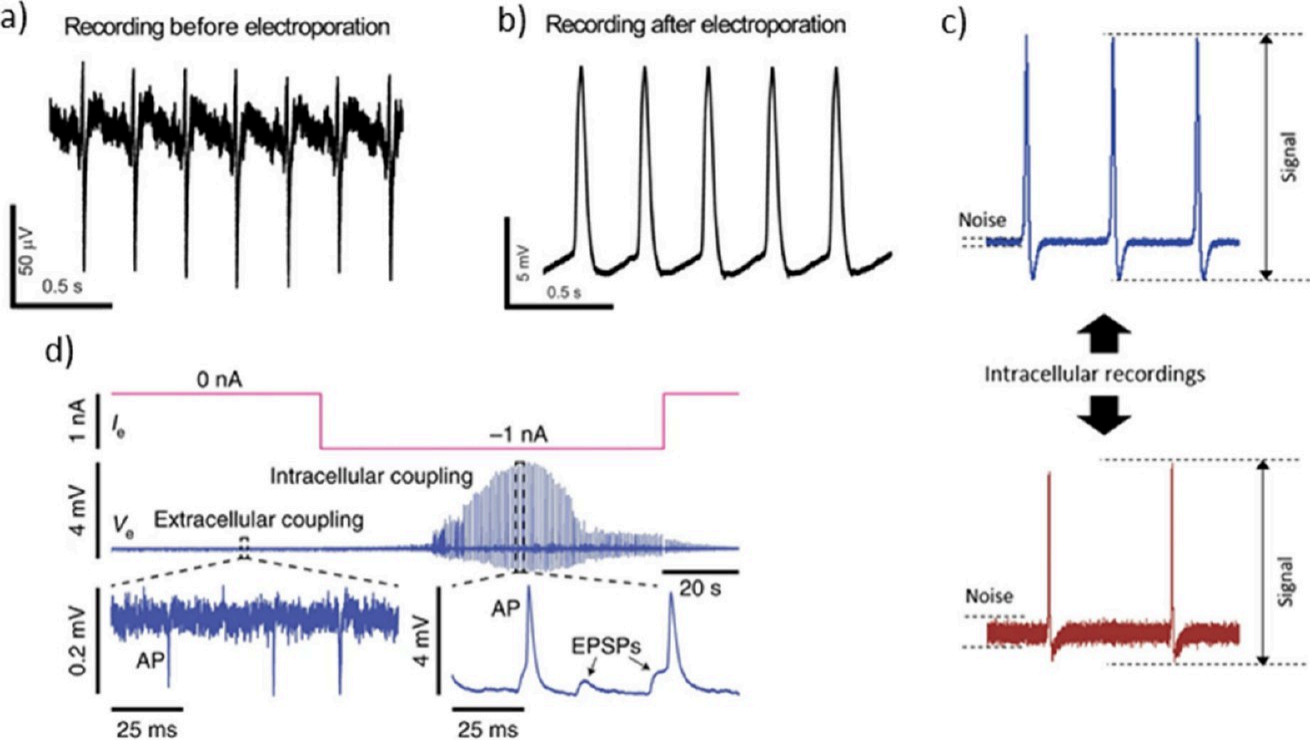
Example recordings from various groups with electro- and optoporation: Electrical recording from cardiac myocytes (a) before and (b) after electroporation. Image adapted with permission from Lin *et al*.^74^ Copyright 2014 Macmillan Publishers. (c) Electrical recording from cardiac myocytes after optoporation (upper graph) and electroporation (lower graph). Image adapted with permission from Dipalo *et al*.^97^ Copyright 2019 Wiley-VCH. (d) Extracellular recordings after electroporation. Excitatory postsynaptic potentials (EPSPs) and their triggering of an action potential (AP) are also visible. Image adapted with permission from Abbott *et al*.^83^ Copyright 2020 Springer Nature.

Assisted penetration can be based on chemical modification of the nanoelectrodes by phospholipids, leading to fusion with the cell membrane, as shown by the Lieber group.^96^ Alternatively, engulfment-promoting peptides (EPP)^67^ have been used with spine-like electrodes and allowed intracellular-like action potential recordings from *Aplysia californica* neurons. The use of chemical modifications for the intracellular access of nanoelectrodes is minimally invasive but still has low efficiency. As described above, typically several minutes after electroporation, this intracellular access disappears due to resealing of the cell membrane. Cui *et al*. employed nanotubes that delayed this resealing for up to 1 h (Figure 2).^74^ Alternatively, continuous current injection can maintain membrane pores, enabling subthreshold intracellular recording.^83^ These results show that although electroporation has significantly improved the efficiency of intracellular access, this penetration remains temporary and does not significantly improve the tight coupling between cell and nanoelectrode and the associated sealing. Furthermore, the electroporation pulse may interfere with the natural electrophysiological activities of the cells; the duration and signal quality are limited due to transient intracellular access, thus not leading to significant improvements in the electrode-cell interface. As an alternative to electroporation, a plasmonic optoporation technique has been developed by de Angelis by using 3D plasmonic nanoelectrodes excited by a short laser pulse to open transient nanopores in the cell membrane.^92^ This penetration allowed high SNR and intracellular-like recordings for more than 1 h (Figure 2).^92^ In this case, membrane pore formation occurs only at the tip of the electrode and has no effect on the cell-electrode seal, nor does it affect spontaneous electrical activity.

**Figure 3:**
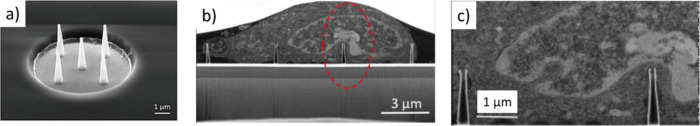
(a) Scanning electron microscope images of five nanostraws with 2 μm pitch on electrodes with a 6 μm diameter opening; the diameter of the nanostraws is on the order of hundreds of nanometers (adapted with permission from Shokoohimehr *et al*.^98^ Copyright 2022 Wiley-VCH). (b) Staining and resin embedding. (c) Inset of panel (b) [the red dotted region in (b) indicates the nanostraw on the right in (c)]. The fixed cells tightly engulf the nanostraws while the nucleus is being deformed at the tip of the nanostraws (unpublished images from the Offenhäusser group).

Recently, the concepts of vertical nanostraws (NS) were combined with that of nanocavity (NC) MEAs.^98^ Here, high-aspect-ratio nanostraws made of TiO_2_ were used to initiate tight cell-structure coupling, while the nanocavity significantly reduced the electrode impedance and therefore the noise levels of the electrodes. Scanning electron microscopy/focused ion beam (SEM/FIB) sectioning showed the deformation of the membrane as well as subcellular compartments (see Figure 3). The nanostraws extend into the cell for almost their entire length, with no areas of suspended cell membrane between them. Although the shape of the organelles may be deformed, their membranes appear to be intact, suggesting that the nanostraws do not damage structures such as the nucleus. Overall, both the restructuring of the cytoskeleton and the deformation of the plasma and nuclear membranes indicate close entanglement.^99,100^

**Figure 4:**
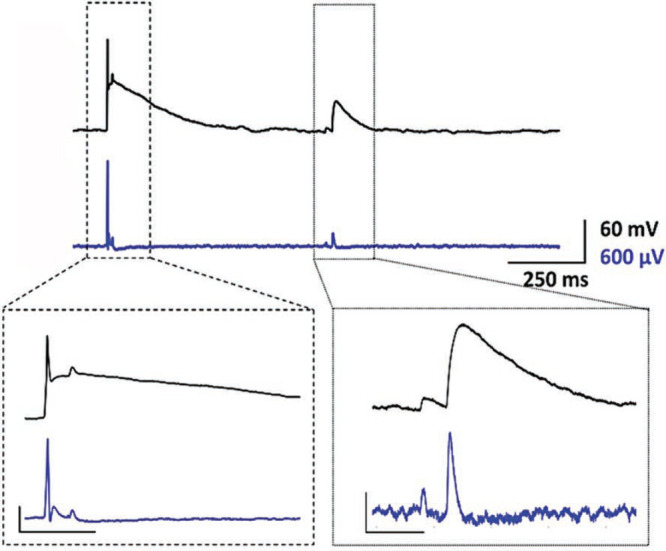
Simultaneous recording of the neuron’s activity using a patch-clamp electrode (black trace) and nanostraw–nanocavity–microelectrode array (NS-NC-MEA) (blue trace) focusing on a giant excitatory postsynaptic potential (EPSP) triggered by an action potential (AP) (left) and a spikelet (right). Bottom left: NS–NC–MEA detects distinct spikes that correspond to quenched and coinciding PSPs. Vertical scale bar corresponds to 40 mV (black) and 400 μV (blue). Time scale = 50 ms. Bottom right: details of postsynaptic potentiations (PSPs) in both patch-clamp and MEA traces. Amplitude scale is 20 mV (black) and 100 μV (blue). Time scale = 50 ms. Reproduced with permission from Shokoohimehr *et al*.^98^ Copyright 2022 Wiley-VCH.

These NS/NC-MEAs enable long-term recordings with increased signal amplitude without the need for external forces, such as optoporation or electroporation or surface functionalization, as discussed above. Furthermore, using simultaneous patch-clamp and MEA recordings, it is possible to record postsynaptic potentials (PSP). Smaller spikes were recorded with the NS-NC-MEA, which persisted throughout the on-chip patch-clamp measurements and matched patch-clamp recorded PSPs (Figure 4). Pairing of patch-clamp detected PSPs and MEA signals were robust, suggesting these spikes represent PSPs and not signals from neighboring cells. This work demonstrates that nonporated MEA recordings can consistently be combined with patch-clamp recordings to compare MEA-detected PSPs to ground truth.

### Nanowires for Template-Guided *In Vivo* Applications

The functionality of our brain relies on the reception and release of action potentials and associated neurotransmitters mediated by 10^15^ neuronal connections between *ca*. 86 billion neurons.^1,101,102^ Typically, planar Petri dishes and multiwell plates are used to perform model *in vitro* characterization experiments. Adhesion, proliferation, viability, migration, and guiding of seeded dissociated neuronal cells have been investigated and tested under the influence of the substrate’s chemistry and topography.^103−114^ Nanowires (NWs), quasi-dimensional NP objects with high aspect ratios are being explored extensively in such studies.^115−117^ By controlling the geometry, and exposed chemical functionality of the NWs and/or the arrays (pitch, type of ordering), the interactions between cells and NWs can be tuned.^93,118−120^ Nanostructuring of the electrode surfaces can improve the cell-electrode contacts, similar to the nanostructured MEAs discussion above. Cell membranes typically engulf the NWs, ultimately increasing the electronic seal resistance between the cells and electrodes,^53^ as for the case of carbon nanotubes (CNTs), discussed below. Such control of NW-neuron interactions enables tuning of adhesion, morphology, viability, and cellular outgrowth.^121−125^ The electrophysiological integrity of individual neurons and the ability to fire action potentials have been maintained on such NW substrates.^121,126−131^ Moreover, NW arrays can be used to elucidate the cell’s nucleus and the mechanical cell properties, to constrain and to guide cell movement, spreading, and cell polarization, and the outgrowth of neurites.^132−135^ Ongoing development in nanostructuring and device fabrication enable the incorporation of “active” components into the NWs, such as electrodes, optically active *p-n* junctions or open channels, and their exterior functionalization *e.g.*, with plasmid DNA, genes or bioactive compounds. Making the step from passive NWs to active arrays opens a plethora of target applications with high potentially impact, such as artificial cell transfection, drug delivery, electrical stimulation and sensing, photocurrent stimulation, and even optogenetics.^60,63,66,70,74,90,120,136−161^ Notably, some of the applications, were demonstrated mainly with model cell lines, such as HeLa or CHO cells, and not all have been yet been shown with neurons. However, the adaption of these procedures might open alternative therapeutic strategies for the local treatment of neurons without addressing the entire complex neuronal system.

**Figure 5:**
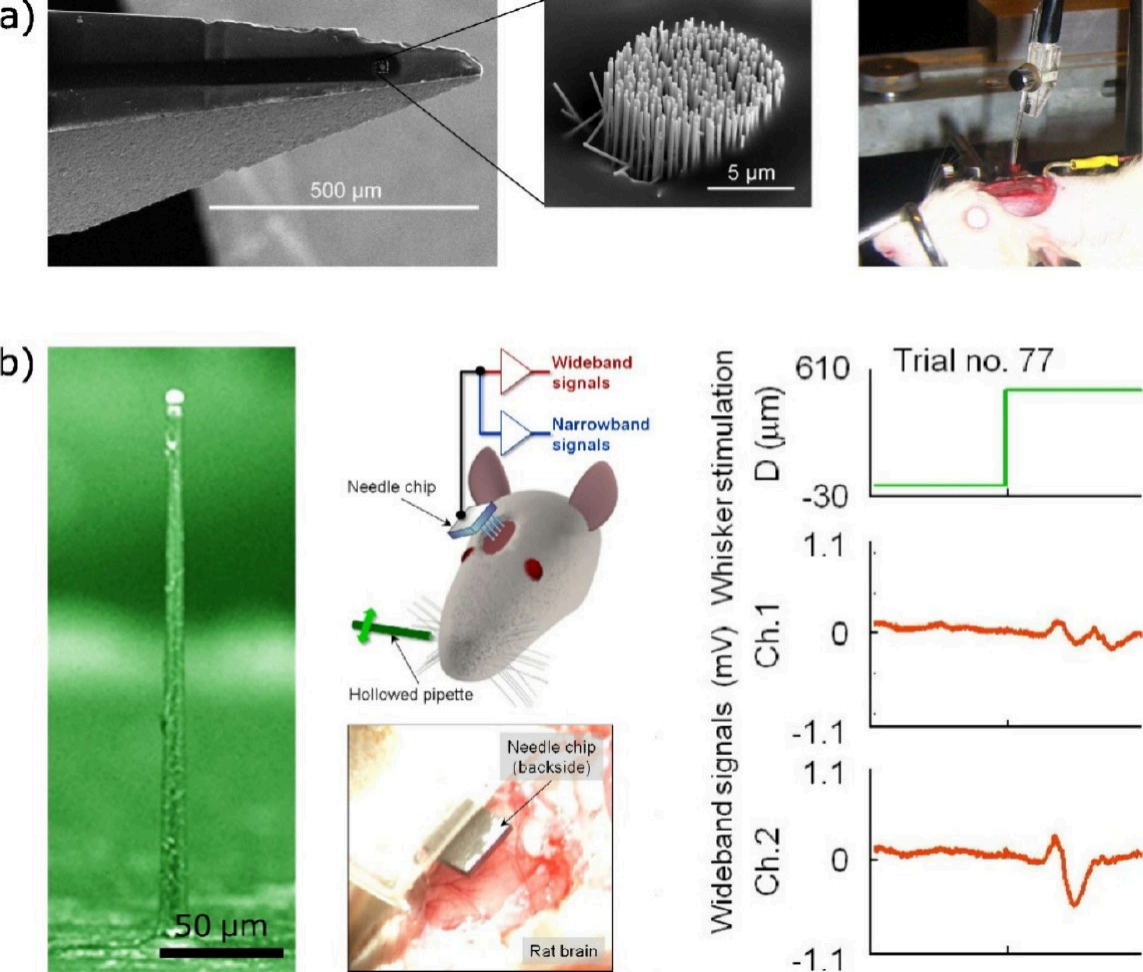
(a) Left to right: scanning electron microscopy (SEM) images of a macroscopic electrode with attached, freestanding GaP nanowires (NWs) (shown in magnification). Photograph of the NW-based electrode attached to a micromanipulator to enable *in vivo* neuronal recordings from the rat’s cortex. (b) False-colored SEM image of an individual (micro)needle from an array that has been used to electrically contact the left whisker barrel area in the somatosensory cortex of a rat (schematic and photograph middle column). On the right, the recorded and amplified wideband signal of the cortex after stimulating the rat’s whisker is shown. Images are taken and adapted with permission from Suyatin *et al*.^152^ and Fujishiro *et al*.^162^ Copyright 2013 PLoS and 2014 Springer Nature, respectively.

In addition to the plethora of *in vitro* studies and aforementioned applications, scattered attempts to exploit nanowires for interacting with neurons *in vivo* have been made. In particular, *in vivo* electrodes have been used to record signals in neuronal networks in the brain. Suyatin *et al*. showed that a metalized gallium phosphide (GaP) NW array prepared on a macroscopic electrode could be used to perform acute *in vivo* measurements of intracortical field potentials in the rat primary somatosensory cortex (Figure 5a). Furthermore, the authors of this study showed that the NW arrays have been rather robust and can withstand several implantation procedures.^152^ Free-standing, flexible silicon microneedles have been employed to record action potentials within the somatosensory cortex of a rat (Figure 5b). The importance of a small/thin wire geometry has been verified by immunohistochemical analysis. Specifically, the authors of this work showed that a decrease of the needle diameter significantly reduced the amount of damaged tissue, since fewer glial cells are activated and concentrated around the penetration location.^162^ Further reduction in diameter of the needles from a few microns down to the nanometer scale, as would be the case for long, free-standing nanowires, may further minimize damage to the brain and nerve tissues. When even thinner neuronal tissue, such as the retina, needs to be penetrated, microelectrodes on ultrathin polyimide and Parylene-C have been utilized, and minimal neuronal damage was demonstrated by immunohistochemical analysis.^163^ Silicon nanowires with transistor functionality have been developed and applied for neuronal interfacing.^164−166^ Ultimately scaled and specially coated by peptides,^167^ such nanowire transistor devices were utilized for intracellular recording of single cells.^168^

**Figure 6:**
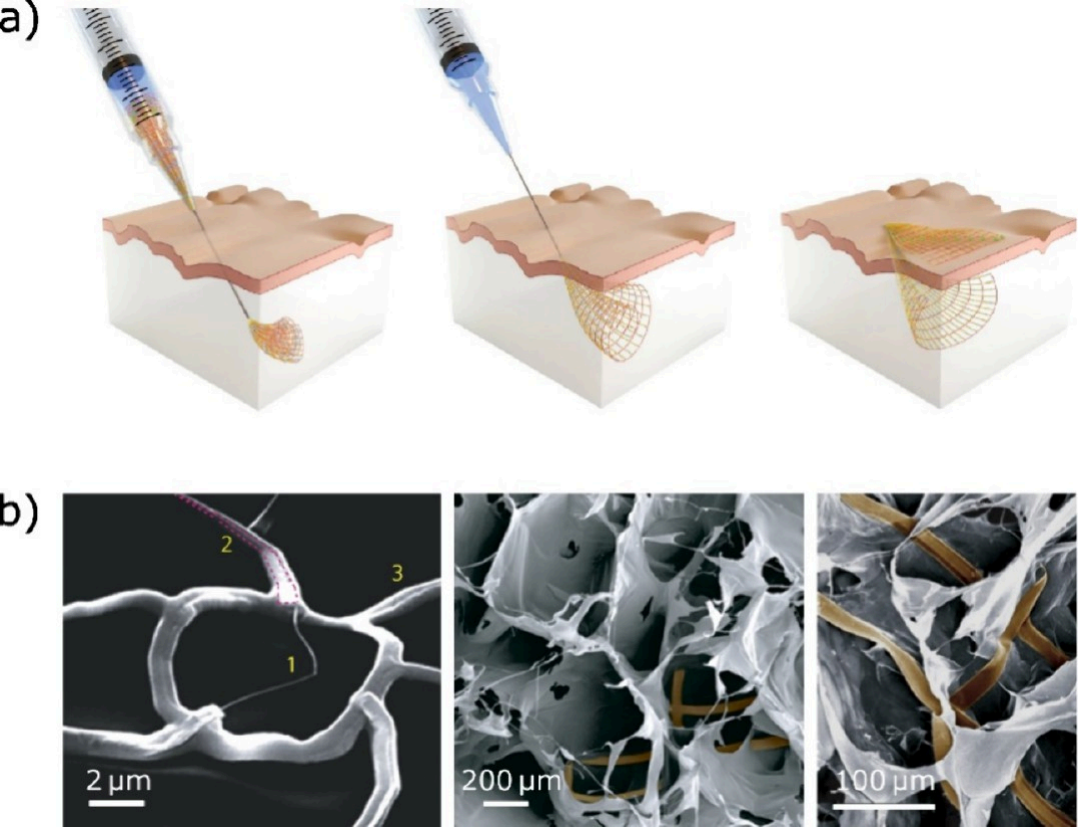
(a) Schematic depictions of an injectable, flexible nanowire network. (b) Left: Scanning electron microscopy (SEM) images of a kinked field-effect transistor nanowire (1), which is contacted by metallic interconnects (2) and supported by a polymeric SU-8 mesh (3) to form a nanowire nanoelectronic scaffold (nanoES). Middle and right: Hybrid nanoES device (false-colored in brown) based on an alginate scaffold. Images are taken and adapted with permission from Liu *et al*.^170^ and Tian *et al*.^166^ Copyright 2015 and 2012 MacMillan Publishers, respectively.

Recently, electrically contacted in-plane silver nanowire networks covered by indium–zinc oxide on flexible Parylene-C substrates have been reported to enable measurements of the neuronal activity from the rat’s cortex surface under urethane anesthesia.^169^ Such flexible NW-based electrodes could be principally injected into the lateral ventricle and the hippocampus of a brain by a syringe through the cerebral cortex, as shown by the Lieber group for mesh electrodes (Figure 6a).^170^ Specifically, instead of employing aligned or interconnected NWs for recording electrical signals from nerve tissue, individual field-effect transistor NWs have been implemented into biocompatible scaffolds, so-called nanowire nanoelectronic scaffolds (nanoES). Subsequently, these nanoES have been combined with collagen, alginate, and poly(lactic-*co*-glycolic acid) (PLGA) to form hybrid devices mimicking the natural tissue structure (Figure 6b).^166^

Devices based on individual long carbon-based wire electrodes could be used to record signals deep from the brain as demonstrated for long carbon nanotube (CNT) fiber electrodes (see also above).^171^ The electrode stiffness can be controlled by embedding the CNTs into a microfluidic device. The small footprint, here in the μm-regime, but scalable for CNTs down to a few nanometers, of such devices minimizes the injury caused to the tissue and hence reduces acute hemorrhage and neuroinflammatory response.

Besides the “passive” recording of signals *in vivo*, the same electrodes could be used to evoke responses electrically, *e.g.*, the release of action potentials, at specific regions in the brain. Note, MEAs are a working horse in *in vitro* studies of neuronal cell cultures to measure but also to stimulate neuronal signals. Conventional MEAs generally consist of up to thousands of microelectrodes arranged in a square grid; each electrode is connected to a single channel and can be read out individually. On the one hand, the electrodes, on which the cells rest, do not have to be planar but could be micro-^82^ and nanostructured with vertically aligned wires (see also below for the case of CNTs).^76,172,173^ On the other hand, several thousands of electrodes can be prepared and addressed by utilizing complementary semiconductor-metal-oxide (CMOS) technology.^174−177^ With such high-resolution arrays, the propagation of action potentials after electrical stimulation even along an individual axon can be recorded and tracked *in vitro*.^178^ Further, some recent preparation methods allow for the processing of biocompatible, flexible MEAs, which have been applied *in vivo* to measure the electrocorticogram of a rat.^179^ Combining all aspects and expertise gained on planar MEAs with nanostructured vertically aligned nanowires as electrodes, with its inherent reduced inflammatory properties, could potentially build the bridge to brain–machine interfaces.

**Figure 7:**
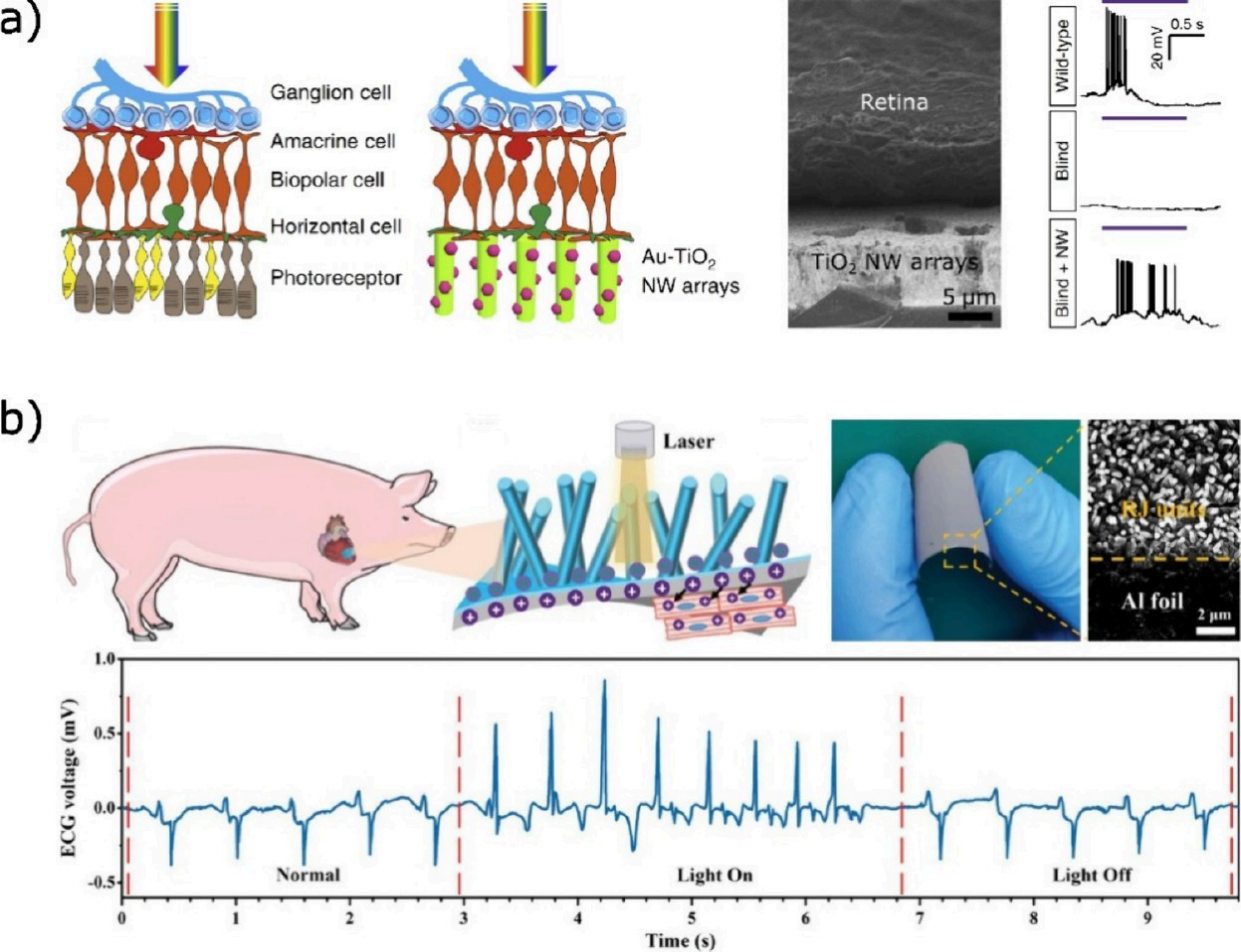
Nanowire (NW)-mediated light excitation of neuronal cells in (a) the retina or (b) heart muscle cells. (a) From left to right: Schematics of the replacement of biological photoreceptors in the retina by Au-decorated TiO_2_ NW arrays. Scanning electron microscopy image of the retina in contact with the NW array (scale bare 5 μm). Whole-cell patch-clamp recordings of the retinal ganglion cells (RCGs) upon UV light recording for wild-type, blind, and blind retinas in contact nanowires. (b) Upper row from left to right: Schematics of the NW implant at the porcine heart. Photograph of the flexible device consisting of an aluminum foil on which radial junction (RJ) nanowires have been grown. Lower panel: Heartbeat as a function of the light irradiation of an implant. Images are adapted with permission from Tang *et al*.^153^ and Liu *et al*.^145^ Copyright 2018 Springer Nature and 2020 Wiley-VCH, respectively.

By further increasing the complexity of the individual NWs’ structure, *e.g.*, by adding NPs or by implementing a *p-i-n* junction, photoactive NWs in contact with neuronal cells could be used to optically trigger a response of the muscle or nervous system. As an example, gold-decorated TiO_2_ nanowires acting as artificial photosensors have been brought in contact with a degenerated retina–without natural photoreceptors–of mice. Incoming green, blue, and UV light led to stimulation of retinal ganglion cells by the NWs. As a result, NW array devices implanted in a subretinal fashion led to activation of the primary visual cortex upon light irradiation and improved response in the pupillary reflex (Figure 7a).^153^ In another example, silicon NW-based photodiodes have been implanted as subretinal prostheses in rabbits.^180^ Visually evoked action potentials upon visible light irradiation of the NW prosthesis revealed the potential of vision restoration by such NW implants. Similarly, Liu *et al*. showed that radial *p-i-n* junction Si NWs on a flexible substrate could be implanted in a porcine heart (Figure 7b).^145^ By modulating the incoming light, the pace of the heartbeat can be reversibly controlled. In summary, NWs as electrodes have the potential to be game-changing in establishing brain-electronic circuit interfaces on single-cell levels. Especially the ability not only to trigger electrically and to record signals, but also potentially to convert external stimuli, such as light (see the aforementioned references), in nanosized photoelectrodes or strain in piezoelectric nanowires,^181^ in neuronal signals could enable such implanted NW-based devices to restore human senses lost due to injury or disease. Whether the transition of these techniques to clinical use is possible, and how long it may take, remain open questions.

### Carbon Nanotube-Based Neuronal Substrates and Scaffolds

The combination of nanotechnology and neurobiology fueled high expectations for innovative and successful therapies in numerous diseases and in the design of implantable hybrid microsystems able to help in healing injured neuronal tissue.^182−185^

Carbon nanotubes (CNTs) have extraordinary electronic, mechanical, and thermal properties that arise from their nanoscale dimensions, together with their structure of graphene-like sheets. The way in which the sheet is rolled on itself, thus the geometric structure and disposition of the hexagons along the surface, defines the diameter and the helicity of the obtained CNT, key features that determine their electrical behavior as metal or semiconductors.^186,187^ Moreover, CNTs can be easily fabricated from the molecular to the nano- and the micrometer scales,^188^ which allows for tuning their biocompatibility and toxicity.^189^ More specifically, neuro-medical applications with CNT-based components, may provide revolutionary tools to interact with the central nervous system (CNS) and, indeed, enable targeting of neuronal physiology from the functional (electrical) point of view.^190−195^

CNTs have been utilized as coating materials for metal microelectrodes to lower electrode impedance^196^ and to promote neuronal cell adhesion.^197^ Neuronal cells preferentially adhere to CNTs and form connected clusters with strong neuronal activity.^196^ Such devices can be manufactured as CNT-Au composite materials,^198^ or can be realized by using CNT materials alone as electrodes, as discussed below.^193,199,200^

#### Carbon Nanotube-Based Substrates

**Figure 8:**
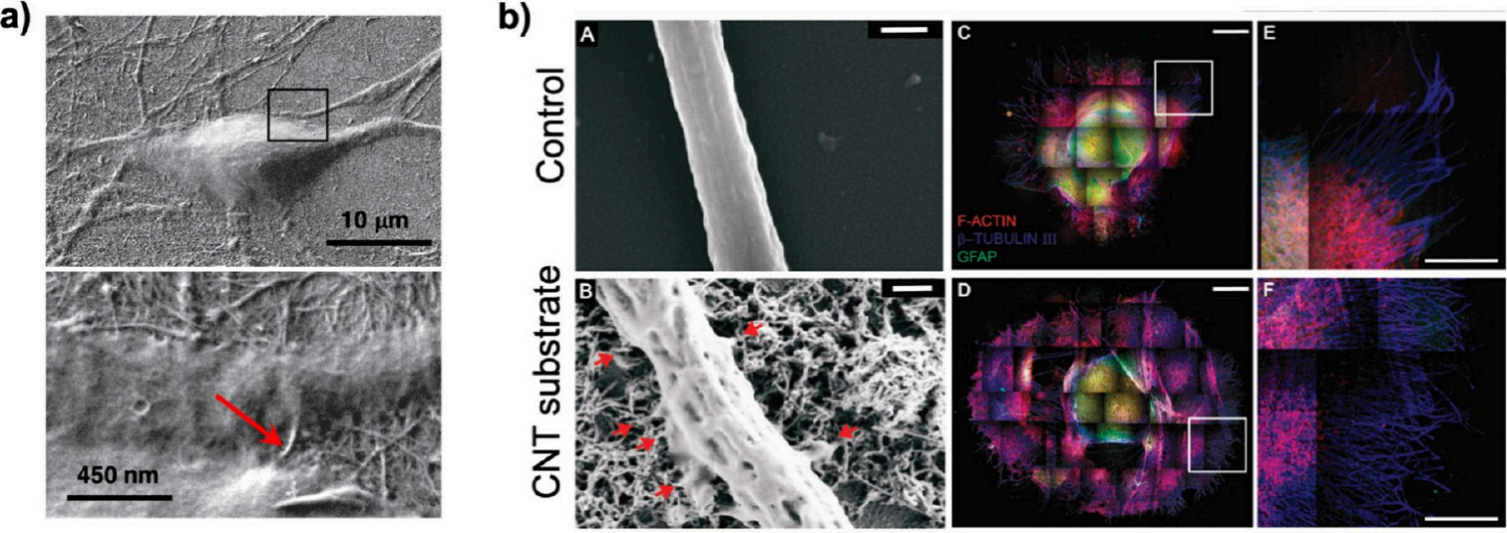
Characterization of carbon nanotube (CNT) substrates and ultrastructural interaction between CNTs and cultured neurons. (a) Scanning electron microscopy (SEM) images of cultured hippocampal neurons on CNTs grown for 10 days *in vitro* (DIV). Note the healthy morphology of the neurons and the outgrowth of neurites attaching to the CNT surface. At higher magnifications, the intimate contacts between bundles of CNTs and neuronal membrane are observed. Adapted with permission from Mazzatenta *et al*.^215^ Copyright 2010 Society for Neuroscience. (b) Organotypic spinal cultures: impact of multiwalled CNT (MWCNT) interfaces on neurite outgrowth. (A) SEM image of a peripheral neuronal fiber of a control spinal explant grown on glass. Scale bar: 500 nm. (B) Scanning electron microscopy (SEM) image of a spinal explant peripheral neuronal fiber on a CNT substrate; note the tight and intimate contacts (red arrows) between the neurite membrane and the MWCNTs. Scale bar 500 nm. (C,D) Confocal microscopy image reconstructions of spinal slice cultures at 8 DIV under control and CNT growth conditions, respectively. Immunofluorescence of specific cytoskeletal components, F-actin, β-tubulin III, and glial fibrillary acidic protein (GFAP). Note the β-tubulin III positive neuronal processes radially exiting the growth area in both cultured explants. (E,F) High-resolution confocal magnifications of the framed areas highlighted in (C) and (D), respectively, visualize the bundles of fibers emerging from the growing belt located around the slices. (C–F): green, GFAP; red, F-actin; blue, β-tubulin III. In (C–D): scale bar 1 mm. In (E,F): scale bar 500 μm. Adapted with permission from Fabbro *et a*l.^205^ Copyright 2012 American Chemical Society.

CNTs and other graphene-based materials have been investigated as substrates to interface with neuronal circuits.^201^ In such cases, the development of hybrid neuro-nanomaterial networks served as a platform to challenge neuronal sensing of, and responding to environmental physical and chemical features. An important *in vitro* study was published by Matson and co-workers who reported the high biocompatibility of CNTs substrates for neuronal growth, and how CNTs may promote the growth of neurons and extension of their axonal processes in all directions.^202^ Lovat *et al*. demonstrated that CNTs, when interfacing with neuronal growth, are able to boost interneuronal communication, increasing postsynaptic currents and action potentials in hippocampal neuronal networks interfaced to CNTs mats.^203^ Moreover, the balance between inhibitory and excitatory components in the neuronal network was not affected. Multiple studies have shown the impressive ability of CNTs to modulate neuronal behavior at the structural (synaptogenesis and neurite elongation) and functional (synaptic efficacy and action potential propagation) levels and even the potential to induce neuronal differentiation.^204−210^ From then on, interfacing neurons with CNTs emerged as an effective tool for manipulating neuronal activity at multiple levels of tissue complexities, that is, at the single neuron, synaptic network, and multilayered tissue levels.^211−214^ Now, it is thought that the recipe for such successful integration of CNTs to neuronal networks comes from the physical interfaces along the cylindrical morphology of the nanomaterials and the effects on the electrical activity of the neurons due to direct electrical contact with the conductive nanomaterials.^215,216^ By means of single-cell electrophysiology techniques, electron microscopy analyses and theoretical modeling, it has been hypothesized that CNTs can provide a “shortcut” between the proximal and distal compartments of neurons.^217,218^ This hypothesis, supported by observations that neuronal membranes establish tight contacts with CNT substrates as shown in Figure 8a, was further corroborated by experiments where, when cells were forced to fire trains of action potentials, reverberations after depolarizing potentials were detected. These were much more frequent on CNT deposited cells than those grown on inert glassy supports. In summary, neurons coopt CNTs to tune genuine biological processes related to their regenerative ability.^217,218^

Synapses are the subcellular structures that physiologically interface one neuron to another; their formation is nearly doubled in the presence of CNT substrates.^218^ Synaptic plasticity, a striking feature of neuronal transmission that governs the processing abilities of the CNS by tuning the short-term dynamics of connections, is also affected: synapses developed on CNTs exhibit short-term potentiation and thus display transient augmentation in strength when activated repetitively, sustaining high-frequency flows of information. This enhanced functionality is attributed to the conductivity and physicochemical properties of the CNTs.^217,218^

#### Three-Dimensional Carbon Nanotube-Based Sponges

**Figure 9:**
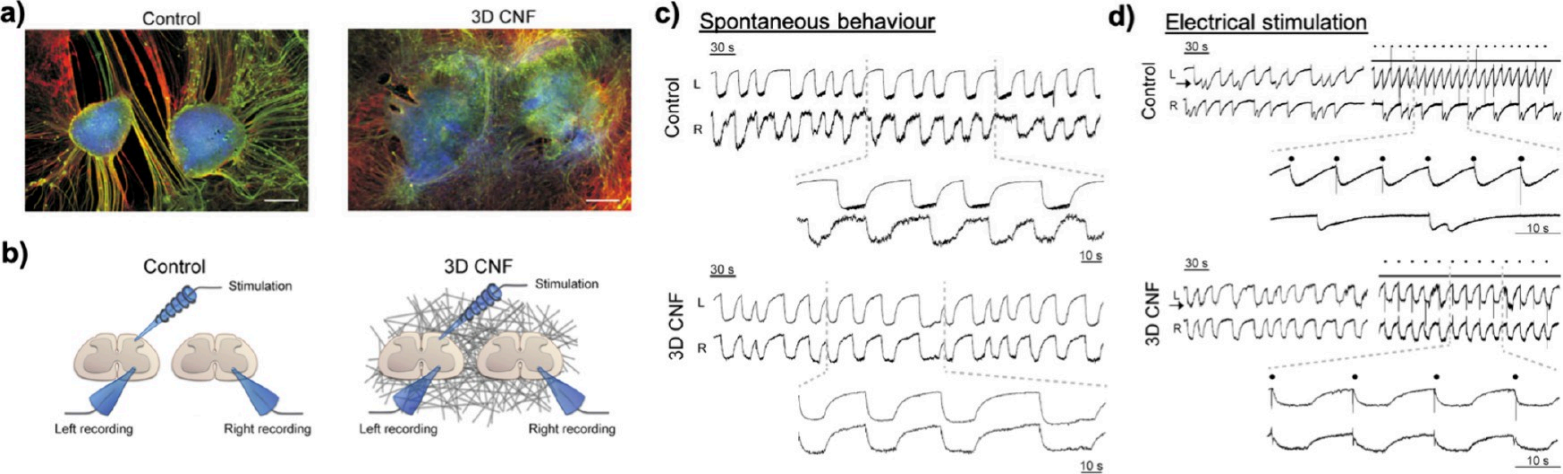
Three-dimensional carbon nanotube-based sponges (3D CNFs) guide the functional reconnection of ventral outputs in segregated spinal organotypic slices, cocultured in “Control” and in 3D CNFs after 14 days of growth. (a) Immunofluorescence is displayed for neuron-specific microtubules (b-tubulin III; red), neurofilament H (SMI-32; green), and nuclei (DAPI; blue). Scale bars 500 μm. (b) Sketch of the experimental setting for double-slice ventral recordings and dorsal stimulation. (c) Local field potential bursting induced by strychnine and bicuculline recorded simultaneously from left (L) and right (R) slices in Control and 3D CNF. (d) Bursting local field potentials (LFP) entrainment by dorsal electrical stimulation (dots) of left slices (arrow) in Control and 3D CNF slice pairs. Adapted with permission from Usmani *et al*.^221^ Copyright 2016 The Authors.

The impact of CNT interfacing substrates on CNS tissue has been tested by coculturing spinal cord (SC) and dorsal root ganglia (DRG) multilayered explants.^205,219^ With respect to controls, spinal explants cultured on CNTs displayed higher numbers of longer axons growing in tight contact with the substrate (see Figure 8b). These neuronal processes grown on CNTs, when assessed by atomic force microscopy (AFM), were less stiff than in controls and seemed to conform to the CNT carpet increasing their contact surface and apparently integrating CNTs as supports or exoskeletons.^220^ Furthermore, as shown in Figure 9, morphological analysis suggests that critical to the effects of pure 3D CNT-based sponges (named 3D CNF) is the ability of these scaffolds to guide the 3D random morphology of outgrowing spinal explant neurites in the third dimension; the conductive properties of the scaffolds may mediate direct electrical transmission between cultured slices.^221^ The overall interactions of the spinal tissue with the substrate appear to be intimate and reminiscent of what has been reported for isolated cells in culture.^219,221^ When sensory afferent pathways, preserved in spinal explants *in vitro*, were exogenously activated, the resulting synaptic responses recorded from the explants not in direct contact with the CNT layer were strongly increased and synchronized. This observation indicated that the boosting effects of CNTs at the interface were transferred from the layers of neurons directly exposed to the CNTs to those functionally connected, yet physically far from the interface.^205,219,221^

**Figure 10:**
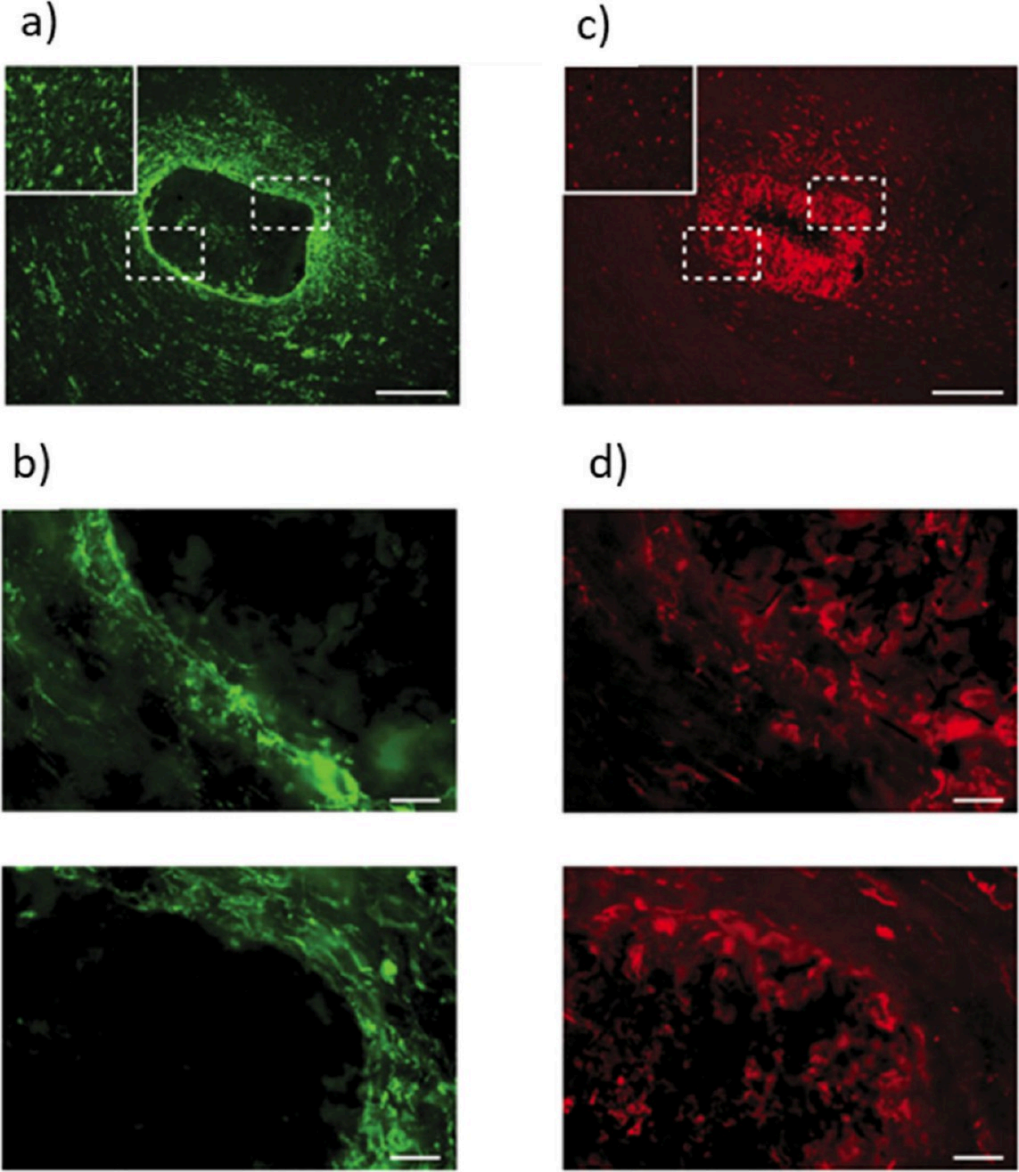
Tissue reaction to carbon nanotube-based sponges (CNFs)-based scaffolds implanted into the adult rat visual cortex as visualized by immunostaining of glial fibrillary acidic protein (GFAP) and Iba1. GFAP is a marker for reactive astrocytes, and Iba1 is a marker for microglial cells, the resident immune cells of the central nerves system. (a) GFAP-positive cells (green) are found surrounding the implant and within the material; the boxed areas indicate high-magnification images shown in (b); Inset, contralateral hemisphere used as a control. Scale bar 200 μm. (b) High magnification of GFAP reactivity at the implant edge demonstrating the minimal and irregular cellular localization around the scaffold. Scale bar 50 μm. (c) Iba1-positive cells (red) are dispersed consistently throughout the tissue and within the material; the boxed areas indicate the high-magnification images shown in (d); inset, contralateral hemisphere used as a control. Scale bar 200 μm. (d) High-magnification images of the ionized calcium-binding adapter molecule 1 (Iba1) reactivity demonstrate no obvious border at the implant edge to indicate scar formation. Scale bar 50 μm. Adapted with permission from Usmani *et al*.^221^ Copyright 2016 The Authors.

The robust interactions between CNTs and neurons have been reported in a number of studies.^215,220^ Chronically implanted ultrasmall electrodes, *i.e.*, of subcellular size, coated with CNTs, have improved signal-to-noise ratios and enhanced stability over months to years, compared to traditional microelectrodes, such as metal-based devices and nondry electrodes, as hydrogels, polydimethylsiloxane (PDMS)-based and liquid silicone rubber.^193,222−224^ The CNTs present large surface areas that decrease the impedance and enhance the charge transfer without inducing inflammatory and immune reactions, while apparently diminishing the astrocytic reaction and glial scar formation.^58,225^ Such enhancements significantly reduce the sizes of the implantable devices and preserve their electrical performance, thus improving recording and stimulation for long periods.^226,227^ Given the beneficial CNT interfaces with neurons, CNT-based electrode arrays also facilitate the anchoring of neurons directly and only onto the electrode sites, avoiding the use of external chemical treatment.^228,229^ These beneficial effects have been observed for the neuronal recording and stimulation of *in vitro* culture,^230,231^ as well as *in vivo* implantation, including intracortical electroencephalograph (EEG) measurements,^222,231,232^ cochlear attachment to restore hearing in the inner ear, EEG monitoring (see Figure 10),^223,233,234^ dorsal root ganglion stimulation,^58^ and sciatic nerve recordings.^235^

From a bioengineering perspective, the CNTs’ intrinsic characteristics make them important building blocks for the production of conductive materials. Composites with CNTs enrich polymers with electrical conductivity and enhanced mechanical properties.^236−242^ Likewise, the presence of a matrix fixes the CNTs spatially, enabling fabrication of robust 3D architectures enriched by nanotopography, which stabilizes interfaces with living tissues. The resulting 3D hybrid materials are useful for implantation, neuronal growth, and axonal regeneration, with significant advantages over polymers alone.^243^ The architecture of the scaffold structure, together with the micro- and nanotopography and alignment at the cellular level, are crucial for successful impact on neuronal regeneration. In particular, the longitudinal tubular shape of CNTs, which mimics the natural structure of the axonal pathways within several areas of the CNS,^244^ provides physical guidance for axonal regrowth and cell migration, and thus may enhance nerve regeneration.^245−248^ As a proof of principle, a hybrid synthetic/cellular construct based on a PDMS porous matrix and CNTs as substrate was developed for culturing dissociated cells of rat hippocampus. The resulting scaffold led to neuronal colonization and 3D synaptic network reconstruction while remaining sufficiently soft to match the viscoelasticity of native hippocampal tissue (Figure 9).^221,249^ The CNTs improve and boost neuronal functionality in three dimensions. Such 3D cellular organization is able to induce neuronal network outputs that differ strongly from the more typical two-dimensional (2D) constructs. Finally, in a test of the nature of the materials, Roberts and co-workers demonstrated how primary embryonic rat motor neurons grown on thin films of alternating stripes of horizontally aligned CNTs and SiO_2_ selectively migrate toward and adhere to the CNT-containing portion of the substrate.^250^

**Figure 11:**
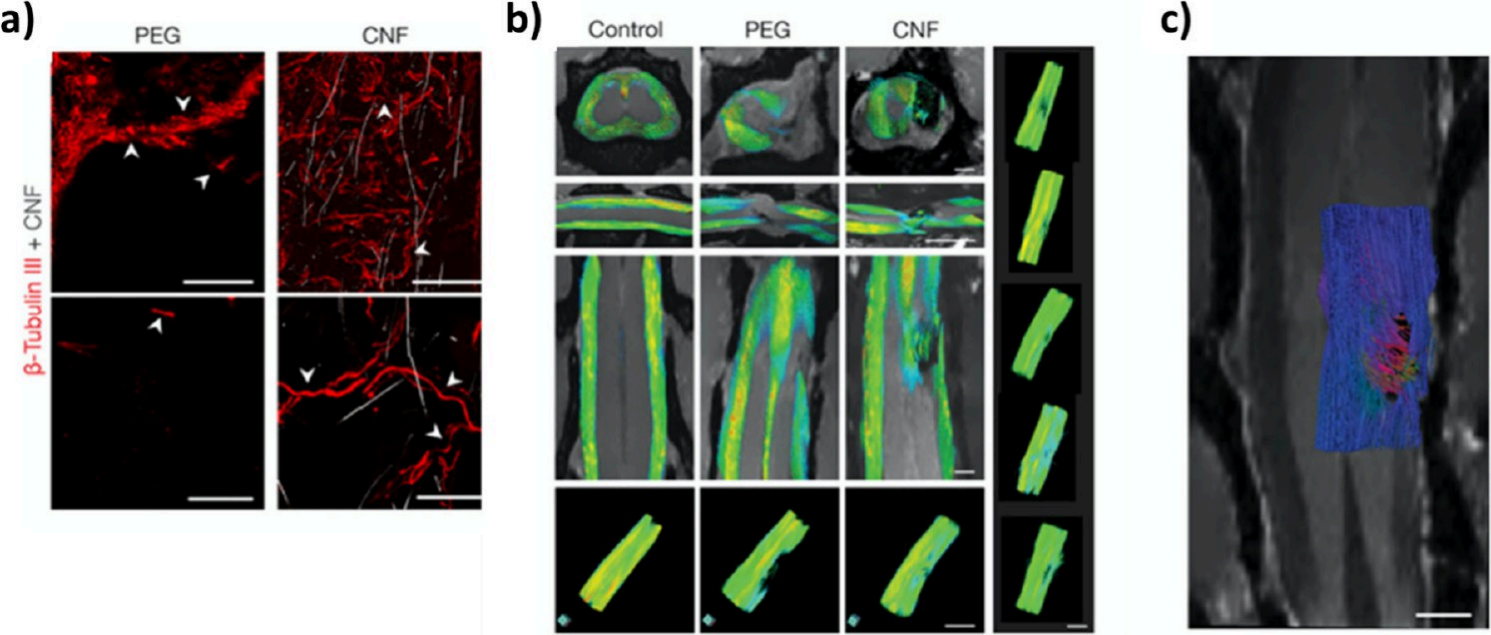
Carbon nanotube-based sponge (CNF) supports implantation in spinal cord injury animal models. (a) Confocal micrographs detail the lesion site at low (top) and high (bottom) magnification. Arrowheads indicate shredded remains and fibers in poly(ethylene glycol) (PEG) (left) and tortuous axons within the CNF (right). Scales top (left and right), 100 μm; bottom (left and right), 25 μm. (b) Fiber tracks in aged-matched naïves (Control) and spinal cord injury (SCI) (PEG and CNF) at 5 to 6 months after surgery, with fractional anisotropy (FA) values ranging from FA = 0 (in blue) to FA = 1 (in red). Right column: 3D representations of fiber tracts of five different examples of 5 to 6 months carbon nanotube (CNT)-implanted animals. Scale bars, 2 mm. (c) Fiber tracking analysis of diffusion tensor imaging (DTI) data constructed along the implant area of a CNF-treated rat (6 months post-SCI; only half spine presented to facilitate visualization) with the 2D MRI coronal plane through the implant. Colors represent fiber orientation following the conventional code for tensor directionality (blue, anterior–posterior; red, left–right; and green, dorsal–ventral directions). Scale, 1 mm. Adapted with permission from Usmani *et al*.^251^ Copyright 2020 The Author(s).

Pure CNFs were constructed and implanted in preclinical spinal cord injury (SCI) rat models.^251^ After six months postlesion implantation, the scaffolds showed not only the ability to adapt and to connect with the physical and electrical properties of the CNS tissue, thus sustaining spinal displacements with CNF integration into the spinal tissue, but also the potential to enhance axonal regeneration and rewiring, which is fundamental to re-establishing motor control after injury (see Figure 11). These data suggested that CNFs induced faster and improved locomotor long-term recovery in comparison to the control (*i.e.*, CNF-free) group. Thus, the CNT scaffold played an important role in the improved motor behavior. Confocal microscopy analysis plus fiber tracking by magnetic resonance imaging (MRI) and neurotracer labeling of long-distance corticospinal axons suggest that such recovery of motor functions is attributable to crossing of the lesion gap by regenerating fibers. Furthermore, CNF implants showed long-term biointegration, *i.e.*, invasion of neuronal fibers and blood vessels, with limited tissue reactivity.

#### Conductive Polymer–Carbon Nanotube 3D Porous Composites

**Figure 12:**
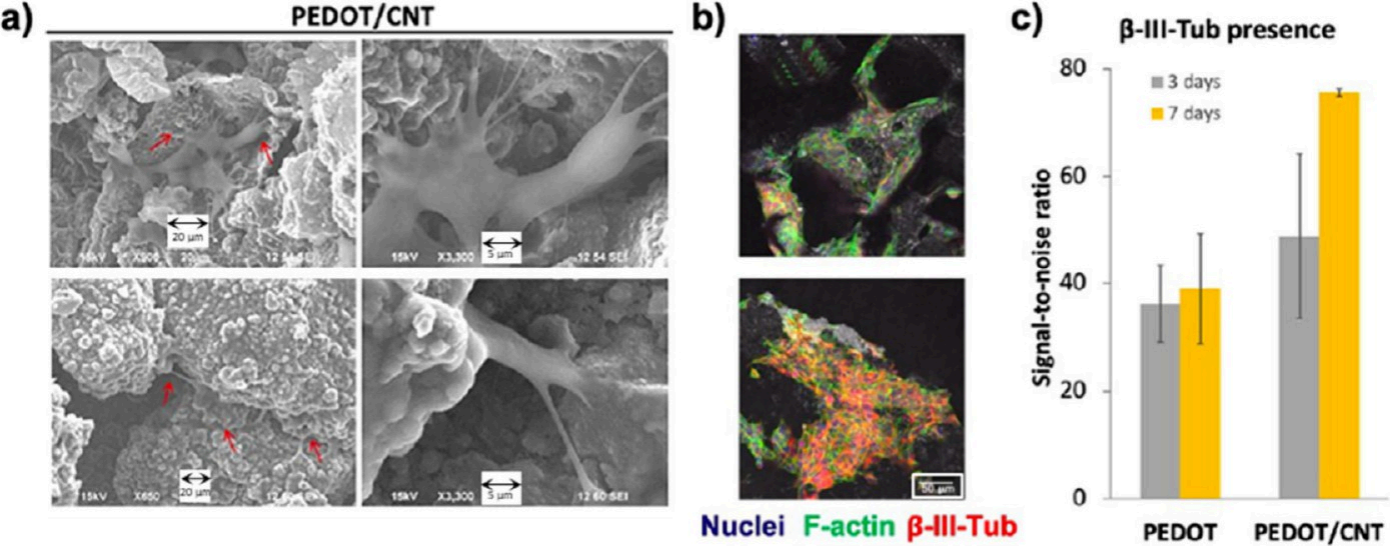
(a) Scanning electron microscope images of SH-SY5Y cells grown on poly(3,4-ethylenedioxythiophene)/carbon nanotube (PEDOT/CNT) scaffolds after 3 (top) and 7 (bottom) days of culture (DIV). The red arrows indicate cells. The scale bars for the images on the left and right correspond to 20 and 5 μm, respectively. (b) β-Tubulin class III and f-actin staining of SH-SY5Y cells grown on PEDOT and PEDOT/CNT scaffolds after 7 DIV. The scale bar represents 50 μm. (c) Amount of β-IIITub expressed in terms of “signal-to-noise ratio” of the incubated cells. Adapted with permission from Dominguez-Alfaro *et al*.^258^ Copyright 2020 American Chemical Society.

Conductive polymers downregulate glial reaction without affecting neuronal viability and function.^252^ To leverage these interactions, highly conductive 3D porous composites formed exclusively of polypyrrole (PPy) or poly(3,4-ethylenedioxythiophene) (PEDOT) and CNTs were constructed,^55,58,193,253−255^ which were able to act as electrodes themselves, with recording and stimulating capabilities. They support neuronal growth and regeneration more than nonconductive or CNT-free matrices.^256,257^ Moreover, tests with *in vitro* cultures of neuroblastoma SH-SY5Y cells indicated biocompatibility. The presence of high amounts of β-tubulin class III and MAP-II target proteins, which are predominantly expressed in neurons, suggests differentiation into neuronal cells after a week of incubation and without the need for additional chemicals during the differentiation process, as in common protocols (Figure 12).^258^ The design of electrodes based on conductive polymers in brain–machine interface technology offers the opportunity to leverage variably manufactured materials to reduce gliosis, the most common brain response to chronically implanted neuronal electrodes.^259−262^ Conductive polymers, with finely tailored physicochemical properties, may result in electrodes with improved adaptability for the neuronal tissue.^77,263^ MEA devices with PEDOT/CNT coatings to reduce input impedance are commercially available.^264^ Other combinations of nanomaterials with conductive polymers are also being evaluated and optimized for optoelectronic interfacing of neuronal cells and tissue.^265^ Intimate contact between the neurons and graphene is beneficial for neuronal activity, presumably by maximizing graphene-ion interactions.^266^ Engineering graphene-based materials to assemble out-of-plane structures, could be exploited for neuronal interfaces.^267,268^ We anticipate that also other material combinations between polymers, NPs and graphene-based materials for optimized neuronal interfacing will be developed and tested.

One of the considerable challenges in neurotechnology is the development of scaffolds for spinal cord reconstruction. Exploiting the spinal microenvironment, including its physical properties is critical. CNTs are promising for this application. Organotypic spinal slice cultures are complex *in vitro* models in which both, sensory-motor cytoarchitecture and electrical properties, are retained in 3D. Spinal slices separated at distances greater than 300 μm fail to reconnect under basal conditions. Pure CNT and PDMS/CNT 3D scaffolds were used to reconnect separated spinal explants *in vitro*.^269^ The incorporation of CNTs led to the adhesion of axons to the devices, favored the formation of intricate networks of axons regrowing from the interfaced spinal tissues, promoted more efficient regenerating interfaces, and conferred increased neuronal activity in these freshly developed networks. The scaffolds guided regrowing axons toward functional reconnection of separated spinal explants. These constructs were subsequently implanted into the visual cortex of adult rats and only minimal immune response surrounding the implants at 2, 4, and 8 weeks after surgery were observed.^269^

Graphene is useful for recording and stimulation from neuronal cells.^270^ Ultraconformable electrodes on flexible substrates can be realized.^77,271^ Other forms of graphene-based materials can be utilized as electrode materials for neuronal interfacing and we refer interested readers to a recent review.^272^ Artificial scaffolds have been proposed as a prototype for providing an artificial extracellular microenvironment that guides neuronal regrowth *in vivo*, exploiting the physics governing spinal regenerative plasticity. Such materials have therapeutic potential for the treatment of SCI. In addition to using 3D hybrids, CNTs, and graphene-based materials in (nano)engineered regenerative interfaces, alternative approaches are discussed in the section on “Advanced Test Systems”.

### Graphene-Based Neuronal Interfaces

Current neuromodulation therapies are not based on stimulating single neurons, which macroscopic electrodes are unable to do. The sheer size of neuronal implants and electrodes currently in clinical use limits the specificity and thus the quality of neuronal signals to be recorded. This limitation, in turn, hampers the precision of electrical stimulation, which may compromise the efficacy of neuromodulation therapies. Potentially going down to communication with individual neurons would facilitate alternative ways of implementing neuromodulation therapies. While such approaches remain out of reach of clinical practice, there are strategies of how to achieve this capability in the future.

To improve specificity and bidirectional coupling between a neuronal probe and neuronal tissue, the dimensions of the device should ideally be reduced to the micrometer scale. Such reductions in the dimensionality of neuronal probes have to be corroborated by maintaining the structural stability of the materials directly interfacing with the tissue, for the purpose of both safety and functionality. By reducing the electrode size to this scale, neuronal recordings could be captured at cellular spatial resolution, which would improve neuronal decoding. However, with microscopic electrode sizes, the electrode exhibits high impedances, limiting signal fidelity due to thermal noise and filtering.

To address the inherent neuronal interfacing issue and potential safety concerns associated with large metallic electrodes, high-performance materials have been explored to miniaturize neuronal interfaces. Due to its combination of several excellent material properties, graphene has emerged as an attractive candidate for the fabrication of micrometer-scale miniaturized, chronic, bidirectional neuronal interfaces. Graphene is one of the strongest, highly electrically conductive, and stable materials known, offering mainly capacitive interaction in aqueous media over a wide electrochemical potential window, while remaining mechanically flexible.^273^

**Figure 13:**
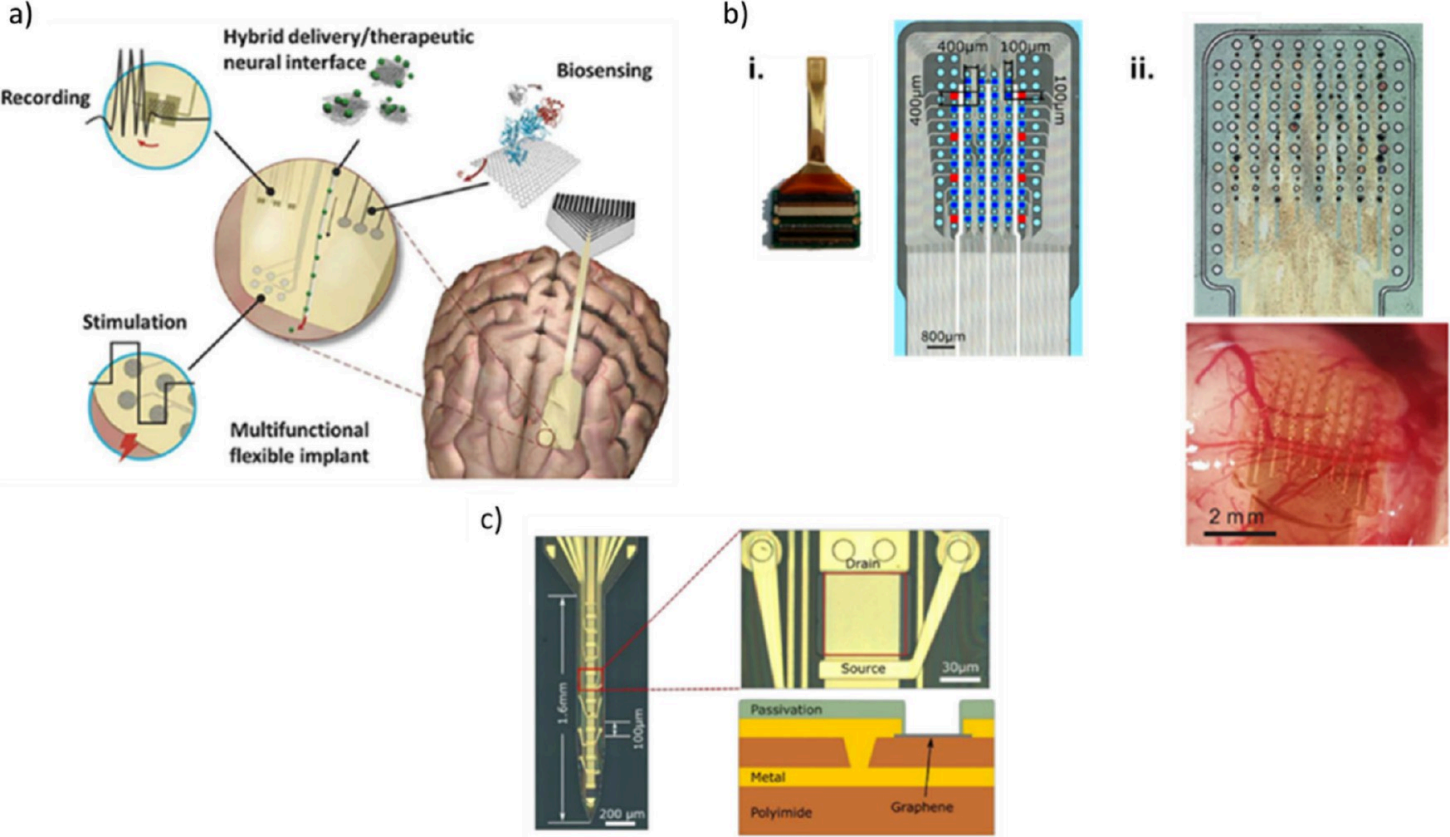
Graphene-based neuronal interfaces have been designed, fabricated, and quality-controlled to achieve reproducible functionality for brain signal recording, electrical neuronal stimulation, and biosensing. (a) Multifunctional graphene-based neuronal interface concept schematics. Modified with permission from Kostarelos *et a*l.^270^ Copyright 2017 Wiley-VCH. (b) Examples of functional graphene-based neuronal surface probes fabricated using (i) chemical vapor deposition (CVD) graphene field-effect transistor technology^278^ and (ii) reduced graphene oxide membrane technology used on a murine cortex (bottom image).^277^ Reproduced in modified form with permission from Garcia-Cortadella *et al*.^278^ and Viana *et al*.^277^ Copyright 2021 Springer Nature and 2022 The Authors, respectively. (c) Example of a graphene-based intracortical probe using the graphene field-effect transistor technology.^275^ Reprinted with permission from Bonaccini Calia *et al*.^275^ Copyright 2022 Springer Nature.

Recently, a generation of neuronal interface probes has been developed and fabricated (Figure 13) using wafer-scale processes routinely used in semiconductor cleanroom technology based on two types of graphene materials: CVD single-layer graphene sheets and reduced graphene oxide (rGO) porous membranes. The neuronal interface technology based on CVD single-layer graphene enables the preparation of field-effect transistors with the capacity to record electrical signals at a range of frequencies not previously possible.^274,275^ Furthermore, the transistor technology enables the design of flexible arrays of multiplexed sensors for high-density neuronal interfaces.^276^ Alternatively, the porous membranes based on rGO aim to design thin-film electrodes with enhanced ability for high charge injection and stability, for spatially focal neuronal stimulation and recording^277^ that could be translated to the clinic. In a series of studies, the graphene-based neuronal interface devices have been interrogated acutely *in vivo* to assess their performance for bidirectional neuronal interfacing using a variety of preclinical models, demonstrating miniaturization on a flexible substrate combined with material properties suitable for chronic implantation, inducing no significant nerve damage nor neuroinflammatory responses.

### Hydrogel-Based Interfaces

#### Conductive Polymers as Soft Interfaces

While the materials discussed above (solid-state electrodes, nanoparticles) are typically mechanically stiff materials, soft neuronal interfaces based on conductive polymers (CPs) can be made. One of the key advantages of CPs in neuronal interface applications is their ability to be easily tuned and optimized to achieve desired characteristics.^279^ This versatility allows for the design and development of interfaces tailored to specific applications and requirements. Furthermore, these materials enable the direct transmission of electrical, electrochemical, and electromechanical signals across the interface between living soft systems and abiotic electronic devices.^280^ In particular, the utilization of intrinsic CPs allows for appropriate conductivity and biocompatibility.

Conjugated polymers are a class of organic macromolecules characterized by their extended π-electron systems.^281^ This molecular arrangement, featuring alternating single and multiple bonds along the polymer backbone, facilitates the dispersion of electrons, resulting in the formation of stable and enduring charge-separated states when undergoing oxidation or reduction. These charged states exhibit quasi-particle behavior, enabling them to migrate and diffuse throughout the material. In contrast to metals, where electrons can occupy any position within the conduction band, charge transport in conjugated polymers occurs through a hopping mechanism between localized Wannier states.^282^ The concept of “doping,″ originating from the semiconductor field, pertains to the generation of charge carriers in conjugated polymers. This process involves the introduction of additional electrons (reduction or *n*-type doping) or the removal of electrons, leading to the formation of “holes” (oxidation or *p*-type doping). It is well-known that the electrochemical impedance of noble metals can be reduced by conjugated polymers.^283^ This reduction in impedance is primarily attributed to the increased uptake of ions across these polymers, especially when they are hydrophilic in nature, leading to an enlargement of the capacitance between the electrode and the electrolyte. Of note, it has been observed that the capacitance of the conjugated polymers coating exceeds that of bare gold for a given electrode size at lower frequencies, while the impedances become comparable at higher frequencies. This phenomenon underscores the potential of conjugated polymers in enhancing the electrical performance of neuronal interfaces, as their exceptional electrical conductivity make them highly attractive for integration into such applications.

Conducting polymers have electrical properties akin to semiconductors and metals, while maintaining mechanical properties similar to conventional polymers. Moreover, their electrochemical responsiveness allows for reversible changes in conductivity, color, wettability, and volume. This combination of hybrid electronic-ionic conductivity and biocompatibility suggests CPs as candidates for neuronal interface applications. To enhance their performance and compatibility with neuronal tissues, CPs are typically incorporated into hydrogel matrices.

Conductive neuronal interfaces based on hydrogel materials offer significant advances by harnessing the properties of both hydrogels and CPs. Hydrogels, which are three-dimensional networks of natural or synthetic polymers capable of absorbing and retaining large amounts of water or biological fluids, offer high biocompatibility and can be tailored to possess desirable properties for neuronal interface applications.^284^ By combining CPs with hydrogels, a soft and compliant interface can be created that not only retains the electrical conductivity of the CPs but also closely mimics the mechanical properties of native brain tissue. This integration of CPs into hydrogel matrices addresses the challenge of minimizing mechanical mismatches and reducing the foreign body response, scarring, and neuroinflammation commonly associated with traditional neuronal interfaces.^285^ Hydrogels provide a biocompatible and aqueous environment that promotes cell adhesion and supports the growth of neuronal tissue, while the CPs contribute to their electrical properties, allowing for efficient signal transmission between the interface and neuronal cells. The resulting conductive hydrogel-based neuronal interfaces offer enhanced biocompatibility, mechanical flexibility, and electrical conductivity, moving toward more seamless integration with the neuronal environment and improved long-term performance.^285^

#### Nanocomposite Hydrogels in Neuronal Interfaces

Nanocomposite hydrogels consist of 3D polymeric networks that are loaded with NPs, creating a water-swollen matrix capable of mimicking the hydration and biomimetic microenvironment found in native tissues.^286^ The fabrication of nanocomposite hydrogels with mechanical stability and bioactivity remains a persistent challenge; however, significant progress has been made in formulating hydrogels that can mimic the structure and physical properties of native tissues using natural and synthetic polymers.^287^ Nanocomposite hydrogels can incorporate various types of NPs that contribute particular properties to neuronal interfaces. Carbonaceous nanomaterials like CNTs and graphene exhibit excellent electrical conductivity, enabling seamless signal transmission between electronic devices and neuronal systems.^288^ Metallic NPs (*e.g.*, gold and silver) and metal oxide NPs offer not only electrical conductivity but also magnetic properties and photoresponsiveness, enabling controlled manipulation of neuronal activity and ensuring long-term stability.^289−291^ Organic NPs, such as polymeric NPs,^291^ can act as carriers for active agents, enabling targeted delivery for modulating cellular behavior.^292^ The physical and/or covalent interactions between these NPs and the polymeric backbone of the hydrogel result in a combinations of their properties.

One of the key motivations for utilizing nanocomposite hydrogels in neuronal interfaces is their ability to address the mechanical properties mismatch between the electrode and the brain tissue.^293^ The brain is a soft and delicate organ, while traditional electrode materials used in neuronal interfaces, such as metals, exhibit significantly different mechanical properties. This mismatch can lead to mechanical stress, tissue damage, and the activation of the immune response, ultimately compromising the long-term performance and biocompatibility of neuronal interfaces. However, the incorporation of nanomaterials into hydrogels for neuronal interfaces also poses challenges and concerns. Nanomaterials, especially CNTs and graphene, possess particular physical and chemical properties that can interact with biological systems in unpredictable ways, as discussed above.^294,295^ Their large surface areas and potential for cellular uptake raise concerns regarding cytotoxicity, inflammation, and long-term biocompatibility. The direct contact of nanomaterials embedded in hydrogel with human tissue introduces potential risks that need to be addressed through comprehensive biocompatibility assessments and rigorous safety testing.

**Figure 14:**
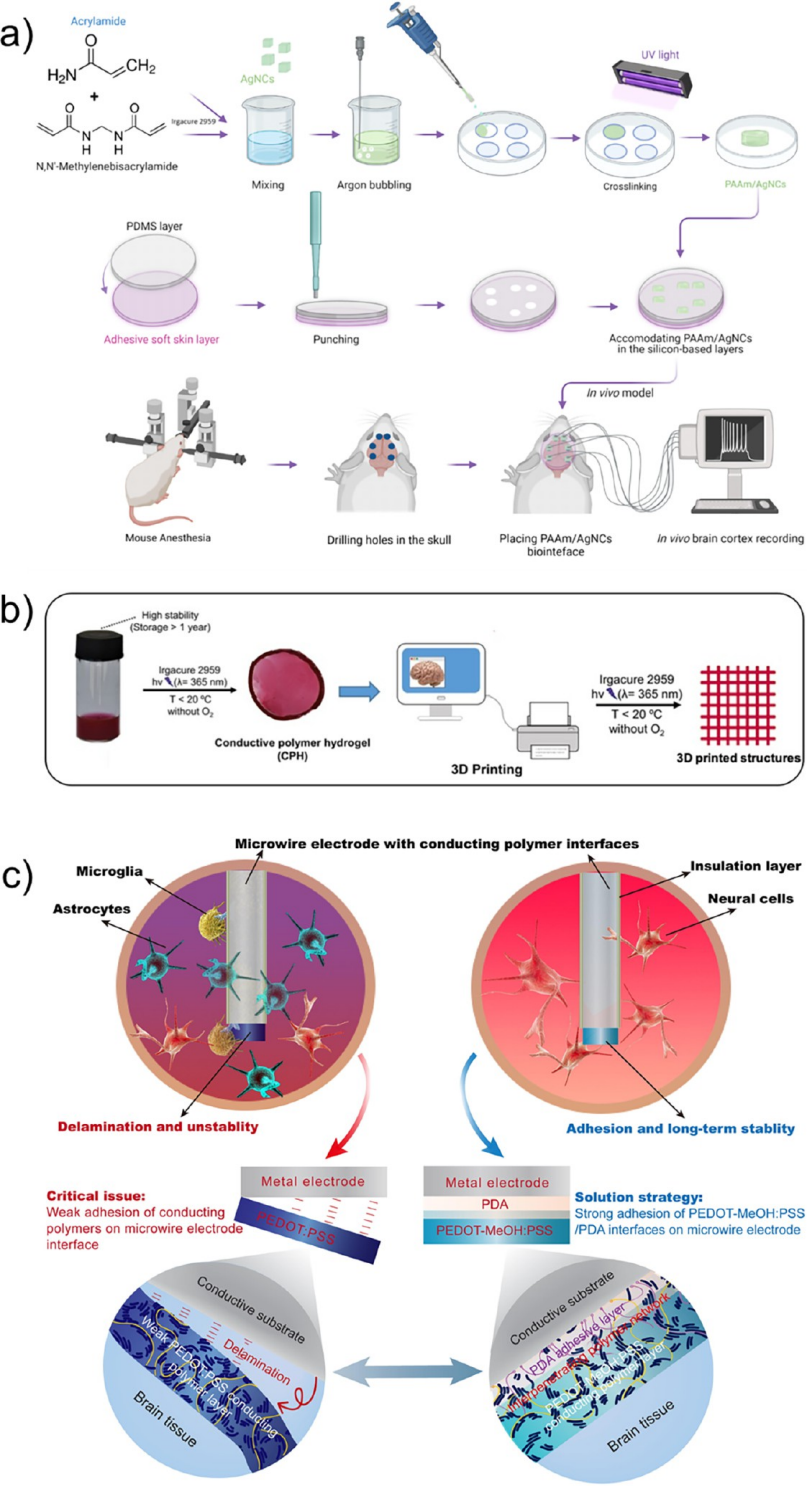
Conductive hydrogel-based neuronal interfaces: from molecular structures to applications. (a) A nanocomposite hydrogel composed of polyacrylamide and plasmonic silver nanocubes. The constructs benefit from well-dispersed silver nanocubes inside the hydrogel network, contributing to the formation of conducting pathways. The nanocomposite hydrogel was surrounded by a silicon-based template and utilized as a neuronal interface for *in vivo* electrocorticography (ECoG) recordings on a mouse model, and the long-term neuronal signal acquisition was practiced. Reproduced with permission from Rinoldi *et al*.^291^ Copyright 2022 American Chemical Society. (b) A conductive semi-interpenetrating network (IPN) hydrogel based on polythiophene. The hydrogel was synthesized by blending polythiophene with a poly(*N*-isopropylacrylamide) [p(NIPAAm)] copolymer, along with a cross-linker and photoinitiator. Subsequently, UV light exposure in a controlled, cold environment facilitated the formation of a conductive semi-IPN hydrogel to offer enhanced electrical conductivity, thermoresponsiveness, and biocompatibility. Reproduced with permission from Tian *et al*.^313^ Copyright 2021 American Chemical Society. (c) A conductive IPN hydrogel based on poly(3,4-ethylenedioxythiophene)-MeOH:poly(styrenesulfonate)/polydopamine (PEDOT-MeOH:PSS/PDA). The design of this adhesive conducting interface involves the incorporation of a thin PDA layer to enable the formation of interpenetrating networks through electropolymerization. The fabrication procedure follows a simple two-step methodology. Initially, PDA is electropolymerized to create an adhesive conductive thin layer on the wire microelectrodes. Subsequently, EDOT-MeOH with PSS acting as the supported polyelectrolyte undergoes electropolymerization to generate the desired interpenetrating PEDOT-MeOH:PSS/PDA networks. Reproduced with permission from Tian *et al*.^313^ Copyright 2023 Elsevier Inc.

To overcome these challenges, researchers are actively exploring a number of strategies. One approach involves surface modifications of the nanomaterials to enhance their biocompatibility and to minimize adverse interactions with biological systems,^295^ discussed below in greater detail. Encapsulation techniques can be employed to create a protective barrier around the nanomaterials, preventing direct contact with the surrounding tissue. Ensuring the nanocomposite hydrogels’ stability and long-term performance is crucial for maintaining their desired properties over extended periods of use. In recent discoveries, the incorporation of a stabilizing and protecting agent, such as polyvinylpyrrolidone (PVP), has been found advantageous in minimizing silver NP aggregation and controlling the reduction rate of silver ions.^291^ Additionally, the presence of PVP moieties has been observed to ensure dispersion of silver nanocubes in aqueous environment. This stable and well-dispersed precursor solution can then be utilized for the preparation of polyacrylamide/silver nanocubes hydrogels (see Figure 14a). The resulting nanosilver-laden nanocomposite hydrogel exhibits useful electrical properties, biocompatibility, and demonstrates no adverse effects during *in vivo* evaluation. On the other hand, the incorporation of a combination of different well-dispersed NPs within a hydrogel matrix for neuronal interfaces offers significant advantages by harnessing the particular properties of each type of NPs.^296−298^ By synergistically combining NPs with diverse functionalities, such as metallic NPs for electrical conductivity and magnetic properties, and polymeric NPs for targeted delivery, the resulting nanocomposite hydrogel can exhibit a comprehensive range of properties necessary for optimized neuronal interface performance. This multifunctional approach enables the design of advanced bio interfaces that can potentially integrate with neuronal systems.

To enhance the precision and scalability of nanocomposite hydrogel production, fabrication methods such as two-photon polymerization (TPP), 3D bioprinting, electrospinning, and self-assembly approaches are being developed.^290,299^ A recent example of 3D bioprinting represents the fabrication of a conductive hydrogel with projection microstereolithography.^300^ The techniques involve a top-down projection microstereolithography system with an UV-LED light source (405 nm) and a digital mirror device. A combination of these methods has also been investigated. For instance, by combining two-photon hydrogelation (TPH) and *in situ* self-assembly of poly(3,4-ethylenedioxythiophene) (PEDOT), a conductive nanocomposite hydrogel was fabricated at a microscopic scale having multiwalled CNTs as its conductive counterpart.^301^ The TPH can be regarded as a step up of TPP process to create photon-curable materials. By employing these two fabrication techniques, the level of precision in lateral feature sizes was reduced to 200 nm, surpassing the limitations imposed by optical diffraction. These techniques allow for the creation of complex structures with precise control over the spatial arrangement of nanomaterials within the hydrogel matrix. By leveraging these advances in fabrication methods, researchers are developing intricate neuronal interfaces with tailored properties, promoting seamless integration between the interface and neuronal tissue. In parallel, researchers are exploring innovative methods for applying nanocomposite hydrogels in neuronal interfaces to optimize their performance and functionality. Surface patterning^302^ and biofunctionalization^303^ are some of the strategies being investigated. Note that the incorporation of nanocomposite hydrogels on/into neuronal electrodes necessitates the utilization of sophisticated patterning techniques to fulfill the miniaturization demands for achieving high spatial resolution and long-term adhesion, ensuring sustained performance over extended periods. Conventional methods for patterning include dip coating, electrodeposition, inkjet printing, or multistep microfabrication processes.^304^ The incorporation of various bioactive molecules or growth factors has been reported to enhance the performance of the device. Examples include L1, laminin, and nerve growth factor (NGF) for improved functionality, as well as melanocyte-stimulating hormone (MSH) to reduce the anti-inflammatory response.^296^ For instance, MSH coating has shown potential in reducing glial fibrillary acidic protein (GFAP) staining of astrocytes and ED-1 staining of activated microglia and *in vivo*.^305^ These approaches aim to promote specific cellular interactions, facilitate tissue regeneration, and enhance signal transmission, creating a bioactive and responsive interface that closely mimics the complexity of the neuronal environment.

#### Semi-Interpenetrating Networks (IPNs) and IPNs Based on Conjugated Polymers

Within the context of incorporating CPs into hydrogels, semi-interpenetrating network (IPN) and IPN systems emerge as a means to unlock their full potential. In the case of semi-IPN structures, the conjugated polymer chains infiltrate a polymeric hydrogel network at the molecular level, creating a state of physical entanglement devoid of covalent cross-links.^306,307^ Consequently, the linear or branched conjugated polymers in the conductive semi-IPN hydrogel can, in principle, be detached from the polymeric network(s) without disrupting chemical bonds, essentially establishing it as a polymer blend.^308^ This distinctive arrangement facilitates the synergistic integration of the different properties inherent in both components, leading to an elevation in the overall conductivity and mechanical strength of the hydrogel. Conversely, in the structure of a conductive IPN hydrogel, the network of a conjugated polymer intricately interweaves, on molecular scale, with a polymeric hydrogel network.^306^ Additionally, an IPN exhibits limited phase separation, where its networks remain inseparable until chemical bonds are severed, and at least one of its components is synthesized or cross-linked in the immediate presence of the other.^309^ This interlocking of polymer chains within the IPN engenders a more intimately intertwined network. Consequently, IPN hydrogels demonstrate enhanced mechanical stability and structural integrity when compared to a single-network hydrogel, owing to the permanent interpenetration and interlocking of network segments.

There are several advantages of conductive hydrogels based on conjugated polymers, such as polyaniline,^310,311^ polypyrrole,^312^ polythiophene,^313^ and PEDOT^309,314^ over metallic or carbonaceous alternatives. Metal-based hydrogels, utilizing materials such as silver NPs, suffer from toxicity concerns due to the release of metal ions. These ions can potentially induce adverse biological reactions and cellular damage in the surrounding neuronal tissue. Furthermore, the stability of metallic-based hydrogels may be compromised over time, leading to degradation of the electrical properties and reduced performance as the metal nanoparticles agglomerate or undergo corrosion. Carbonaceous materials, such as CNTs and graphene, have shown promise for enhancing the electrical conductivity of hydrogels. However, they present challenges that hinder their widespread use in neuronal interfaces.^315^ One of the main concerns is their potential toxicity. Both, CNTs and graphene sheets have high aspect ratios, which can lead to adverse biological effects when they come into contact with living cells or tissues. The sharp edges and high surface areas of these materials can cause mechanical damage to cells and induce inflammatory responses.^316^ The hydrophobic nature of CNTs and graphene makes it challenging to disperse them uniformly within the hydrogel matrix, resulting in aggregation and poor integration with the surrounding environment. Conjugated polymers, on the other hand, offer high biocompatibility and stability within the hydrogel matrix, making them more suitable for long-term implantation in neuronal interfaces. These polymers can interact with biological molecules and cells, promoting the formation of neuronal networks and facilitating the transmission of electrical signals. Polythiophene, for instance, is a widely studied conjugated polymer due to its favorable properties, including high electrical conductivity, stability, and biocompatibility.^317^ Polythiophene-based hydrogels can be easily synthesized and processed into various shapes and forms, making them versatile for different neuronal interface applications. For instance, a polythiophene derivative, poly[3–14(potassium-5-butanoate)thiophene-2,5-diyl] (P3KBT), was incorporated in a nanostructured PVA hydrogel.^318^ Cross-linking conditions are thought to play important roles in determining the conductivity of semi-IPN hydrogels. Specifically, these conditions influence the formation of conducting networks that facilitate charge transport. Elsewhere, another type of polythiophene, poly[6-(3-thienyl)-hexanesulfonate] (P3HT6S), was employed as the conjugated polymer component for poly(*N*-isopropylacrylamide) (p(NIPAAm)) copolymer hydrogel neuronal interface (Figure 14b).^308^ The lower electrical impedance of the conductive semi-IPN was coupled with markedly improved mechanical properties compared to the p(NIPAAm)-copolymer hydrogel. The semi-IPN hydrogel based on P3HT6S exhibited an average Young’s modulus of approximately 4.6 ± 0.3 kPa, demonstrating mechanical properties similar to those of brain slices (1–10 kPa). In addition to its favorable mechanical characteristics, the semi-IPN hydrogel yields high cell viability rates when cells were directly cultured on the hydrogel. Furthermore, the neuronal differentiation percentage was approximately 20% for the P(NIPAAm)/P3HT6S semi-IPN hydrogel, surpassing the 15% observed for bare hydrogel.

To fabricate conductive semi-IPN and IPN hydrogels based on conjugated polymers, various methods have been explored to incorporate the CPs’ chains into the hydrogel network.^293,319^*In situ* polymerization is one approach: monomers of the conjugated polymers are polymerized within the hydrogel matrix, ensuring a uniform distribution and interpenetration of the polymer chains. Physical blending involves mixing presynthesized conjugated polymers with hydrogel precursors, followed by gelation. Chemical cross-linking utilizes cross-linkers that can simultaneously or stepwise cross-link the hydrogel and conjugated polymer chains, forming cohesive networks.^309^ In parallel, other advanced fabrication methods have expanded the possibilities for creating innovative conducting semi-IPN and IPN hydrogels. Techniques such as electrospinning and additive manufacturing have been explored to achieve high-resolution and miniaturized devices with improved spatial resolution.^302,320^ These methods offer precise control over the structure and properties of the interfaces, enabling the design of tailored interfaces for specific applications.

Despite the immense potential of conductive semi-IPN and IPN hydrogels based on conjugated polymers, several challenges remain to be addressed. One significant challenge is the trade-off between the water uptake/swelling of a hydrogel and the improved electrical properties when the conjugated polymer is present in the hydrogel network, specifically when the construct is designed as a neuronal interface. In one study, the incorporation of polyaniline in a semi-IPN chitosan-agarose hydrogel increased the conductivity to approximately 10^–4^ S/cm (which falls within the range suitable for neuronal tissue engineering applications), but the hydrophobic polyaniline reduced the swelling rate of the hydrogel, resulting in decreased swelling capacity from approximately 800% for the pristine hydrogel to *ca*. 300% for the conductive hydrogel.^321^ While substantial progress has been made in enhancing the mechanical and electrochemical stability of semi-IPN or IPN hydrogels, there remain challenges in achieving reliable adhesion between these flexible structures and the underlying neuronal stimulation/recording substrate. The objective is to prevent delamination of the soft coating, which could disrupt the electrical connection and impair the functionality of the device.^322^ It is anticipated that low adhesion would occur between the rigid surface of a neuronal electrode and a fully swelled hydrogel; therefore, it is crucial for these soft coatings to possess the capability to be patterned and selectively deposited onto electrode sites before the formation of the hydrogel network. One potential approach involves a three-step process: first, the functionalization or modification of the neuronal stimulation/recording substrate, which can involve chemical modifications to introduce functional groups or physical manipulations to enhance surface roughness. The second step involves the deposition or addition of the hydrogel precursor onto the modified substrate, followed by the formation of the conductive semi-IPN or IPN.

Since gold and PDMS are commonly used materials in neuronal probes, their chemical modification has garnered considerable attention. An example of such modifications involves the introduction of acryloyl groups to gold through the reaction with *N*,*N′*-bis(acryloyl)cystamine (BAC). Similarly, PDMS surfaces have been modified using silanization agents like 3-(trimethoxysilyl)propyl methacrylate (TMSPMA). This particular approach proves particularly advantageous for developing conductive semi-IPN or IPN coatings based on P(NIPAAm), polyacrylamide, and poly(dimethylacrylamide) copolymers, such as poly(DMAA-*co*-5%MABP-*co*-2,5%SSNa) (PDMAAp) hydrogels.^322^ The interaction between the functional groups of these polymeric chains and the surface groups formed on the neuronal electrodes, such as amine acrylate reactions, contributes to the enhanced stability of the hydrogel interface. In a relevant study, the gold substrate of the neuronal probe underwent chemical modification using cysteamine.^323^ Subsequently, the hydrogel precursor, gelatin methacrylate (GelMa), was electrodeposited onto the modified substrate, followed by the electropolymerization of a polypyrrole conjugated polymer within the hydrogel network. The semi-IPN of GelMa/polypyrrole showed stability and resilience after 1000 cyclic voltammetry (CV) cycles. An interesting finding in this study was that the polypyrrole coating, in the absence of the hydrogel support, displayed noticeable signs of delamination during CV tests, pointing toward the weak adhesion between the conjugated polymer and the gold electrode.

Three significant challenges persist in the development of stable and reliable conductive semi-IPN or IPN hydrogel neuronal interfaces. First, the integration of these soft layers is intricate and costly. Recent advancements have sought to address this challenge by introducing a neuronal interface integrated into ultrasmall microelectrodes through a simple two-step electropolymerization strategy.^313^ This strategy entails the initial prepolymerization of a thin adhesive layer of polydopamine (PDA) to enhance the interfacial adhesion of the conducting polymer film without compromising its electrical conductivity. Subsequently, the electropolymerization of EDOT-MeOH with poly(styrenesulfonate) (PSS) is performed to generate interpenetrating PEDOT-MeOH:PSS/PDA networks, further enhancing the functionality and performance of the neuronal interface (Figure 14c). The obtained conductive IPN hydrogel demonstrated good interfacial adhesion, even when subjected to continuous sonication at 500 W for 20 min. Additionally, the integration of the PEDOT-MeOH:PSS/PDA interface on ultrasmall Pt–Ir wire microelectrodes with a diameter as low as 10 μm has yielded improved results. The modified interface exhibits significantly lower impedance at 1000 Hz and long-term stability, enduring up to 10,000,000 biphasic input pulses. Second, the *in situ* growth efficiency of the conjugated polymer is relatively low. Addressing this challenge involves the consideration of the density of propagation sites and interdopant spacing.^324^ Recent advances have revealed a direct correlation between interdopant spacing and impedance. A controlled method has been developed to integrate dopants into the polymer backbone through chemical modification of the PVA macromers with sulfonic acid, enabling precise control over interdopant spacing and dopant density.^325^ The developed method enables the manipulation of interdopant spacing and dopant density, allowing for variability and control in these parameters. Third, the presence of the hydrogel component in conductive semi-IPN and IPN interfaces can have a detrimental effect on the electrical properties. For example, when gold neuronal electrodes were coated with GelMa hydrogels, the observed charge storage capacity (CSC) decreased from 0.61 ± 0.07 mC cm^–2^ to 0.31 ± 0.01 mC cm^–2^. This reduction in CSC was attributed to factors such as the molecular weight of GelMa and the thickness of the hydrogel coating. However, by incorporating polypyrrole growth within GelMa through electrochemical polymerization and forming a semi-IPN structure, the CSC increased to 3.47 ± 0.12 mC cm^–2^. Nonetheless, there remains a need to address the miniaturization of the hydrogel layer and to gain control over the molecular weight of the hydrogel polymeric backbone to optimize the interface further.

Building upon the aforementioned challenge of achieving reliable adhesion between the soft layer and the underlying rigid substrate, a promising approach for achieving stable chronic neuromodulation is to establish a robust neuronal interface that ensures close electrical coupling with neuronal tissue. The concept involves utilizing a conductive semi-IPN or IPN as a bioadhesive hydrogel while maintaining adequate adhesion to the electrode surface. This necessitates the development of a specialized type of neuronal interface capable of securely affixing the neuronal electrodes onto the surface of the brain tissue. Recently, a hydrogel has been developed to address these specific requirements.^326^ In this study, a conductive (∼6 S·cm^–1^) and bioadhesive (interfacial toughness ∼100 J·m^–2^) semi-IPN hydrogel based on polyethylene glycol diacrylate (PEGDA) and PEDOT:PSS conjugated polymer was constructed. A key component of the design is the incorporation of a UV-sensitive zwitterionic monomer, [2-(methacryloyloxy)ethyl]dimethyl-(3-sulfopropyl) (SBMA). This approach is particularly interesting as zwitterionic polymers can interact with the polar groups of the modified neuronal probe surface, promoting adhesion to the hydrogel construct. Furthermore, zwitterionic polymers possess both positive and negative charges, as well as good ion-conductive properties, making them a good choice for blending with conjugated polymers.^327^ This blending strategy allows for tailoring electrical properties in semi-IPN or IPN hydrogels, specifically for applications in neuronal interfaces.

Matching mechanical properties is also a challenge for hydrogels in neuronal interfaces. Neuronal tissue is inherently soft and delicate, requiring hydrogels to mimic its mechanical behavior closely to minimize mechanical mismatch and potential trauma. While it is crucial to achieve mechanical similarity between the hydrogel and the brain, the delicacy of the hydrogel introduces a risk to its integrity during handling and application, as it may be susceptible to cracking or disintegration. To address this challenge, incorporation of self-healing properties into the hydrogel has been practiced. Self-healing hydrogels possess the ability to repair damage and/or defects autonomously, restoring their structural integrity. This process is achieved by introducing dynamic chemical bonds or reversible physical interactions within the hydrogel network, which can be activated when the material is damaged. The self-healing capability adds additional safeguards against cracking or disintegration, significantly enhancing the durability and reliability of the hydrogel during handling and application.

Unlocking alternative fabrication methods relies on the presence of specific properties conferred upon the material, as well as their combination and arrangement. For instance, the formulation of P(NIPAAm)/P3HT6S as an ink for 3D printing takes advantage of a straightforward cross-linking method.^308^ This mixture is highly suitable for 3D custom printing applications. Similarly, the semi-IPN hydrogel of PEDOT:PSS/zwitterionic SBMA offers the advantage of being photopatternable.^326^ This advance enables facile microfabrication processes, facilitating the creation of multifunctional interfaces with characteristic sizes as small as 50 μm. Neuronal electrodes based on this hydrogel demonstrate improved performance in terms of impedance, charge storage capacity, and charge injection. Moreover, they exhibit the ability to deliver effective electrical stimulation with high current density (1 mA·cm^–2^) at low voltages (±25 mV). Ongoing research is dedicated to exploring avenues for improving the electrical conductivity, stability, and biocompatibility of semi-IPN and IPN hydrogels embedded with conjugated polymers. Scientists are actively developing conjugated polymers with precisely controlled molecular structures and customized side chains to enhance their charge transport properties and facilitate seamless neuronal interfacing.^328^ Additionally, there is growing interest in the development of smart hydrogels with multimodal responsiveness, including pH, temperature, and/or light sensitivity.^329,330^ In particular, the development of shape-memory and thermoresponsive materials holds great potential for enhancing the functionality and usability of neuronal interfaces. Shape-memory materials can provide ease of handling during the implantation procedure, while thermoresponsive materials enable wireless neuronal transduction, eliminating the need for invasive wiring and external energy sources.^293^ These advanced smart hydrogels exhibit features important for applications, such as improved stretchability, rapid self-healing, excellent tissue adhesion, and the ability to respond to multiple stimuli. They offer the potential for on-demand drug release or electrical stimulation, granting precise control over neuronal interactions and expanding the range of functionalities in neuronal interfaces. These advances are important for the next generation of conductive semi-IPN and IPN hydrogels characterized by enhanced conductivity, conformal biointegration features, and multistimuli-responsivity.

### Improving Biocompatibility *via* Surface Coatings

As discussed above for hydrogels, the mechanical mismatch between conventional stiff electrodes (with Young’s modulus of some GPa) and soft neuronal tissue (with Young’s moduli of some kPa^78^) can lead to immunological response and cell damage in the brain. On the other hand, the activation of microglia cells and the formation of an astroglial sheath on the surface of electrical implants undermines the function of implanted electrodes^331^ and may cause neuroinflammation.^332−335^ Hence, appropriate and structural biocompatibility is a prerequisite for successful medical implantation of electrodes in the brain. Many physicochemical parameters affect interfacing a nanometer-sized electrode with brain tissue,^81,336^ such as electrode materials, electrode structures, and surface coatings.^337,338^ For modulating the implant-host tissue reaction, appropriate surface chemistry is essential for creating long-term stable and biocompatible electrodes in the brain. Proper coatings can render a nonbiocompatible electrode surface^339^ biocompatible.^340^ Neurons, in general, are sensitive to their environment and their electrophysiological function can be impaired by underlying electrode surfaces.^341^ The coatings used are thus essential.

Apart from hydrogel coatings, by simply modifying electrode surfaces chemically with polymers, neuronal growth and adherence can be improved. Guiseppi-Elie *et al*. found that cysteamine (CA) self-assembled monolayers exhibit better performance than those of ω-amine alkanethiols (*e.g.*, 11-amino-1-undecanethiol, 11-AUT) on the adherence of neurons to gold electrodes by modulating surface roughness, functional groups, and adhesion proteins.^342^ In order to lower interface impedance and improve neuronal interactions, some conductive polymers, such as PEDOT, have been frequently used to modify implantable electrodes,^343^ attracting increasing interest.^344−346^ Moreover, carbon-based nanomaterials such as CNTs, graphene, and “glassy diamond″^347,348^ have also been applied for the surface coating of neuronal electrodes to improve the stability, biocompatibility, and electromagnetic properties of electrodes,^349,350^ as discussed in the above sections.

In addition to surface coatings, another option is to deliver anti-inflammatory drugs to the brain to mitigate the brain microenvironment and to suppress the immunological response induced by implanted electrodes. For example, Cui *et al*. showed that a polypyrrole-coating of the electrode can lower the activation of astrocytes *in vitro* through the electrically controlled release of dexamethasone (Dex) as an anti-inflammatory drug.^351^ In this scenario, although substantial progress has been made for better brain–machine interfaces in recent years, the long-term stable and biocompatible incorporation of nanostructured electrodes in the brain remains a major challenge.^49,352^ Patterned coatings also might help to control which types of cells adheres to electrodes.^353^

### Roles of Non-neuronal Cells

Roughly half the cells in the human brain are not neurons. The most recent count indicates *ca*. 86 billion neurons vs. *ca*. 85 billion glia cells. Glial cells, microglia and macroglia, and also pericytes and endothelial cells occupy important fractions of brain cells. The fraction of glial cells increases with phylogeny. Among the macroglia, astrocytes form an abundant, nonaction-potential firing but excitable cell population. Although expressing ion channels as well as electrogenic transporters, astrocytes are often considered “electrically silent”, which is probably more the result of our inability to voltage- or current-clamp the extensive and leaky arborization of these cells that are coupled through gap junctions to form a large syncytium. Despite significant progress in understanding the contributions of glial cells and particularly those of cortical astrocytes and Bergmann glia of the cerebellum to brain metabolism and signaling, our understanding of how glial cells plug into neuronal circuits and which higher brain functions are orchestrated by these cells remains limited.^354^ As voltage control is restricted, studying glia and glial heterogeneity instead requires optogenetic stimulation strategies.^355^ However, astrocytes readily take up different NPs^356−358^ and respond in various manners, a strategy that has been used, *e.g.*, for cell-type-specific gene therapy,^359^ targeted drug delivery to the neuro-vascular unit,^360^ or treating spinal-cord injury.^361^

## Colloidal Nanoparticles as Transducers for Communication with Different Ion Channels or Neurotransmitters

Neuronal signaling works electrically *via* ionic currents through ion channels traversing the cell membrane and ions spreading along the cell and chemically *via* neurotransmitters. These signals however are not necessarily the easiest ones for external readout and stimulation. Nanomaterials can be used as signal transducers, allowing for a larger variety of signal types for the external interaction with neurons. In this section, different transducer modalities are described. However, we note that while excellent NP-based transducers are available; the challenge to direct them *in vivo* to the desired location (*e.g.*, close to specific ion channels) remains largely unsolved.

### Different Gating Mechanisms of Ion Channels

As neurons express a plethora of receptors next to the classical voltage-gated ion channels and thus electrically stimulated ones, more activating methods are conceivable. While one can engineer ion channels that are gated by certain external stimuli, many different gating mechanisms are already naturally available.

Light is a natural trigger for biological functions such as phototaxis in plants, diurnal rhythms, and vision in higher animals. The pathway with the highest bandwidth to the brain is through the eyes. The 1967 Nobel Prize was awarded to Wald, Granit, and Harline for elucidating the molecular steps of the visual process.^362^ Rhodopsins, the key proteins for light sensing and information transfer to the brain, are a family of structurally closely related molecules in the eye. Rhodopsins are among the oldest proteins in evolution^363,364^ and are widespread in single-cell organisms up to the most complex. Early forms of an eye are found in bacteria, *e.g.*, bacteriorhodopsin in *Halobacterium halobium*,^365^ in algae, *e.g.*, channelrhodopsin in *Chlamydomonas*,^366^ and various rhodopsins in ∼95% of all animals. Even in organisms living entirely in the dark, rhodopsins have been found.^367^

**Figure 15:**
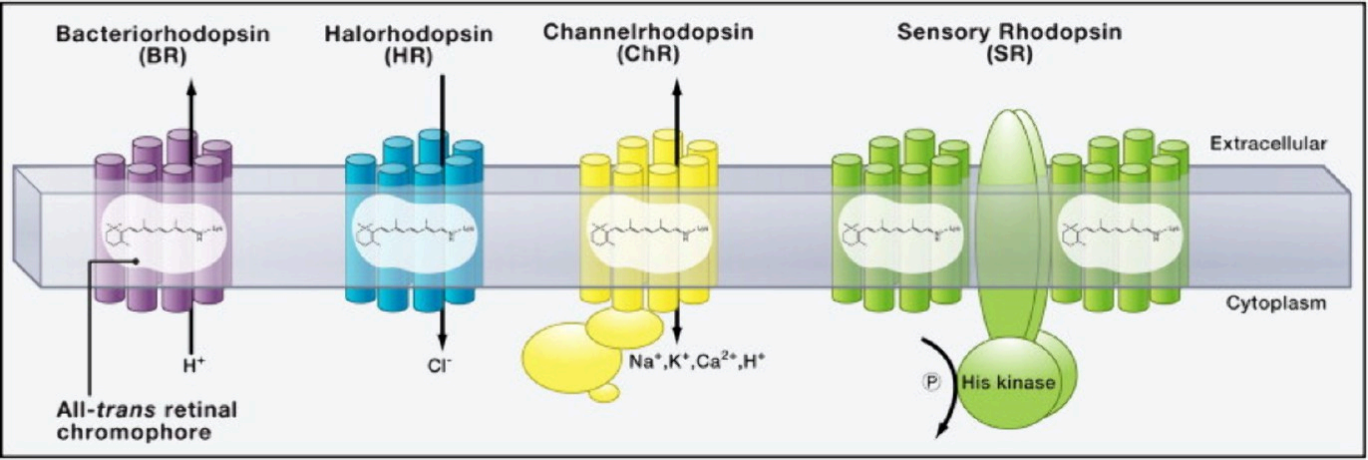
Different members from the rhodopsin family and their function. Image reproduced with permission from Zhang *et al*.^369^ Copyright 2011 Elsevier.

**Figure 16:**
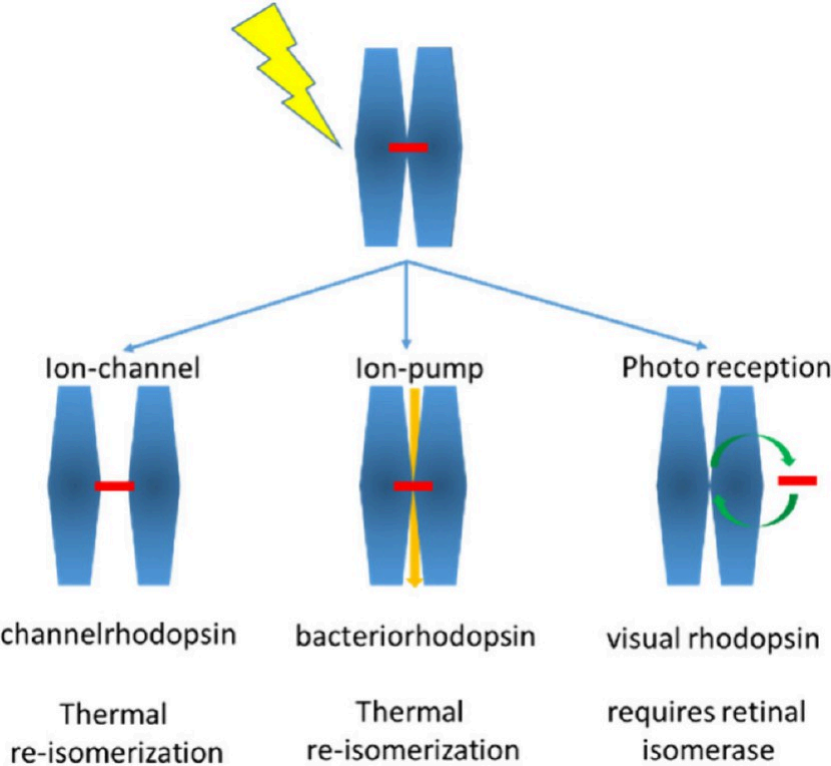
Three types of phototriggered rhodopsins: (left) light-triggered ion-channel thermally reverting to its initial state, *e.g.*, channelrhodopsins; (middle) light-triggered ion pump thermally reverting, *e.g.*, bacteriorhodopsin and halorhodopsin; (right) light-triggered sensory pigment requires retinal isomerase to revert to its initial state. For optogenetic use, channelrhodopsin is the best-suited molecule.^370^

Rhodopsin functions are highly variable.^368^ They can act as proton or chloride pumps, as gated ion channels, or as light-sensing molecules triggering cellular functions such as switching a flagellar motor resembling an early form of an eye, see Figure 15. The seven α-helical rhodopsin family is a versatile molecular platform to supply various light-triggered functions. All rhodopsins contain a retinal molecule that forms the chromophore, together with an inner shell of amino acids. Those amino acids also control the photochemical pathway, i.e., no random isomerizations at one or more of the double bonds occurs, as would be observed with rhodopsin in solution, but precisely only one at C9/C10, or C11/C12, or C13/C14. All rhodopsins effectively absorb light and transfer energy to the protein moiety. But, the various rhodopsins differ in one crucial aspect (Figure 16). Light triggers the isomerization of the bound retinal, but how does isomerization revert back to the initial configuration? Dissolved retinal would *not* reisomerize at ambient temperature. Numerous rhodopsins catalyze the thermal reisomerization to return the retinal to its initial conformation so that the cycle can restart. Some rhodopsin species act like an enzyme and reduce the energy barrier for reisomerization. Still other rhodopsins, *e.g.*, the rhodopsins in the human eye, release the retinal, which then diffuses to an external enzyme, a retinal isomerase, reisomerizes, and then diffuses back to the binding pocket of the rhodopsins. This process is the reason for the relatively limited temporal resolution of our visual system.

Among the rhodopsin family channelrhodopsins are particularly useful to interact with the brain by light. Channelrhodopsins are light-gated ion channels that thermally return to their initial state. The gene coding for channelrhodopsin can be inserted into practically any organism using common genetic tools. This combination of optical control and genetic tools^371^ opened the field of optogenetics, with channelrhodopsin as the enabling tool.^369^

Based on such light-sensitive proteins, optogenetics is a rapidly emerging field of research and applications.^368,372−374^ Optogenetics has strongly influenced basic research in neuroscience and cell biology and has enormous potential for medical applications such as vision or hearing restoration, the development of innovative therapies for diseases including cancer, diabetes, and neuronal disorders. Optogenetic tools utilize light-sensitive proteins, typically ion channels or pumps, that either activate or suppress neuronal signaling in response to specific wavelengths of light (neuronal photoactivation).^375−377^ Neurons can be controlled *via* fast and specific excitation or inhibition on a temporal scale commensurate with physiological conditions.^378−380^ Specific cells (or tissues, organs, or organisms) can be made light-sensitive by the heterologous expression of microbial sensory photoreceptors, thereby enabling the light-induced triggering of specific physiological responses^381^ in a reversible, spatiotemporally selective, and noninvasive manner. Recent advances in optogenetics focus on optimizing the light-gated ion channels themselves, the light delivery systems,^382^ simultaneous sensing of neuronal activity, and the optogene delivery system. Despite significant progress, considerable challenges for the widespread implementation of optogenetics are still related to the light source, *i.e.*, unsatisfactory penetration depth, or the need for invasive insertion of a light source related to the spectral sensitivity of existing light-gated channels. In addition, *in vivo* implementation of optogenetics in the clinic would require modification of the patient genome. Visible light (vis), particularly in the blue or green/yellow spectrum, is required for the photoactivation of channelrhodopsin-2 (ChR2) and halorhodopsin (HR).^383^ These wavelengths are strongly absorbed and scattered in biological tissues and, depending on the dose, can lead to phototoxicity. Due to low tissue penetration, invasive fiber-optic probes typically are required to deliver visible light deep into organs and animal tissues. This intrusion carries the risk of tissue damage and inflammation. Other challenges to optogenetics are related to the variation in channel expression, slow response, low signal/noise ratio, and/or low fluorescence of the ion/voltage-sensitive optical sensors and the lack of cell-type specificity of the optogene delivery system.

Channelrhodopsins are the most frequently used optogenetic tools to stimulate neurons, however, their activation poorly reflects astrocyte physiology. Although astrocytes express large numbers of ion channels, they mainly respond to synaptically released neurotransmitters *via* Gq protein-coupled receptors that mediate intracellular Ca^2+^ release.^384^ Most channelrhodopsins have rather low Ca^2+^ permeability and mainly conduct Na^+^, K^+^, and H^+^, leading to ion fluxes across the plasma membrane that naturally do not occur in astrocytes, at least not to the extent generated by channelrhodopsins. Light-activated G-protein-coupled receptors can be used as optogenetic tools that better reflect signaling mechanisms in astrocytes. Vertebrate rhodopsins are G-protein coupled and can be activated by visible light. However, transducin, the G protein coupled to vertebrate rhodopsin, links to phosphodiesterase activation and thus deviates from the Gq-mediated signaling cascade employed by astrocytic neurotransmitter receptors, rendering native rhodopsin unsuitable to evoke Ca^2+^ signaling in astrocytes.^385^ This problem can be circumvented by the generation of rhodopsin chimaeras, assembled from the rhodopsin core (consisting of extracellular loops and transmembrane helices) and the intracellular loops of a Gq-coupled receptor such as the α1-adrenergic receptor, resulting in a light-activatable Gq-coupled receptor (opto-α1AR).^386^ Opto-α1AR has been expressed in astrocytes and triggers Ca^2+^ signals upon illumination, using the same intracellular signaling cascade as the natural adrenergic receptor.^387,388^ Ca^2+^ is the main second messenger in astrocytes and is involved in virtually all astrocyte functions. Ca^2+^ signals in astrocytes trigger the release of transmitter molecules such as ATP, glutamate, d-serine, prostaglandins, and arachidonic acid that act on adjacent neurons, microglial cells and blood vessels.^384,389^ Consequently, Ca^2+^ signaling in astrocytes results in neurovascular coupling, neuronal excitation, synaptic plasticity, and finally shapes behavior.^390,391^ Ca^2+^ imaging is the method of choice to study astrocyte physiology.^392,393^ Genetically encoded Ca^2+^ indicators have replaced chemical Ca^2+^ indicators in recent years and allow for real-time visualization of astrocytic Ca^2+^ signals *in vivo* at high spatial and temporal resolution using two-photon excitation.^393^ Besides Ca^2+^, cyclic adenosine-monophosphate (cAMP) is an important second messenger and rhodopsin chimeras linking to Gs proteins have been assembled, resulting in light activation of cAMP production.^386,394^ In addition, bacterial light-activated adenylyl cyclases generating cAMP upon illumination and genetically encoded fluorescent cAMP indicators are optogenetic tools to study cAMP signaling.^395−398^ Using these tools, cAMP signaling in astrocytes has been shown to modulate synaptic plasticity and memory formation.^394,399,400^

Another important external trigger of biological reactions is heat.^401^ Examples are thermosensitive vanilloid transient potential receptors (TRPV), known in daily life for their agonistic substance, capsaicin, also found in hot chilies.^402−404^ The entire family of transient receptor potential (TRP) channels are rewarding targets, as some TRP channels have a thousand times higher conductivity than current channelrhodopsins.^405^ Heat can be supplied, for example, by photothermal or magnetothermal stimulation. Magnetothermal stimulation *via* magnetic NPs injected^403^ in different regions of mice brains leads to clear behavioral effects.^406,407^

Mechanical signal transduction has been studied in detail in hair cells from the inner ear. While it has long been known that hair cells are able to sense tiny mechanical displacements, the responsible transducer in mammalian cells, Piezo1, was only discovered in 2010.^408^ Piezo1 is a calcium-permeable channel that is ubiquitously expressed throughout various cell types in the body, including neurons in the brain,^409^ and plays crucial roles in the development of organs, cell migration, maintenance of cell layers, as well as many other processes. Hence, to achieve cell-specific neuro-mechanical stimulation, it may be possible to express a modified Piezo1 channel genetically with an extracellular handle onto which a transducer can couple to provide controlled mechanical stimulation to the ion channel. There are other mechano-sensitive ions channels, such as large conductance mechanosensitive ion channels (MscLs),^410^ which have also been used for interfacing neurons.^411^

### Nanoparticles as Transducers for the Stimulation of Ion Channels

The complexity of the brain, with tens of billions of neurons interconnected in a nearly endless network, makes controlled stimulation an exceptionally demanding task. Technological advances in measurement techniques and probes have triggered major breakthroughs in neuroscience. The utilization of nanotechnology may potentially help to decipher the processes within the neuronal network. Nanoscale probes have several advantages in comparison to bulky electrodes that are typically used for the measurements and stimulation of neurons. Their similar size relative to individual cell bodies and nerve fibers enables localized activation (in case the NPs could be directed to precise positions) and prevent the activation of surrounding neurons. Furthermore, as the bending stiffness scales with the cube of material thickness, nanoscale materials minimize the apparent mechanical mismatch between brain tissue and engineered material, reducing the risk for glial scar formation, which would lead to the displacement of the probe from the neuron. Downsizing materials to the nanoscale results in new physical properties which can be utilized for signal transduction across different energy regions.^336,412−414^

One great potential of NPs lies in their function as transducers. As discussed above, there are voltage-, light-, heat-, mechanically, ligand-gated, etc. ion channels. However, it would be highly challenging to stimulate a mechanically gated ion channel directly deep inside the brain. On the other hand, a magnetic NP attached to a mechanically gated ion channel would enable transducing *via* magnetic stimulation (*i.e.*, alternating magnetic fields) into mechanical movement, which then, in turn, would trigger the ion channel. Inorganic NPs offer great potential, as they can be designed to interact with different stimuli, such as light, magnetic fields, etc. with high cross sections. A list of how NPs can be used as transducers is given in Table 1. Next, we discuss transduction mechanisms relevant to neuronal excitation. In addition to their function as tranducers, NPs can often play dual roles, allowing for both imaging and neuroprotection.^415−417^

**Table 1:** Transducing Modalities to Trigger Ion Channels

ion channel is gated by	external stimulus
electric voltage	electric voltage
electric voltage	optical
optical stimulus (light)	optical
thermal stimulus (heat)	optical
thermal stimulus (heat)	magnetic
mechanical stimulus	optical
mechanical stimulus	mechanical
mechanical stimulus	magnetic
chemical stimulus (ligand)	chemical

### Photoelectric Stimulation Using Nanoparticles

Light can create electron–hole pairs in semiconductors, leading to local potential differences, for example, used for photoelectrochemical sensors.^418−423^ Consequently, it should be feasible to use the electric potential created in photoexcited semiconductors to trigger voltage-gated ions channels. Likewise, also in plasmonic NPs, light-generated separation of charge carriers is possible, which also can be transferred to the environment, *e.g.*, so-called hot electrons.^424^ Successful proof-of-concept has been reported for this type of stimulation in cell culture systems.

Neuronal cells were grown on NP films, and upon photoexcitation, the accumulated charges at the NP/neuron interface led to the activation of voltage-gated ion channels, resulting in measurable membrane hyperpolarization or depolarization. Tailoring the degree of depolarization and hyperpolarization determines if an action potential (AP) is triggered (in the case of depolarization) or if an AP can be inhibited due to hyperpolarization. A classic example is the utilization of a photoelectrode prepared by a layer-by-layer approach^425^ using HgTe NP films with poly(dimethyl-diallyl-ammonium-chloride) (PDDA) on an indium–tin-oxide (ITO, InSnO_*x*_) substrate.^426^ Neuroblastoma glioma cells were chosen as model cells (note that glioma cells are *not* neuronal cells) and grown on the NP film. Although no direct opening of ion channels during photostimulation with a 532 nm laser with 800 mW/cm^2^ was observed, the extrinsic current flowing across the membrane led to measurable APs.^426^ The specific opening of K^+^ ion channels leading to hyperpolarization was demonstrated upon illumination of prostate cancer cells grown on a CdTe NP film. Cortical neurons grown on a CdSe NP film rather showed depolarization with significant production of APs.^427^ The immobilization of NPs on a glass micropipette tip enables better control over the NP/neuron proximity, and photostimulation led to hyperpolarization of the neurons.^427^ Due to their intrinsic toxicity, Cd-based NPs may not be the ultimate choice *in vivo*, though appropriate coatings can significantly block the release of toxic Cd^2+^ ions;^428,429^ there are more biocompatible semiconductor materials available.^430^ In general, for photoelectrode-neuron devices, the hyperpolarization strength is directly proportional to the photocurrent.^431^ As the photocurrent is dependent on the absorbance cross-section of the NPs used and the light intensity, the excitation wavelength is typically below 500 nm, which is not an appropriate range of wavelength for efficient tissue penetration. In another example, type-II InP/ZnO NPs were successfully integrated into a photoelectrode device, which served as a substrate for the growth of PC12 cells differentiated *via* nerve growth factor treatment. Low-energy light pulses of 4 μW/mm^2^ induced hyperpolarization and also firing of APs was observed.^431^ InP NPs were also used to build a biocompatible quantum funnel to direct the charge carriers to the bionanojunction for neuronal photostimulation. The quantum funnel is composed of three differently sized InP/ZnS NPs that engage in subsequent Förster resonance energy transfer to transfer the energy from one NP layer to the other, resulting in larger photocurrents compared to single-layer photoelectrodes.^432^ A valid application of NP-based photoelectrodes in neuroscience is their utilization for artificial vision. Bareket *et al*. immobilized CdSe/CdS core/shell NPs as light absorbers on a film of carbon nanotubes for optical stimulation of light-insensitive neuronal tissue. The highly porous structure formed by the carbon nanotubes enabled excellent coupling of the neuronal tissue and the carbon nanotubes themselves served as microelectrodes for the NP.^433^

Despite the obstacles needed to be overcome before NPs’ potential for stimulation of neuronal activity can be fully explored, the applications and the optoelectronic properties of NPs make them a promising tool for neuroscience. NP-based photoelectrodes may be at the forefront for wireless connections of the human brain with computers for neuroprosthetics for artificial vision. Fabrication of small, targetable, and remotely addressable electrodes should aid the development of future therapeutic tools for neuronal injury and sensory deficits.

Piezoelectric materials have been used for the same purpose.^434,435^ By growing them as sheets, larger areas can be covered, enabling improved interfacing to neuronal cells.^435^

### Photooptical Stimulation Using Nanoparticles

As discussed above, microbial light-gated ion channels work well in heterologous expression systems such as mammalian neurons. Concerning targeted stimulation of neurons in the brain, however, they have a major drawback. Most natural light-gated ion channels operate with blue/green light, wavelengths that are strongly scattered in brain tissue.^436,437^ In addition, high-intensity blue light may harm neurons and cause cytotoxic effects. To achieve the same performance using excitation wavelengths of 550 nm or even 650 nm, the light intensity needs to be increased by 3.5× and 30× , respectively. These intensities can be problematic for cell survival.^431^ Thus, to be able to access deep brain areas, the insertion of artificial light sources^438,439^ is necessary, which can cause inflammation or scarring of brain tissue similar to implanted microelectrodes, defeating the concept of noninvasive light stimulation. A common strategy to improve penetration depth is the use of fluorophores that emit in the near-infrared (NIR; 750 to 1000 nm) or short-wave infrared (SWIR; *ca*. 1000 to 1700 nm) or can transduce NIR light to visible light to modulate classical opsin-expressing neurons. NIR-activatable optogenetic tools utilize NIR absorbers, commonly NPs, to convert NIR light to heat to activate thermosensitive proteins.^440^ Examples of NIR optogenetic tools for light-controlled cell signaling, gene expression, and protein localization utilize organic fluorophores probes and tools based on Chrimson variants of channelrhodopsins^441^ or phytochromes with absorption and emission bands in the wavelength regions of *ca*. 500 to 700 nm and 650 to 900 nm, respectively.^379^ However, the absorption, fluorescence excitation, or action spectra of red-shifted variants of rhodopsins still fall short in the NIR and SWIR optical window, where tissue penetration is optimal.^442^ Two-photon excitation in the NIR range allows for deeper penetration of light into the tissue, although *in vivo* stimulation of channelrhodopsin-expressing neurons is eventually limited by light scattering^443^ and by NIR-induced tissue heating.^444,445^ Nanoparticles have been shown to possess large two-photon absorption cross sections, 2–3 orders of magnitude larger than organic dyes or channelrhodopsins, and could significantly increase the depth of two-photon stimulation in the brain.

Most organic fluorophores are not excitable in the NIR and are only weakly emissive in the NIR, particularly in the SWIR. They are easily outperformed by nanoscale emitters, *e.g.*, semiconductor quantum dots or rare earth (RE^3+^)-based NPs (RE = rare earth).^446−448^ Exploitation of different types of nanomaterials is anticipated to solve some of the challenges encountered in optogenetics, from the delivery/expression of the optogene to the stimulation/inhibition and follow-up sensing of neuronal activity. Optical activation and manipulation in deeper tissue can be addressed, *e.g.*, with the aid of semiconductor quantum dots and RE^3+^-based NPs, *i.e.*, the stimulation/inhibition of the light-gated channels, strategies described in detail in the following. The readout of neuronal activity and optogene delivery can be tackled, *e.g.*, with nanosensors such as voltage-sensitive quantum dots and plasmonic nanostructures or ion-sensitive luminescent nanomaterials and nanocarriers,^440,449,450^ which we will describe in the section on NP-based optical readout. Of particular interest for optogenetics are RE^3+^-doped upconverting (UC) nanoparticles (UCNPs), which consist of a host material of low phonon energy such as a fluoride or oxide matrix, *e.g.*, NaYF_4_, SrF_2_, CaF_2_, or Gd_2_O_3_, doped with a single type or different trivalent RE^3+^ ions (codoped UCNPs). In commonly used codoped UCNPs, one type of RE^3+^ dopant acts as a sensitizer that absorbs NIR light and transfers this excitation energy after multiple random energy migration processes among neighboring sensitizers to a neighboring RE^3+^ activator (X^3+^) in sequential steps (energy transfer, ET; upconversion, UC; energy transfer upconversion, ETU).^451,452^ The most efficient UCNPs obtained to date are hexagonal NaYF_4_ doped with the sensitizer Yb^3+^ excitable at 980 nm and the activators Er^3+^/Tm^3+^/Ho^3+^. Typically, molar fractions of 5–30% for Yb^3+^ (mostly 18–20% with Er^3+^ or 25% with other activators) are used together with 1–3% Er^3+^ (commonly 2%), 0.1–1% Tm^3+^ or 0.5 or 1% Ho^3+^. Also, Nd^3+^ can be utilized as a sensitizer, which is excited at 808 nm, where water absorption and thus heating effects are less pronounced.^453^ UCNPs have the capability to convert multiple NIR photons into shorter wavelength ultraviolet (UV), vis, or NIR UC luminescence (UCL; NIR-to-UV/vis/shorter NIR conversion) by successive absorption of two or more NIR photons by a system of real energy levels of RE^3+^ ions.^447,454−463^ The ladder-like electronic energy structure of the lanthanide ions leads to the emission of a multitude of sharp and characteristic emission lines with microsecond lifetimes in the UV/vis/NIR, defined by the RE^3+^ dopants, and enables the precise tuning of the emission wavelength by controlled lanthanide ion doping.^464^ Moreover, UCNPs can also be luminescent in the SWIR (down-converted luminescence, DCL).^447,465−469^ For optogenetics, the spectral shifting ability of UCNPs^380,469−471^ provides the following advantages: (i) greater penetration depth of light, potentially entirely free of implanted fiber optics, due to their NIR excitability, (ii) localized delivery of excitation light with high spatiotemporal precision provided by the photons emitted by the UCNPs, particularly in the UV and vis wavelength region restricted to the targeted neurons or neuronal substructures (*e.g.*, terminals), and (iii) potential for optical imaging for guided optogenetic stimulation. This localization is of great interest for multiple areas of biomedicine, which particularly include bioimaging, neuromodulation, and therapeutic applications such as advanced oncotherapy.^380,469−473^ Increasing numbers of studies suggest the use of UCNPs for optogenetics and regenerative medicine, utilizing Tm^3+^- and Er^3+^-*co*-doped UCNPs commonly relying on the β-NaYF_4_ host matrix and the sensitizers Yb^3+^ and less commonly also Nd^3+^, excitable at 980 and 808 nm, respectively.^470,471,474−478^ The blue emission of UCNPs doped with the activator Tm^3+^ matches the maximum absorption of ChR2 for neuronal activation, while the green emission originating from the activator Er^3+^ is compatible with activation of halorhodopsin (NpHR) or archaerhodopsin (Arch) for neuronal inhibition.^471^ An example of cellular optogenetics presents the *in vitro* targeting of cells, expressing ChR2, with Tm^3+^-doped UCNPs that produce upconversion luminescence (UCL) at ∼470 nm, exciting ChR2.^470^ In combination with Er^3+^-doped UCNPs with their Er^3+^-related UCL (peak at ∼550 nm) absorbed by NpHR, could be exploited for the specific activation of cells expressing either ChR2 or NpHR with two different types of codoped UCNPs. Also the stimulation of deep brain neurons was achieved in living mice with blue emitting NaYF_4_:Yb,Tm core/shell UCNPs and green emitting NaYF_4_:Yb,Er core/shell UCNPs of different particle architectures in living mice.^471^ This combination was used to evoke dopamine release from genetically tagged neurons in the ventral tegmental area, inducing brain oscillations through activation of inhibitory neurons in the medial septum, silence seizure by inhibition of hippocampal excitatory cells, and trigger memory recall.^471^ Thus, a combination of bright and small UCNPs of different chemical compositions with optimized UCL, varying in the chemical nature of the activator ion, could be utilized for multiplexed targeted, cell-specific, imaging-guided optogenetics. Such a multiplexed optogenetic stimulation of neurons with spectrally distinguishable NaYF_4_-based UCNPs doped with Tm^3+^ or Er^3+^ has been used to stimulate neurons expressing different ChRs, here ChR2 or C1 V1.^476^ Recently, a series of core/shell and core/shell/shell UCNPs of different chemical compositions and particle architectures relying on NaGdF_4_:Yb,Tm,Ca or NaGdF_4_:Yb,Er,Ca cores with the sensitizers Yb^3+^ and Nd^3+^ have been used to manipulate motor behavior of the nematode *Caenorhabditis elegans*.^474^ These NPs effectively activated Chrimson-expressing inhibitory GABAergic motor neurons, resulting in a reduced action potential firing in the body wall muscle and resulting in locomotion inhibition. Moreover, NIR-mediated control of stem cell differentiation has been shown with multishell UCNPs bearing photoswitching polymer-capping ligands to enable the spatiotemporally controlled release of small molecules like stem cell differentiation factors, thereby guiding neuronal stem cell differentiation in a highly controlled manner.^479^

**Figure 17:**
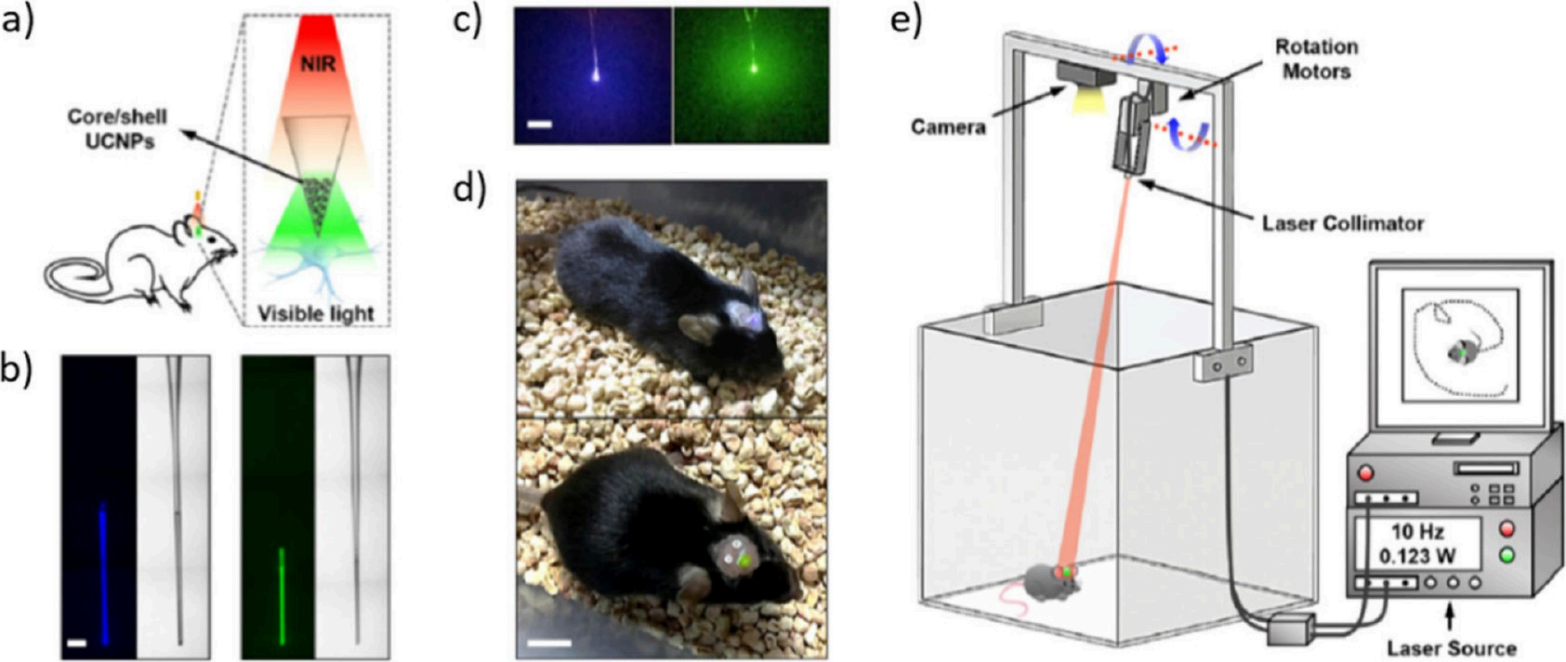
Demonstration of upconverting nanoparticles (UCNPs) as transducers for converting NIR light into green light for exciting light-gated ion channels. (a) Schematic of tetherless near-infrared (NIR) optogenetic control of brain activity using fully implantable upconversion microdevices. (b) Bright-field and fluorescent photographs of the implantable micro-optrodes containing UCNPs doped with Tm^3+^ (blue) or Er^3+^ (green). Scale bar, 500 mm. (c) Fluorescent images of the operating UCNP microdevices (Tm^3+^-doped, blue; Er^3+^-doped, green) excited by near-infrared (NIR) light. Scale bar, 2 mm. (d) Images of animals implanted with different types of micro-optrodes containing Tm^3+^-doped (top) or Er^3+^-doped (bottom) UCNPs. Scale bar, 1 cm. (e) Instrumentation design of a robotic laser projection system for automatic and consistent NIR irradiation of the heads of behaving animals. Figure and caption taken with permission from Wang *et al*.^482^ Copyright 2017 Elsevier.

Upconverting NPs have been used as active media in implantable devices for wireless optogenetic and neuronal modulation to provide an effective and interference-free alternative for remote brain stimulation and inhibition in neuroscience research.^441,480^ Here, different types of UCNPs with the activators Tm^3+^ and Er^3+^ have been exploited, including dye-sensitized UCNPs with improved NIR absorption efficiencies, *i.e.*, absorption cross sections, to overcome the generally low absorption cross sections of UCNPs caused by the forbidden nature of the underlying optical transitions.^481^ Thus, the brain activity of rats could be monitored by means of an implanted UCNP-optrode consisting of β-NaYF_4_:Yb, Er (Tm), see Figure 17.^482^ Here, the NIR energy of UCNPs is converted into visible light that stimulates neurons expressing various opsin proteins. In this way, spiking activity can be reliably triggered in rat brains. Using robotic laser projection systems, it was then possible to modulate activity in different brain regions, such as the striatum, ventral tegmental area and visual cortex, thus controlling stimulus-stimulus and stimulus-response associations of the freely moving animals.

The neuronal activity of living brain slices has been modulated with β-NaYF_4_:Yb, Tm-UCNPs using azobenzene-based photoswitches.^483^ Polyacrylic acid-coated β-NaYF_4_:Yb, Er-UCNPs conjugated to concanavalin A (ConA) have been injected into mice retina, enabling NIR light sensitization of photoreceptors and even pattern recognition. Such nanoantennas allow for an extension of vision from the visible into the NIR range.^484^ A recent example of a UC wireless device presents a flexible optrode UC system fabricated from biocompatible thermoplastic polypropylene mixed with β-NaYF_4_:Yb, Er-UCNPs that convert NIR photons from a 980 nm laser diode (20 mW/mm^2^) to visible light for the optogenetic manipulation of spinal cord tissues, enabling the activation of spinal cord neuron and evoking hindlimb muscular activity in mice.^485^

Although these examples demonstrate the considerable potential of NIR-excitable and spectrally shifting UCNPs for optogenetics, there remain challenges to be overcome for their use *in vivo*.^486^ General disadvantages of nanomaterials that can hinder their application in optogenetics are related to their potential toxicity, the nonbiodegradable nature particularly of inorganic NPs,^487^ their relatively complicated clearance pathways,^488^ and their targeting efficiency^489^ requiring optimized surface chemistries and control over the number of targeting ligands and their orientation.^490^ Particular challenges of UCNPs are related to their relatively low absorption cross sections, their relatively low UCL quantum yields, which require optimized particle architectures, doping ion concentrations, proper surface protection, and to tackle recent concerns regarding their chemical stability.^464^ This latter issue is especially important in the current development of tiny UCNPs for biomedical applications.^491^ UCNPs are photochemically stable and can be used for imaging applications at high excitation power densities, in the MW/cm^2^ regime, including super-resolution nanoscopy and real-time upconversion microscopy that enable visualization of intraneuronal motor protein transport.^492,493^ Although such NPs have previously been regarded as chemically inert and biocompatible,^494,495^ concerns have been raised regarding their potential cytotoxicity. For example, several research groups have observed the release of potentially toxic RE metal and fluoride ions from host matrices such as NaYF_4_ in aqueous media and particularly under high dilution conditions.^496−498^ This issue might be addressed with suitable surface coating,^499,500^ utilizing, *e.g.*, tightly bound amphiphiles^501,502^ or a sufficiently thick silica shell.^498^ The former requires the development of suitable conjugation strategies for the development of targeted probes (which are also able to cross the blood–brain barrier, BBB) and the latter will increase NP size. For UCNPs embedded into devices like implantable optrodes, which require local surgery, this should not present a problem as long as NP leakage can be prevented. The greatest challenge, however, remains the development of targeted probes that are not recognized by the immune system and, moreover, are able to cross the BBB. Alternative nanocoating strategies are being intensively pursued.^503^ Thus, far, a few mechanisms have been proposed for NPs to cross the BBB, including possible direct intrusion and receptor-mediated transport;^504^ see also the BBB section in “Improving Cell-Electrode Interfaces”. Receptor-mediated, including hormone receptor-mediated, transport of NPs across the BBB using proteins such lactoferrin, apolipoproteins, and insulin are promising approaches that need to be applied to UCNPs.^505−508^ Super-resolution microscopy can help here to analyze the precise locations of the NPs.^509^ Overall, photostimulation using UCNPs allows for exciting therapeutic applications, especially in oncotherapy such as manipulation of the tumor microenvironment, photodynamic, photothermal, and immunotherapy. However, there are few reports of mammalian studies in this regard.

### Photothermal Stimulation Using Nanoparticles

The photothermal pathway can be used by plasmonic heating of NPs from gold,^510−512^ copper sulfide,^513^ or other nanomaterials. Heating may interfere with neurovascular coupling and the permeability of the BBB, so it may be difficult to discern truly channel-specific photothermal cell activation from nonspecific influences by blood-borne substances.^514^ Also, cortical astrocytes have been shown to respond to the heat generated by infrared (IR) two-photon excitation^445,515^ with aberrant intracellular calcium signals.^444^

Local photothermal heating of plasmonic NPs has led to a variety of biomedical applications, including photothermal therapy (PTT) to destroy tumor cells.^516^ Here, NPs are covered with targeting moieties such as antibodies or aptamers, which bind only to distinct tumor cells carrying the complementary chemical functionality. Note that such active targeting is an important issue and there is also nonspecific uptake of NPs.^489^ Ideally, after internalization of the NPs and illumination, only the target tumor cells would be destroyed. This localization is possible since the absorption cross section of the plasmonic NPs is high, such that they can be selectively excited. If the plasmon frequency is tuned to the NIR-window of the tissue, between 700 and 900 nm, the NPs can be excited deep inside the tissue. This tuning can be achieved by, *e.g.*, using elongated gold nanorods Au nanorods (NRs) with an aspect ratio of *ca*. 3, where the plasmon frequency of the longitudinal collective oscillation of the valence electrons leads to an absorption band centered at 750 nm.^517^ Upon illumination of the Au NRs, the absorbed energy is eventually dissipated into heat, which can trigger the initiation of a cell death mechanism, apoptosis or necrosis.^518^ Such Au NRs functionalized with cationic protein/lipid complex have also been used to activate the thermosensitive cation channel TRPV1 in intact neuronal cells. Here, the localized photothermal heat mediated by the plasmonic Au NRs induced the Ca^2+^ influx solely by TRPV1 activation.^511^ However, if local heating were to be used for temporal changes of the temperature in the vicinity of ion channels, the location and number of NPs (*e.g.*, Au NRs) need to be carefully adjusted, as do the illumination conditions. For example, the local temperature change mediated by plasmonic heating can lead to the formation of transient pores in the membrane even in the absence of any temperature sensitive ion channels.^519^ In addition, NPs other than those based on Au have been used.^415,520^

One can distinguish between photoexcitation of the NPs with short laser pulses or with continuous wave (cw) lasers. In the case of pulsed lasers, a temporal temperature profile could be established, and a sequence of laser pulses could lead to periodic changes in the ion channel conductance. It is important to adjust both the energy deposition into the NP by the laser pulse, and its subsequent dissipation into heat, which is eventually transferred to the proximate region. This process can be divided into several subsequent steps.^521^ The first step is the absorption of light through the collective excitation of free electrons. These plasmons dephase rapidly on a time scale of tens of femtoseconds, giving rise to the broad bandwidth in the plasmonic UV–vis or NIR absorption spectra. In the next hundreds of femtoseconds, the hot electrons thermalize by scattering processes and electron–phonon coupling leads to a temperature change of the lattice itself, on the time scale of picoseconds. The hot NPs then couple to and heat the environment within hundreds of picoseconds. Under cw excitation, one can reach steady state conditions between excitation through photons and dissipation into heat, leading to a constant temperature profile shortly after the start of illumination. The resulting spatial temperature decay away from the NP is exponential with a decay width on the range of the radius of the NP itself, *i.e.*, several nanometers.

For pulsed excitation, there is an additional temporal temperature profile, where the temperature rise is strongly dependent on the length of the laser pulse and its energy. For example, illumination with femtosecond laser pulses can lead to rapid temperature rise, which finally results in a pressure wave traveling away from the nanostructure to induce the formation of vapor bubbles.^522^ On the other hand, if the laser pulse is in the nanosecond range, all the dissipation processes described above happen within the pulse period. In this case, the absorption of photons and the dissipation of the energy into heat happen in parallel, which leads to a constant temperature profile after a short induction period. Besides this temporal temperature profile, the spatial temperature profile in the vicinity of the NP depends on the length of the excitation pulse. The spatial temperature profile decays more rapidly into the surrounding for illumination with short laser pulses. For example, the radial temperature decrease follows a 1/r^3^ dependence under illumination with short laser pulses, while the steady-state profile under cw excitation has a 1/r dependence. Hence, a larger volume around the NPs is heated with cw excitation.^523^ Local heating to trigger ion channels can more easily be achieved by quasi cw conditions, *i.e.*, by using excitation periods of hundreds of nanoseconds to milliseconds.

This issue is especially important since the temporal temperature change surrounding the NP should exceed the ion diffusion time to stimulate neuronal action. For example, it has been shown that the membrane current can be activated by heat (50 °C) with a mean time to half activation of about 35 ms.^524^ While experiments to trigger neuronal action with sequences of illumination pulses remain to be done, several simulations of the spatiotemporal evolution of temperature have been presented.^525^ These simulations suggest that the realization of programmed stimulation of neuronal action indeed could be realized by millisecond illumination of Au nanorods in the vicinity of ion channels, to induce capacitive currents thermally and to alter the membrane potential.

Photothermal heating can also trigger Ca^2+^ signaling. Photothermal activation of endocytosed NPs enables precise manipulation of single endolysosomes. These organelles have gained much attention recently, on the one hand, because they are involved in multiple crucial cellular functions, such as (i) endocytosis, (ii) exocytosis, (iii) autophagy/mitophagy, (iv) nutrient sensing, (v) plasma membrane repair, or (vi) exchange of molecules with other organelles, *e.g.*, the endoplasmatic reticulum (ER; reviewed in Li *et al*.^526^). On the other hand, there is increasing evidence that many of these functions are controlled by ion channels localized in the endolysosomal membrane, *e.g.*, transient receptor potential channels–subfamily mucolipins (TRPML), two-pore channels (TPC), or purinergic P2 × 4 channels (reviewed in Li *et al*.^526^). Lysosomal Ca^2+^ release in glutamate-evoked excitotoxicity is involved in hippocampal neurons.^527^ Though their application in brain or peripheral nervous tissue may be difficult, in cultured cell experiments can be much better controlled for neuronal cell types, *e.g.*, neurons or astrocytes, or other cell types that endocytose the NPs.

**Figure 18:**
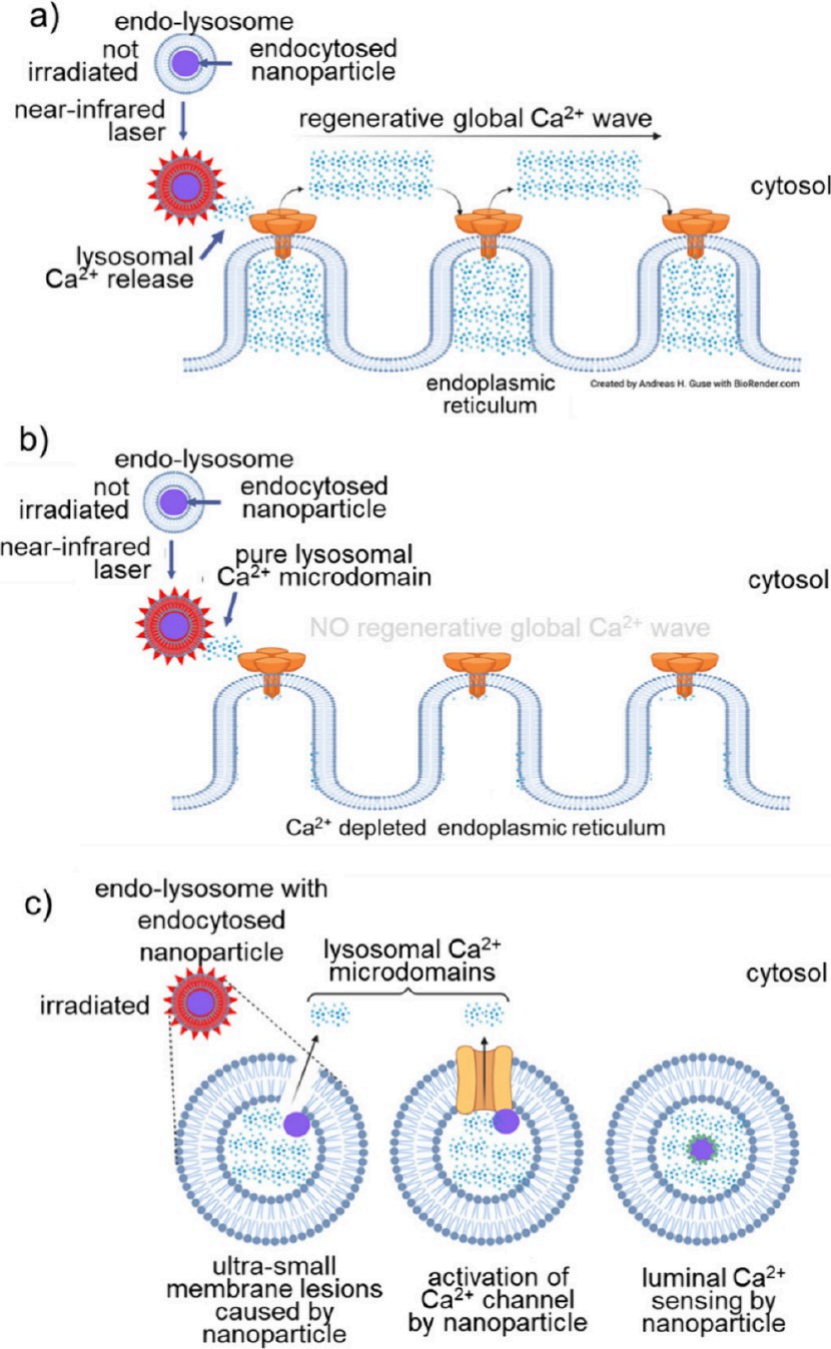
Amplification model for endolysosomal Ca^2+^ signaling. (a) Laser irradiation in the near-infrared results in small and brief local Ca^2+^ signals that require amplification for global Ca^2+^ signaling, here as a regenerative Ca^2+^ wave caused by Ca^2+^-induced Ca^2+^ release (CICR) in MCF-7 cells. (b) Depletion of the endoplasmic reticulum results in pure lysosomal Ca^2+^ microdomains due to lack of amplification. (c) Future applications for endolysosomal nanoparticles: photothermally induced ultrasmall lesions (left), activation (or inhibition) of Ca^2+^ signals (middle), or luminal Ca^2+^ sensing (right).

Zhu *et al*. recently described a method to load cellular endolysosomes with polymer capsules containing integrated plasmonic NPs.^528^ Upon heating the particles with IR light, an increase of the free cytosolic Ca^2+^ concentration ([Ca^2+^]_i_) was observed. Unexpectedly, though single lysosomes were heated, global Ca^2+^ waves traveling over entire cells were observed in both MCF-7 and HeLa cells.^528^ Since the volume of lysosomes is small, such global Ca^2+^ signals require amplification of the initial lysosomal Ca^2+^ release. For MCF-7 cells, Ca^2+^ entry phenomena were excluded as an amplification mechanism, but a regenerative Ca^2+^ wave caused by Ca^2+^-induced Ca^2+^ release (CICR) from the endoplasmic reticulum (ER) was observed (Figure 18a). For this experiment, MCF-7 cells were kept in Ca^2+^-free extracellular buffer to avoid activation of store-operated Ca^2+^ entry (SOCE), and the ER was depleted of Ca^2+^ by inhibition of the sarco/endoplasmic reticular Ca^2+^-ATPases (SERCA) by thapsigargin. Physiologically, SERCAs pump back released Ca^2+^ into the sarcoplasmic reticulum (SR) or ER; upon inhibition, luminal Ca^2+^ gets lost through leaky channels, resulting in low intraluminal Ca^2+^ concentrations. Under such experimental conditions, photothermal heating of single endocytosed NPs resulted in brief and small pure Ca^2+^ signals from a single lysosome (Figure 18b).^528^ An important limitation is the unknown mechanism resulting in lysosomal Ca^2+^ release; it may be due to ultrasmall lesions of the lysosomal membrane, however, heat-sensitive Ca^2+^ channels may also be involved. Also, the micrometer size of the capsules filling out and potentially also blowing up the lysosome, is not ideal to study such intricate signaling processes. Thus, future experiments with smaller NPs that do not enlarge endolysosomes and ideally are located in a small volume of the lysosomal lumen only, may refine the current view of lysosomal Ca^2+^ signaling (Figure 18c). Further, such smaller NPs may serve several functions: (i) controlled induction of ultrasmall lesions of the endolysosomal membrane, (ii) direct inhibition or activation of individual (lysosomal) Ca^2+^ channels (reviewed in Lee *et a*l.,^529^ or (iii) use of Ca^2+^ responsive NPs targeted to the lumen of single lysosomes by endocytosis to measure luminal [Ca^2+^] parallel to [Ca^2+^]_i_ (reviewed in Li *et al*.^530^).

For millisecond infrared pulses, action potentials can be induced *via* optocapacitive photothermal effects. Instead of the above-discussed mechanisms, where light-generated heat acts on ion channels, this effect is based on the observation that the membrane capacitance is temperature-dependent, *i.e.*, it increases with temperature.^531^ Changes in capacity d*C*/d*t* lead to a current (C = Q/U = = > d*C*/d*t* = (1/U)·d*Q*/d*t* = (1/U)·I; C = membrane capacity, Q = charge, U = membrane potential, I = optocapacitive current), in the present context termed optocapacitive current, which causes depolarization and thus can trigger an action potential.^531^ In this way, a light pulse creates a short heat pulse, leading to a temperature-dependent change in the membrane capacitance, ultimately causing an action potential by local depolarization of the cell membrane. This principle has been demonstrated in a range of applications.^532−535^

### Magnetothermal Stimulation Using Nanoparticles

In contrast to electric fields or light, magnetic fields penetrate easily through the skull and brain tissue. This makes them an attractive tool to deliver stimuli to neurons. Direct modulation by magnetic fields has recently been reported.^536^ Alternatively, the magnetic field energy can be converted by an actuator to a secondary cell-specific stimulus, such as heat. Among the TRP family, several are known to respond to rapid changes in temperature and are thus preferred mediators for magnetothermal activation.^403−405,407,537−542^

This concept was realized as magnetothermal neuronal stimulation - an approach combining the genetic expression of temperature-sensitive ion channels and superparamagnetic NPs that convert the energy from alternating magnetic fields into local heat.^406^ This approach provided tetherless magnetic activation of specific brain circuits in a freely behaving animal.^403,407^ The temperature-sensitive cation channel TRPV1 was used to activate (depolarize) the neurons, as upon activation calcium or sodium ions enter the cell, and the channel activates at moderated temperatures. While the midpoint of activation is 42 °C, a sufficient number of channels are already activated at 39 °C to trigger neuronal firing.^407^ In addition, TRPV1 is only weakly expressed in the brain, so off-target activation is minimized.^543,544^ In an attempt to develop magnetothermal silencing, channels are being explored that would hyperpolarize the neurons. The chloride-permeable ANO1/TMEM16A channel and the potassium efflux channel TREK1 have both been shown to be triggerable *via* local magnetic NP-based hyperthermia.^545^

One disadvantage of magnetothermal stimulation is that heating is relatively slow, limiting the responses of animals to magnetothermal stimulation to on the order of ten seconds.^407^ Significant research has gone into optimizing the thermal energy conversion of the superparamagnetic NPs and tuning them to specific field frequencies, while keeping the NPs small enough to be superparamagnetic and to diffuse freely in tissue.^546−548^ Hence, it is unlikely that the speed of heating can be increased enough to stimulate individual action potentials thermally, as they have durations shorter than 10 ms. Such speed could be achieved, if it was possible to transfer the thermal energy directly from the NP to the ion-channel, *i.e.*, minimizing parasitic water heating. How much of the surrounding water is heated is a matter of active debate and the answer depends on the system studied. Some dispute that the heating can be well confined,^549,550^ while others argue that binding the NPs to the cell membrane leads to cell-specific heating with a kinetic signature of local and not volumetric heating,^407^ or that the temperature near the NPs is significantly higher than in the water.^551^

Current magnetothermal stimulation methods are well suited to study memory formation, deletion or modulation in the brain, as those processes are thought to require repeated or continuous stimulation (or suppression) for many seconds or minutes.^552,553^

### Magnetomechanical Stimulation Using Nanoparticles

Recent efforts facilitated magnetomechanical neuronal stimulation, which is another nanotechnology-enabled neuromodulation with significantly faster response times. One of the intrinsic mechanical force transducers is in the hair cells of the inner ear. The stereociliary structure of hair cells oscillates in response to mechanical sound waves, whose frequencies range from a few to hundreds of thousands Hz depending on the animal species.^554^ There has been a search for the responsible mechanosensitive ion channels transducing mechanical force into electrochemical signals,^555,556^ and the recently discovered transmembrane channel-like (TMC)1 and 2 are thought to be the most probable candidates.^557^ There are other suspected mechanically gated channels discovered in eukaryotic cells, including TREK, TRAAK, TRPV, and TMEM channels. However, their structure, function, characteristics, and orthogonality to mechanical force are not yet elucidated and remain under investigation. The hitherto *bona fide* mechanosensitive ion channels known are Piezo1 and 2, which are pressure sensor proteins responsible for touch, pain, heart beating, and blood flow regulation.^558^

Utilizing these mechanically gated ion channels was proposed for magnetic force-mediated stimulation by magnetic NPs to induce the on-command gating of the channel by the transduction of mechanical force into electrophysiological responses. One of the studies to demonstrate this idea for neuronal stimulation utilized genetically encoded TRPV4 ion channels ligated to a magnetic protein, ferritin, named as “Magneto”.^559^ However, subsequent studies brought its working mechanism into question, since ferritin is weakly magnetic and it would be difficult to generate adequate force to stimulate the channel.^560−562^ Instead, the idea of magnetomechanical stimulation was demonstrated by using magnetic actuators with stronger magnetic moments to provide forces in the piconewton range, sufficient for channel gating.

To achieve cell-specific mechanical neuronal stimulation, it is necessary to express a modified Piezo1/2 channel genetically with an extracellular handle onto which the magnetic transducers can be coupled. The second hurdle for magnetomechanical neuronal stimulation is the need for the magnetic actuator NP to have a permanent magnetic moment, which requires 10-fold larger NPs than for heating, *ca*. 200 nm *versus* 20 nm. NPs with permanent dipoles also tend to interact and aggregate. Several groups developed transducers to circumvent these challenges, as described below.

For *in vitro* studies, Gregurec *et al*. fabricated a 200 nm thin magnetic disc with a vortex magnetic state for stimulation of intrinsically expressed Piezo2 ion channels in spinal cord neurons.^563^ These magnetic discs are nonspecifically attached to the cell membrane, and the application of low frequency alternating magnetic fields successfully results in flipping motion of discs on the membrane to exert mechanical strains for neuronal stimulations. Another *in vitro* demonstration was performed by magnetic torquer (m-Torquer),^564,565^ which is a 200 nm sphere composed of multiple magnetically anisotropic NPs. This design creates NPs with a permanent magnetic moment so that they will rotate with the rotation of an applied external static magnetic field. Short bursts of a rotating external magnetic field twist the NPs, which when tethered to the mechanical channels, exert a torque on the respective channels. The m-Torquer was selectively linked to Piezo1 *via* antibody–antigen binding and externally manipulated by the pulsed applications of a rotational magnetic field. The force activates Piezo1 to invoke Ca^2+^ influx, then further activates either to elicit action potentials or to regulate downstream signaling pathways toward the Ca^2+^ responsive gene expression promoter, NFAT, to induce protein expression of pre-encoded genes of interest.^564^ The magnetic field is applied *via* a circular array of permanent magnets, which is briefly rotated to cause the stimulation. It does not require any tether or fiber into the brain of the investigated animal, and the design permits making the array diameter large enough to fit a human head.

**Figure 19:**
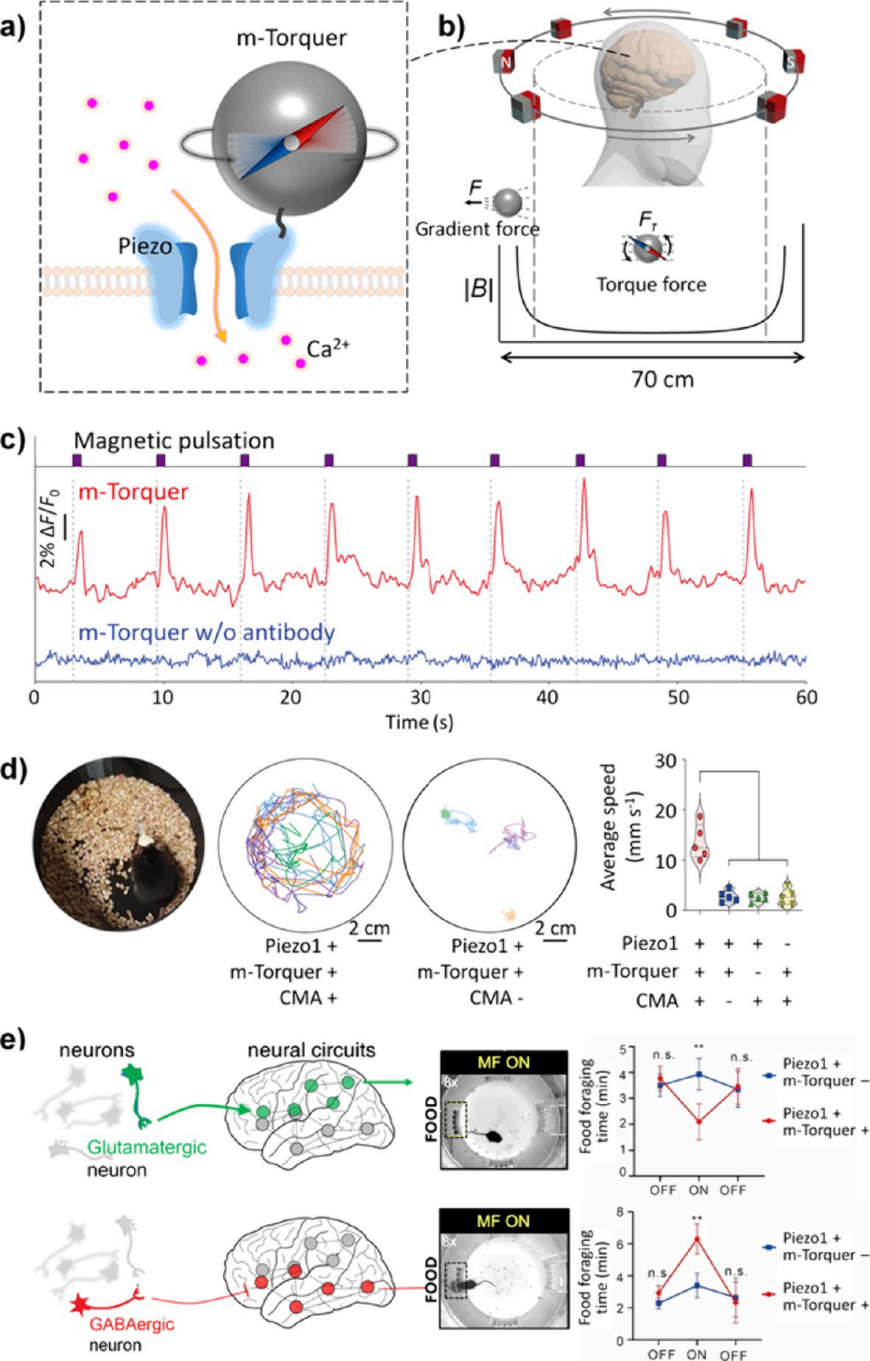
Magnetomechanical actuation with an m-Torquer nanoparticle for remote control of electrical activity in genetically engineered neurons. (a) Mechanical torque force generation by an m-Torquer nanoparticle under a rotating magnetic field for the activation of a mechanosensitive ion channel, Piezo1, to transduce Ca^2+^ eliciting action potentials. (b) Long working ranges under constant magnetic field, |*B*|, suitable for large-scale *in vivo* studies. (c) Temporal control of Piezo1 activation in mouse cortical neurons *via* magnetic fields. (d) Mice motional behavior control by m-Torquers injected into the motor cortex M1 region of mouse brain. The motion trajectory shows the increased movements of a mouse treated with Piezo1 and m-Torquers with circular magnetic array (CMA) rotation. Figure adopted with permission from Lee *et al*.^565^ Copyright 2021 Springer Nature. (e) Neuron-type specific magnetic stimulations for neuronal circuitry control. Stimulation of glutamatergic and GABAergic neurons in lateral hypothalamus induces increase and decrease of food foraging behavior in mice, respectively. Figure adopted with permission from Choi *et al*.^567^ Copyright 2024 Springer Nature.

For impactful neuroscience, magnetomechanical stimulation should be applicable to *in vivo* studies for arbitrary targets in any place of the brain, wireless and remotely.^566^ The aforementioned m-Torquer system demonstrated by Lee *et al*. meets these requirements by utilization of a torque force at a constant magnetic field (see Figure 19).^565^ The m-Torquer is designed to have high magnetic moments and the rotation of the magnet array makes the m-Torquer rotating in-phase, generating a few pico-Newton torque force. Piezo1 ion channels were delivered *via* viral vectors and genetically encoded to the target neurons. The torque force transduction in magnetomechanical stimulation is direct, fast, and localized and temporal resolution can be as fast as on the milliseconds timescale. The m-Torquer system demonstrates the concept of magneto(mechanical)genetics powerful for being truly wireless and controlled at a distance, a shortcoming in optogenetics. For example, Choi *et al*. showed the neuromodulation of specific neuronal circuitry in mouse brains, selectively activating excitatory or inhibitory neurons in deep brain.^567^ Such remotely triggered neuromodulation could validate effective neuroscience applications in cognition and social behavior of animals without limitations in numbers of animals and spatial confinement. Future targets include developing a wide variety of mechanically gated ion channels and methodologies for cell-type specific neuronal targeting for investigating neuronal circuitry and revealing brain functions. For example, ion channels capable of inhibitory functions and the expression of mechano-sensitive ion channels on specific types of neurons are essential. Once these assemblies are made, magneto(mechanical)genetics may become a highly useful tool for neuromodulation studies on a wide variety of animal types from rodents to primates and human in nontethered and remote ways.

### Optomechanical Stimulation Using Nanoparticles

Optical excitation can be converted into a mechanical signal in the form of soundwaves, commonly known as photoacoustics. The soundwaves can then trigger mechanosensitive ion channels.^568^

Mechanical stimulation could also be performed by the control of light using mesoporous silicon (see also the section “Advanced Test Platforms to Model Aspects of the Brain”). In aqueous electrolytes, mesoporous silicon can convert electrical signals to mechanical actuation directly and effectively.^569^ The voltage-strain coupling for this nanoporous system is three orders of magnitude larger than the best-performing ceramics in terms of piezoelectric actuation. The exceptionally small operation voltages (0.4 to 0.9 V), along with the sustainable and biocompatible base materials, make this hybrid material promising for bioactuator applications. Specifically, the emerging field of mechanical, acoustic, and opto-acoustic actuation of neuronal activity^570−572^ could profit from this material system.^569,573,574^ This approach is highly advantageous in comparison to classical piezoelectric ceramics, where the high and thus physiologically incompatible operating voltages make applications *in vivo* challenging, if not impossible.

### Nanoparticles for Transducing Electrical Signals into an Optical Readout

Nanoparticles can be used to transduce neuronal signals into the optical domain, such as *via* fluorescence. The primary readout of neuronal action is the membrane potential, or the ionic currents through the ion channels, both of which are electrical events. For example, voltage-sensitive dyes or protein constructs have been applied to monitor the membrane potentials of neurons, but voltage-induced changes in their luminescence are relatively modest and prone to photobleaching.^575^ Inorganic semiconductor NPs, on the other hand, are known for their photostability and optoelectronic properties, which can be modulated by external electric fields. Quantum dots (QDs), which are inorganic NPs of semiconductor materials, have emerged as promising functional nanomaterials for the real-time imaging of neuronal membrane potentials. They have distinct advantages over traditional voltage-sensing materials such as organic dyes^575,576^ or genetically encoded fluorescent proteins.^577^ Their advantageous photophysical and electronic properties include:^575,576,578^ (i) They have small sizes, on the scale of the plasma membrane (∼5 nm). (ii) They have bright, stable photoluminescence (PL) that can be modulated by applied electric fields.^579,580^ This effect, also known as the quantum-confined Stark effect (QCSE), includes a shift of the PL emission maxima, reduced PL intensity, and also influences the PL lifetime. (iii) They can be readily functionalized with membrane-targeting peptides or with other moieties using established bioconjugation protocols.^581,582^ (iv) Their large two-photon absorption cross section is helpful for deep-tissue imaging.^583,584^ Building on foundational studies that demonstrated the responsiveness of QDs to an applied electric field,^579,580,585^ several groups have shown the feasibility of implementing QD-based voltage sensing systems both *in vitro* and *in vivo*. Simulations have also shown that the superior brightness of QDs results in AP detection with substantially fewer indicator molecules than voltage-sensitive dyes.^580^

These NP-based sensing/imaging systems include QDs that are inherently voltage-sensitive as well as QD-bioconjugate systems that engage in charge and energy transfer. The Keyser group developed inherently voltage-sensitive CdSe/CdS and InP/ZnS core/shell QDs for imaging membrane potential changes in *Xenopus laevis* retinal ganglion cell axons, finding that QDs had up to 2-fold greater sensitivity than state-of-the-art genetically encoded calcium imaging proteins.^586^ However, the magnitude of the QCSE is dependent on the shape, size, and band gap fine structure of core/shell NPs.^585^ The band gap energy decrease, responsible for the observed redshift of the PL emission maxima, has a quadratic dependence on the field amplitude for symmetric NPs, whereas asymmetric NPs, like quantum rods, have linear dependences. This effect translates to a PL shift of a maximum 40 mV for symmetric and 100 mV for asymmetric NPs when experiencing an electric field equivalent to a neuron action potential. The shift of the rod-shaped NP emission maxima can be shifted either to the blue or to the red, depending on the orientation of the exciton dipole moment to the external electric field.^587^ Independent of the NP shape, an increase in size is coupled with increased polarizability and thus results in enhanced voltage sensitivity. The size-limiting factor for the utilization of NPs as voltage sensors is the thickness of the membrane bilayer, which is only 4 nm. Although larger NPs have increased QCSE, the probability of insertion into the membrane is decreased. Fine balancing between enhanced QSCE and the NP’s size capable of insertion into the membrane is required.^588^ Another important factor is the NP architecture in which core/shell NPs can be divided into type-I and type-II NPs depending on the band alignment between core and shell. Strong confinement of the exciton in the core in type-I NPs makes them less sensitive to external electric fields whereas type-II NPs provide increased charge separation of the holes and the electrons in spatially separated band energy minima, which reduces the Coulomb attraction and thus increases the sensitivity for QCSE.^579^ Studies have shown that elongated type-II NPs composed of ZnSe/CdS are the excellent candidates for membrane potential recording and stimulation due to the creation of a large dipole moment at small sizes and thus have the biggest changes in PL intensity, PL shift, and PL lifetime when exposed to external electric fields.^578,585,588^

**Figure 20:**
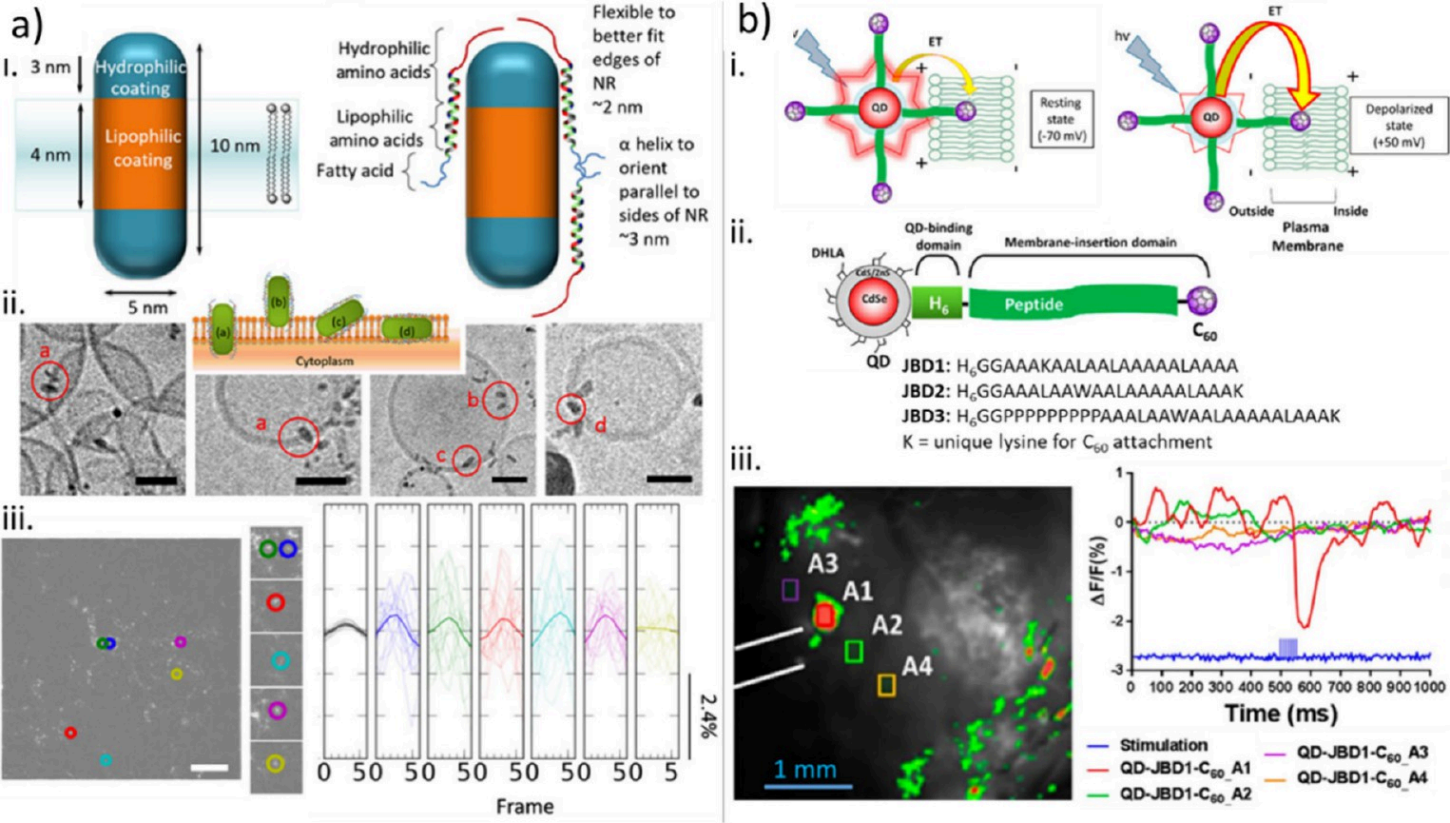
Quantum dot (QD)-based voltage sensing. (a) (i) Schematic of quasi type-II CdSe-seeded CdS QD nanorods decorated with amphipathic peptides. Rigid, lipophilic, α-helical regions are oriented parallel to the rod-shaped nanoparticle, whereas flexible, hydrophilic regions are oriented to and cap the nanorod ends. (ii) Schematic (top, center) depicting the potential membrane bilayer insertion orientations and cryo-scanning electron micrographs (bottom) of the NPs inserted into small unilamellar vesicles. The scale bars correspond to 30 nm. (iii) Spatially high-pass-filtered image of human embryonic kidney cells containing inserted rod-shaped NPs (left) and representative temporal, bandpass-filtered traces of changes in fluorescence normalized to the steady-state fluorescence (Δ*F*/*F*_0_) for each region of interest from (right). Bolded traces are the means of the 19 overlaid traces. The scale bar indicates 10 μm. Image taken with permission from Park *et al*.^589^ Copyright 2018 The Authors. (b) (i) Schematics depicting the mechanism of action of QD voltage sensing using electron transfer. At resting potential, the QD is bright and becomes dimmer upon membrane depolarization. (ii) Schematic of CdSe/CdS/ZnS core/multishell QDs conjugated to peptide-fullerene (C_60_) with the corresponding peptides tested. (iii) (left) Representative frame of mouse cortices injected with QD-JBD1-C_60_ conjugates. Tungsten electrodes are depicted by white lines. (right) Time-resolved Δ*F*/*F*_0_ traces at the various regions of interest from (left, A1–A4) showing the response of QD-JBD1-C_60_ to electrical stimulation as an average of 50 trials. Figure taken with permission from Nag *et al*.^581^ Copyright 2017 American Chemical Society.

Apart from the rather direct strategy to embed the NPs within the membrane to record APs, one can utilize indirect strategies to measure changes in the electric field by monitoring the changes in the energy transfer processes between a donor and an acceptor. Thus, despite the success of inherently voltage-sensitive systems, QD systems that engage in charge and energy transfer have received more attention in the literature, taking the form of QDs decorated with peptides to facilitate interaction with the plasma membrane. For example, the Weiss group realized quasi-type II CdSe-seeded CdS QD nanorods that were directionally assembled with amphipathic peptides containing both hydrophobic, α-helical domains and hydrophilic domains designed to facilitate insertion of the QDs into the plasma membrane bilayer (Figure 20a).^589^ In human embryonic kidney cells, the insertion of QDs into the plasma membrane in various orientations resulted in a voltage sensitivity of the ensemble QDs of ∼0.6% in fluorescence intensity from the baseline (ΔF/F_0_). This voltage sensitivity strongly increased to ∼5% for those QDs exhibiting the optimal membrane bilayer-spanning orientation, demonstrating the tractability of the system and the capacity for further optimization of this approach.

The Delehanty group developed quasi type-II CdSe/CdS/ZnS core/multishell QDs decorated with peptide-fullerene (C_60_) conjugates where the QD and C_60_ engaged in electron transfer (ET) (Figure 20b).^581^ In this QD-peptide-C_60_ bioconjugate, the type-II QD CdSe/CdS/ZnS NPs served as electron donors and the fullerene C_60_ NPs as electron acceptors. The peptide-C_60_ was inserted into the membrane bilayer while the hydrophilic QD decorated the exofacial layer of the membrane. The depolarization of the membrane potential led to enhanced ET from the QD to the fullerene and thus, a quenching of the PL intensity was observed. The rate of ET between the QD and C_60_ tracked the changes in the membrane potential, resulting in the modulation of the QD PL. In HeLa cells, the magnitude of the voltage response tracked inversely with the QD-C_60_ separation distance, with the shortest peptide motif facilitating a ΔF/F_0_ per mV (∼0.4%), equivalent to that of the voltage-sensitive dye (VSD) FluoVolt. In a direct comparison, the designed probe has a 20–40× improvement of the PL intensity changes in the presence of membrane potential in comparison to traditional voltage-sensitive dyes.^581^ Furthermore, this sensing platform demonstrated a ∼ 2% ΔF/F_0_ in an *in vivo* mouse cortical stimulation model, which is a significantly larger response compared to many commonly used VSDs.^590^ The Chen group devised an ET-based voltage imaging scheme using a membrane-tethered CdSe/ZnS QD donor for Förster resonance energy transfer (FRET) with a dipicrylamine (DPA) acceptor.^582^ Glutathione surface ligands of the NP not only provide solubility in the aqueous media but also enable tethering of the NP to the membrane surface *via* electrostatic interactions. Dipicrylamine is a voltage-sensing lipophilic anion, which can translocate from one side of the membrane to the other during depolarization of the cell. In this way, upon depolarization, the DPA migrates to the inner leaflet of the membrane bilayer, resulting in decreased FRET efficiency and increased emission from the QD donor. In the case of hyperpolarization, DPA moves back toward the outer membrane bilayer and the decreased distance between donor and acceptor during this process influences the FRET efficiency and thus results in enhanced PL quenching of the NPs.^582^ Future work on QD-based voltage-sensing systems will focus on meeting several remaining challenges, such as improving QD targeting to neurons of interest, reducing endocytosis of appended QDs, and developing multifunctional QD systems for combined voltage imaging and drug delivery.

While semiconductor NPs can be synthesized routinely, there remain size and shape inhomogeneities resulting from different experimental protocols. Although synthetic approaches have improved, NPs still possess rather broad PL line widths originating from slight differences in the sizes and shapes of the NPs in single batches. Due to only small changes of a few nm for the PL emission maxima in the presence of the electric field, ensemble measurements of many NPs at the same time can mask those changes by averaging PL emission maxima, so-called spectral diffusion. In general, heterogeneity of size, shape, and optoelectronic properties like the PL quantum yield (QY) result in broad distributions of voltage sensitivities and thus limit their sensitivity. This issue might not be a problem for single-particle measurements of immobilized NPs on neurons, but to measure large brain areas with many NPs at the same time, different synthesis approaches need to be developed to improve the material properties.

The potential toxicity of NP materials also needs to be considered. Due to well-established synthesis protocols for the preparation of monodisperse spherical and rod-shaped NPs, cadmium-based NPs are among the most popular nanomaterials used for the investigation of membrane potentials. However, the high intrinsic toxicity of the heavy metal cadmium is a roadblock for any transition of these materials from the lab bench to clinical applications. In this context, the European Union has also severely limited the amount of cadmium in electrical and electronic equipment (RoHS Directive) and added cadmium to the banned list as part of the REACH chemicals legislation.^591^ Therefore, it is imperative that the research community focuses on NP compositions, which have the potential to be useful and applicable in the future. A promising potential candidate for the substitution of Cd-based NPs are indium phosphide (InP) NPs. InP, in turn, with a bulk band gap of 1.8 eV covers the spectral range from the blue to the NIR. InP NPs have already been substituted for Cd-based NPs in commercially available television displays, which shows the promising future of this material. Regarding the potential for voltage sensitivity, theoretical calculations have shown that materials with large effective hole masses are more polarizable. When comparing the effective hole mass of CdSe NPs of 0.45*m*_e_ with InP NPs possessing 0.6*m*_e_, it shows that the larger effective hole mass of InP NPs should result in even higher voltage sensitivity.^580^ Experiments could demonstrate the practical effect on the PL quenching of the large difference in the effective hole masses as InP/ZnS NPs were quenched by 70% in comparison to CdSe/CdS NPs with 60% when exposed to an electric field. Although the local membrane changes in *Xenopus* retinal ganglion cells (RGC) could be measured using InP-based NPs, their lower brightness and fast photobleaching hampered the measurements and showed the need for improved materials engineering.^586^ These examples demonstrate the promising future for InP NPs for neuron activity measurements, while sensitivity against photobleaching and the lack of asymmetric NPs need to be solved to replace Cd-based NPs.

One of the crucial aspects for neuron activity measurements using NPs is the neuron/NP interface. In fact, a major limitation of the utilization of NPs immobilized on a substrate is the inability to fine-tune the distance between neuronal cells and NPs necessary when attempting to record activity. There are requirements on the nanomaterials for reliable membrane potential measurements: (i) no perturbation of the membrane integrity or ion channel activity, (ii) minimal cellular internalization, and (iii) high sensitivity to detect subthreshold voltage changes.^575^ As the electric field dissipates rapidly with the distance from the membrane, the NPs should be as closely attached to the membrane as possible, with insertion providing the best result. Currently, different coatings with lipids and peptides are used to make the NPs biocompatible and to enable the (partial) insertion of NPs in the membrane bilayers. Apart from intimate contact with the membrane, the rod-shaped QDs ideally should be aligned parallel to the membrane, with one end exposed to the cytoplasm and the other to the extracellular matrix.^589,592^ However, the orientation of the NPs’ dipole moment and the electric field of the membrane potential significantly influences the QCSE leading to either blue- or red-shifted PL emission maxima. Lack of orientation control results in the presence of both red and blue shifts at the same time and the effect is then canceled out in ensemble measurements.^578^ By using a α-helical peptide (myristol-CLTCALTCMECTLKCWYKRGCRGCG-COOH), Park *et al*. demonstrated that >60% of the rod-shaped QDs were inserted in small unilamellar vesicles (SUVs) in a favorable orientation. However, when measuring the membrane potential in self-spiking HEK293 cells, the voltage sensitivity was quite poor in comparison to voltage-sensitive dyes. Blinking of the rod-shaped NPs and dynamic movement/orientation in the membrane seemed to be detrimental to their performance.^589^ Similar results were obtained using rod-shaped QDs coated with brain extract lipid mixtures. Although 10% of these rod-shaped QDs showed oriented insertion perpendicular to the membrane plane in SUVs, the analysis of single NPs in single-NP measurements showed that most of the rod-shaped QDs were unresponsive during voltage modulation of cortical neurons. However, the responsive QDs demonstrated PL intensity changes of 5–10%.^592^

While NPs have great potential for neuronal activity measurements and their superior properties over voltage-sensitive dyes are evident, it will be necessary to modify NPs with ligands enabling the specific targeting of subcellular areas of a neuron and enabling better control over the insertion orientation. We must think of ways that not only allow for the insertion into membranes but also to develop transient interfaces that enable automatic removal, *e.g.*, when a brain injury has healed.

### Nanoparticles and Nanomaterials for Transducing Chemical Signals

**Figure 21:**
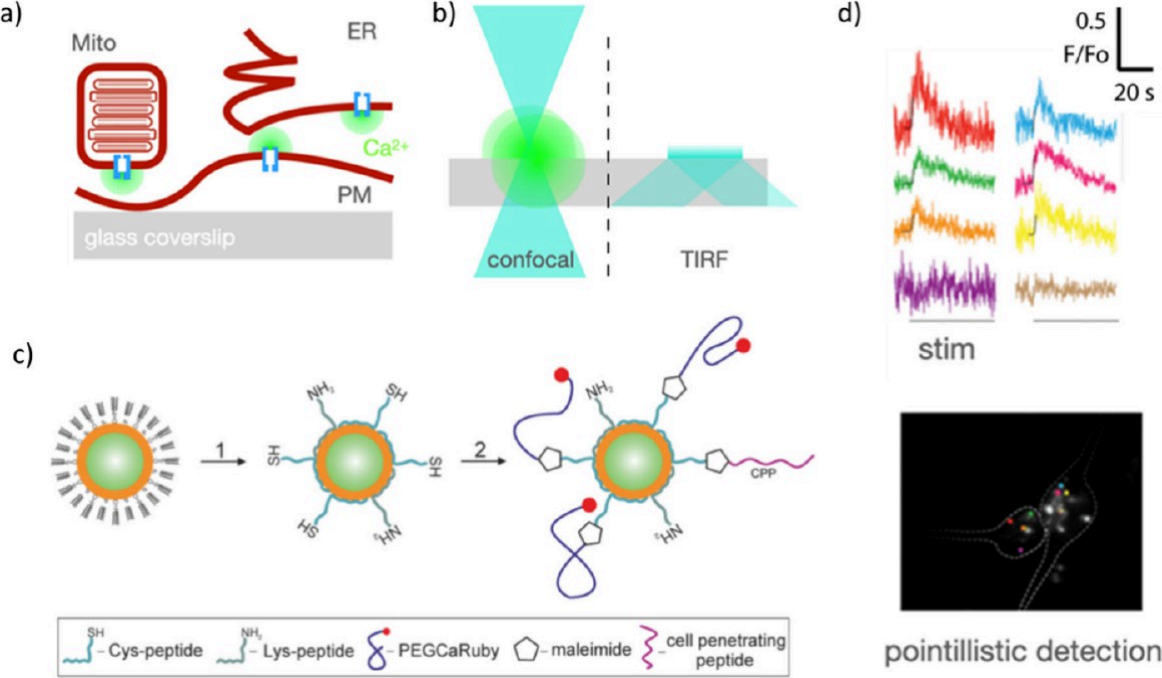
(a) Ca^2+^ channels are located in the plasma membrane (PM), the membrane of the endoplasmatic reticulum (ER), and the membrane of mitochondria (Mito). (b) For observing Ca^2+^*via* Ca^2+^-responsive fluorophores, optical detection is in general performed with confocal microscopy. In the case of imaging fluorophores close to the glass substrate, total internal reflection (TIRF) geometries can be used. (c) Sketch of a nanoparticle (NP) with attached Ca^2+^-responsive fluorophores (PEGCaRuby) and cell-penetrating peptides (CPPs) and (d) corresponding normalized fluorescence signal *F*/*F*_0_ upon stimulation as recorded from the NPs localized in the two cells shown below. Image adopted from Zamaleeva *et al*.^601^ Copyright 2014 American Chemical Society.

Two-photon calcium (Ca^2+^) imaging is commonly used as a proxy for reading out neuronal electrical activity.^593,594^ Not only have Ca^2+^ sensors historically been more sensitive, offering larger dynamic ranges than voltage sensors, the availability of and choice among genetically encoded Ca^2+^ indicators (GECIs) has allowed cellular and subcellular targeting and optical recording from identified cell populations and even defined subcellular locations, long before genetically encoded voltage indicators (GEVIs) were available. Neuronal and astrocytic Ca^2+^ signals span several orders of magnitude, both in the temporal and the spatial domains, with the smallest “elementary” Ca^2+^ signals resulting from the opening of individual Ca^2+^ ion channels located in the cell’s plasma membrane,^595,596^ or in the membranes of intracellular organelles, in the case of ER-Ca^2+^ release channels on endoplasmic or mitochondrial Ca^2+^ stores,^597,598^ see Figure 21a.

The detection of highly localized and short-lived excursions from resting Ca^2+^ presents a major challenge for intracellular Ca^2+^ imaging. High spatiotemporal resolution is required because tiny “microdomain” signals are difficult to detect in front of a bulk background and because cytoplasmically loaded mobile (diffusible) Ca^2+^ indicators act like a “ferry” for Ca^2+^ and accelerate the equilibration of near-membrane Ca^2+^ concentration gradients. Immobile (high-molecular weight or membrane-tethered) Ca^2+^ indicators, the use of low-affinity Ca^2+^ indicators, and optical sectioning techniques such as confocal microscopy or total internal reflection microscopy^599,600^ have been used for increasing the detectability of microdomain Ca^2+^ signals (Figure 21b).

An alternative strategy for confining the fluorescence excitation or readout volume is to label the Ca^2+^ source by targeting fluorescent indicators directly to the molecule of interest. Here, as in single-molecule tracking experiments, a functionalized NP is addressed to the ion channel of interest. Such “pointillistic” Ca^2+^ measurements have been realized by “swiss-knife”-like quantum-dot based nanobiosensors that carried: (i) a cell-penetrating peptide for accessing the cytoplasm, (*ii*) a binding motif for covalently linking some 15–20 Ca^2+^ indicator molecules, and (*iii*) an antibody for targeting the sensor to the precise ion channel of interest, *e.g.*, an *N*-methyl-d-aspartate (NMDA) receptor.^601^ This arrangement enabled low-density labeling and imaging of Ca^2+^ transients resulting from only a few ion channels. Excitation and detection of the (green) NP luminescence allowed for single-particle tracking without appreciable photobleaching (Figure 21c). At the same time, the NP acts as a FRET donor^602^ for the green-absorbing and red-emitting Ca^2+^ indicator molecules decorating its surface. For the red-emitting indicator used as a FRET acceptor, Calcium Ruby, Ca^2+^ binding modulates the fluorescence quantum yield, so that red fluorescence on a green spot indicates the opening of the local ion channel (cluster). Ca^2+^ transients close to NMDA receptors (NMDARs) have been detected in this manner (Figure 21d),^601^ and an analogous approach has been extended for pH measurements^603^ and for caspase4 proteolysis,^604^ but a recurrent problem has been the uneven loading of nearby cells and the overall low throughput.

Classically, the detection of the synaptic release of neurotransmitters has been possible through: (i) the electrophysiological recordings of voltage or current elicited upon the activation of ligand-gated ion-channels located at the postsynaptic terminal^605^ and (ii) the electrochemical detection of neurotransmitters.^606−609^ Electrophysiological recordings are ideal to monitor the release of neurotransmitters from single synaptic vesicles mediating fast synaptic transmission (*e.g.*, glutamate, GABA, and acetylcholine) in single neurons.^605^ Catecholamines (*e.g.*, dopamine, epinephrine, and norepinephrine) and indolamines (*e.g.*, serotonin) are best suited for electrochemical detection through the recordings of the oxidation currents with high temporal resolution.^606−611^ The electrochemical detection of neurotransmitters beyond catecholamines (*e.g.*, acetylcholine or glutamate), requires the participation of specific enzymes to catalyze the oxidative reaction that limits the temporal resolution of the recordings. In the last two decades, advances in chemical synthesis have supported the development of nanosensors to detect different types of neurotransmitters.^612^

**Figure 22:**
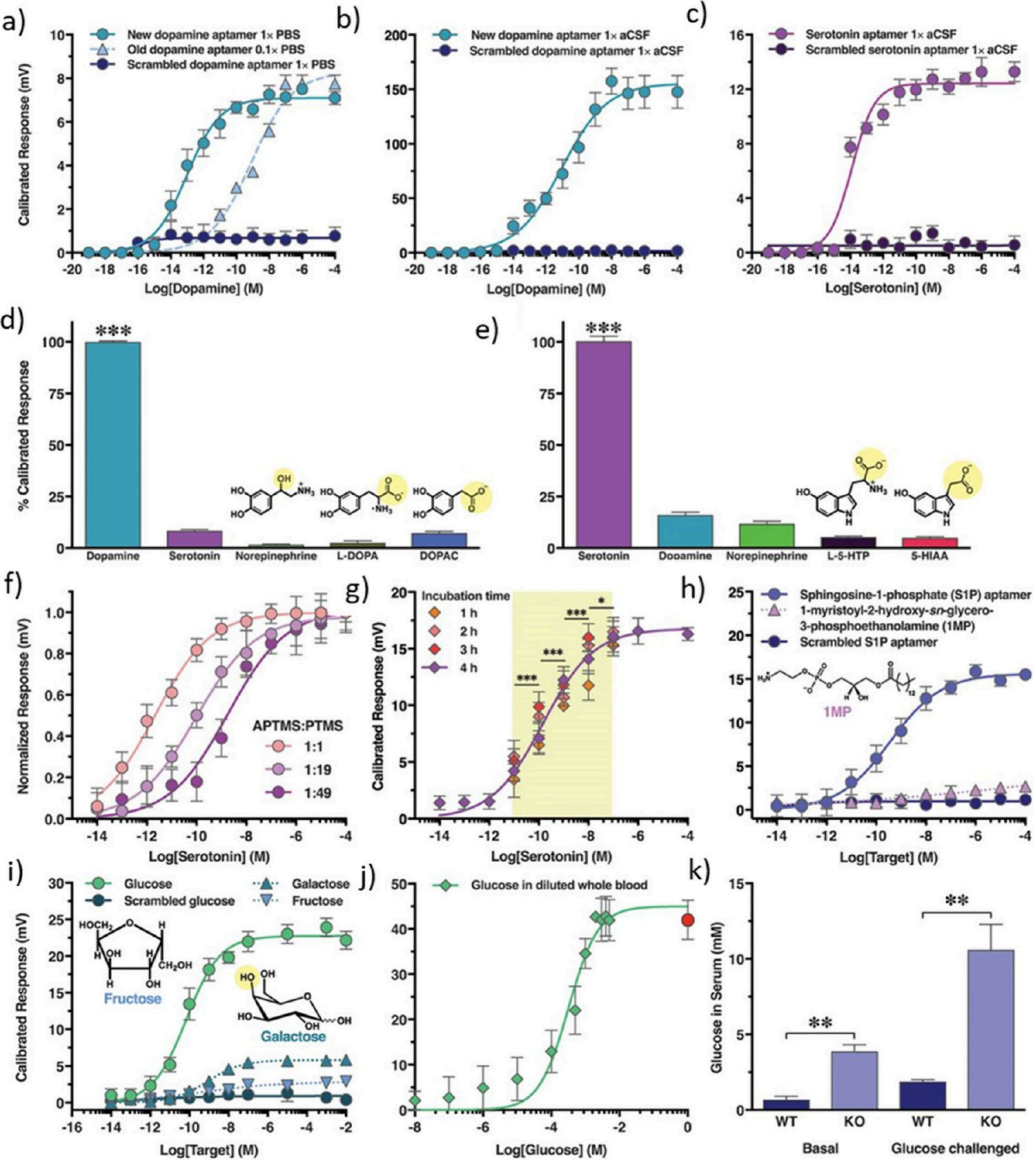
Electronic small-molecule detection using aptamer-functionalized field-effect transistor (FET) sensors. (a) Responses of FET sensors functionalized with a dopamine aptamer (*K*_d_ = 150 nM, 1× PBS) or its scrambled sequence as a control compared to FET responses with a previously known dopamine aptamer (*K*_d_ = 1 μM, 0.1× PBS). (b) The dopamine aptamer–FET and scrambled sequence control responses to dopamine in 1× artificial cerebrospinal fluid (aCSF). (c) For serotonin aptamer–FETs, serotonin in 1× aCSF led to concentration-dependent responses, whereas scrambled serotonin control sequences showed negligible responses. (d) Dopamine aptamer–FET responses to 100 μM norepinephrine, serotonin, l-3,4-dihydroxyphenylalanine (-DOPA), and 3,4-dihydroxyphenylacetic acid (DOPAC) were negligible relative to dopamine (10 nM). (e) Serotonin aptamer–FET responses to 100 μM dopamine, norepinephrine, serotonin biological precursor l-5-hydroxytryptophan (l-5-HTP), or serotonin metabolite 5-hydroxyindoleacetic acid (5-HIAA) were negligible relative to serotonin (10 nM). (f) By altering ratios of amine-terminated/methyl-terminated silanes for surface tethering, serotonin aptamer–FET sensitivity ranges were shifted. (g) Serotonin aptamer–FETs after 1 to 4 h of incubation in serotonin-free brain tissue followed by addition of serotonin had reproducible responses with differentiable physiological concentrations. (h) Sphingosine-1-phosphate (S1P) aptamer–FETs showed concentration-dependent responses to S1P but not to a phospholipid with similar epitopes or a scrambled control sequence in 1× HEPES. (i) In tests of glucose sensing in 1× Ringer’s buffer, the responses of glucose aptamer–FETs were minimal for galactose, fructose, and a scrambled control sequence. (j) Concentration curves for glucose aptamer–FET responses in mouse whole blood diluted in Ringer’s. The red circle shows the response in undiluted whole blood. (k) Glucose aptamer–FETs were able to differentiate hyperglycemia in serotonin transporter–deficient (KO) mice *versus* wild-type (WT) mice by measuring glucose concentrations in diluted serum under basal and glucose-challenged conditions. Error bars are ±SEM with *N* = 6 [(a–c, h, i, and k)] or *N* = 3 samples per group [(d–g and j)]. In (d,e), ***P < 0.001 *versus* countertargets; in (g), ***P < 0.001, *P < 0.05 *versus* different serotonin concentrations (10 pM to 100 nM); in (k), **P < 0.01 KO *versus* WT. Figure used with permission from Nakatsuka *et a*l.^614^ Copyright 2018 The Authors.

Aptamers have distinct advantages in that they can be selected from large libraries to recognize neurotransmitters and other biomarkers and simultaneously *not* recognize (by antiselection) closely related molecules such as precursors and metabolites of the biomolecular targets. Aptamers are most commonly DNA or RNA, but can also be peptide or synthetic nucleic acid sequences. Andrews and co-workers have developed field-effect transistors (FETs) and FET arrays functionalized with DNA aptamers that recognize neurotransmitters with high specificity and selectivity as well as high sensitivity, covering six orders of magnitude concentration over biologically relevant ranges for *in vivo* measurements (Figure 22). Note that the sensitivity of the sensors extends to concentrations well below the *K*_d_ values of the aptamers. The authors attributed this extended range to the logarithmic effect of the transconductance in the transistor. Subsequent measurements in related systems show that on/off rates are rapid, and that the aptamers are repeatedly sampling the local environment.^613^

**Figure 23:**
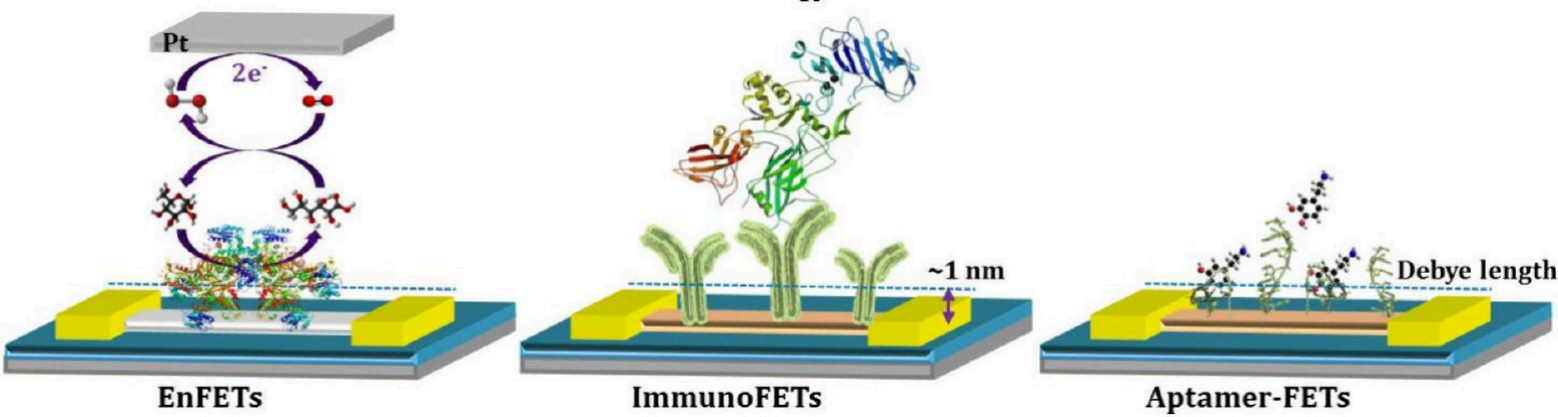
Scales of enzyme-based field-effect transistors (EnFETs) *versus* antibody-based FET sensors (ImmunoFETs) *versus* aptamer-FETs compared to the Debye length *in vivo* over which charge is screened because of the high ionic strength in the brain. EnFETs and ImmunoFETs are more commonly used in the laboratory, where extracted solutions can be diluted and/or desalted. The aptamer-FETs can be used both *in vivo* and *in vitro*.

As the aptamers gate the transistors by moving the charged DNA backbone closer to or further from the surface (Figure 23), a key advance was developing the aptamers in which conformational changes occur close to the surface.^614^ This aspect is critical because the high ionic strength *in vivo* leads to Debye screening lengths on the order of 1 nm. This limit has largely precluded direct *in vivo* electronic measurements using antibodies and enzymes, as these molecules themselves are larger than the Debye length. Stojanović and co-workers have developed methods and quite a number of aptamers for this purpose, including both chemical neurotransmitters and other biomarkers.^615^

Andrews and Weiss have developed nanofabrication methods that combine chemical patterning with chemical lift-off lithography^616^ and sol–gel chemistry to fabricate arrays of these sensors, where 3–4 nm thick In_2_O_3_ functions as the semiconductor.^87,614,617^ These aptamer-FETs have been fabricated on soft, compliant, biocompatible polymer substrates that match the mechanical properties of the brain, an important aspect as discussed earlier.^263^ As in other flexible probe experiments, a carrier is used to place the sensor, and then the carrier shuttle was removed. Zhou and Andrews developed a multiplexed sensor array on a soft poly(ethylene terephthalate) substrate that stayed intact through many bending cycles, had 10 fM detection limits with its dopamine and serotonin sensor elements, and also detected temperature.^618^ In this case, multiple devices were also fabricated in one process. These sensors could be placed on the skin for use as wearables.

**Figure 24:**
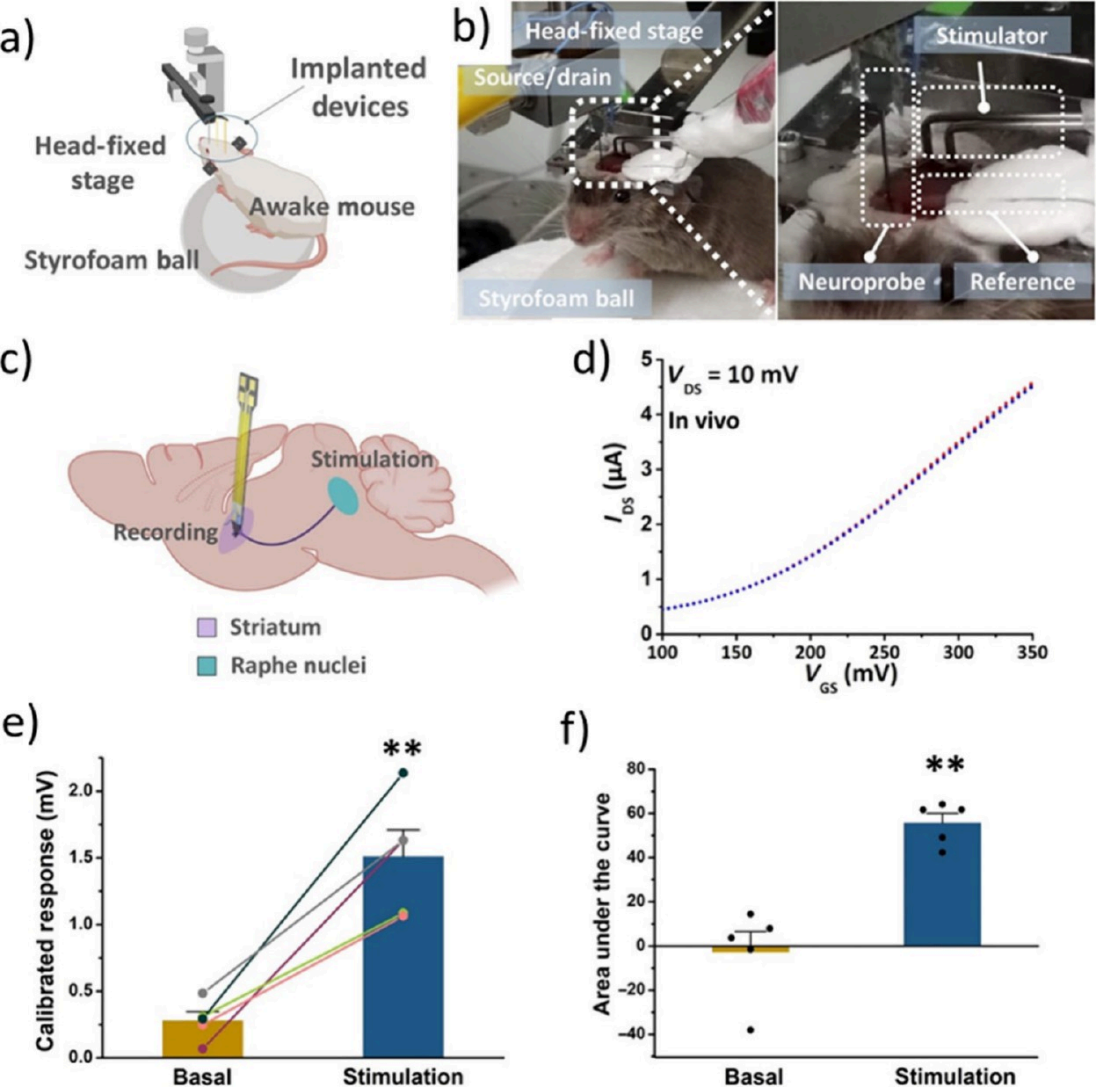
(a) Schematic and (b) digital photographs of an *in vivo* experiment where a neuroprobe, Ag/AgCl reference electrode and stimulator were implanted into the brain of a head-fixed mouse. (c) Schematic of the stimulation and recording sites. The stimulating electrode was implanted into the serotonin cell body region, and the neuroprobe was implanted into a serotonin terminal region in the striatum. (d) Three consecutive overlapping output sweeps *in vivo* where gate-source voltage (*V*_GS_) was swept while source-drain voltage *V*_DS_ was held at constant. (e) Calibrated responses and (f) areas under the curves for *in vivo* measurements of basal and postelectrical stimulation levels from the same mouse, respectively. Error bars in (e) and (f) are standard error of the mean. **P < 0.01 *versus* basal. Used with permission from Zhao *et al*.^617^ Copyright 2021 The Authors.

The aptamer-FETs have been placed into the brains of live, behaving mice and operated with femtomolar to micromolar sensitivity ranges to serotonin for 8 h without significant degradation (Figure 24).^617^ Serotonin release was stimulated elsewhere. First in human studies are planned for the coming months. This same technology has been developed into wearable sensors for measuring biomarkers of stress and other functions.^619^ The FET arrays are also useful for measuring DNA, RNA, and single-base variations and mutations, which target disease diagnostics and genetic analyses, as well as many other biomarkers.^614,620,621^

Nakatsuka *et al*. have leveraged aptamer recognition within nanopipettes with 10 nm apertures for sensing down to the low picomolar range *in vitro*, where aptamer conformation changes upon recognition gate permeability of the pipette orifices.^622^ The nanoscale pore functionalized with aptamers, effectively shields the sensing area from nonspecific protein binding in complex biological environments. These aptamer-modified nanopipettes have enabled quantification of serotonin release from human serotonergic neurons, monitoring of endogenous dopamine release from *ex vivo* brain slices, and measuring of dopamine levels in human serum.

Beyond the requirements of fast temporal resolution to measure neurotransmitter release, nanotechnologies face the challenge of increasing spatial resolution to monitor chemical communication at individual synapses. Genetically encoded fluorescent reporters are powerful tools to study, with advanced microscopy, the dynamic of neuronal communication but they are, however, limited to genetically tractable organisms.^623^ Here, we discuss recent developments of nanosensors to monitor neurotransmitter release with high spatial resolution to monitor neurochemical activity in the brain with nanotechnologies. Highly significant advances have been achieved in the detection of catecholamines, dopamine and norepinephrine, using NIR nanosensors based on functionalized single-walled carbon nanotubes (SWCNTs)^624−627^ and acetylcholine, using DNA-based enzymatic nanosensors.^628^

Carbon nanotubes are formed by multiple parallel concentric graphene cylinders.^629^ Single-walled carbon nanotubes (SWCNTs) are CNTs formed by a single graphene cylinder with interesting properties that depend on the surrounding environment (reviewed in O’Connell *et al*.^630^). In aqueous solutions, SWCNTs emit fluorescence without bleaching in the NIR region (λ ∼ 870–2400 nm).^630,631^ Imaging advantages of NIR include low background and the high penetration into living tissues with low phototoxicity. The high sensitivity of SWCNTs to changes in the environment is a key feature for their use as powerful biosensors. Their sensing properties are tunable through the functionalization of their surfaces by covalent or noncovalent binding of specific molecules, such as DNA, proteins, or synthetic heteropolymers.^632,633^ The selectivity of SWCNT-based sensors is optimized either by screening different configurations of the functionalizing molecules (*e.g.*, different DNA sequences) or by rational design based, for example, on proteins with specific recognition motifs for the analyte of choice (*e.g.*, antibodies). The noncovalent functionalization strategy named as corona-phase molecular recognition (CoPhMoRe)^632−634^ has turned out to be useful in designing neurotransmitter sensors (*e.g.*, dopamine).^634^ CoPhMoRe consists of a heteropolymer that, upon adsorption onto the carbon nanotube surface, forms a structure (corona) that generates a molecular recognition site for the analyte of interest. Since the recognition sites cannot be predicted, the optimal characteristics of the heteropolymer are determined through the screening of chemical libraries.^634−636^

**Figure 25:**
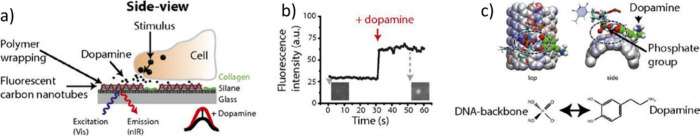
Nanosensor arrays used for chemical imaging: (a) fluorescent single-walled carbon nanotubes (SWCNTs) are made responsive to dopamine by attaching specific single-stranded DNA sequences (ssDNA) to them via noncovalent bonding. These nanotubes are then fixed onto a glass substrate used to culture dopamine-releasing PC12 cells on top. Upon stimulation, PC12 cells release dopamine and the fluorescence of SWCNTs changes. (b) Increase in fluorescence intensity of a single (GA)15-ssDNA/SWCNT (GA = guanine, adenine) induced by dopamine addition (10 μM). (c) The proposed sensing mechanism involves dopamine-pulling phosphate groups toward the SWCNT surface, resulting in the elimination of quenching sites and an enhancement in SWCNT fluorescence quantum yield (molecular dynamics, MD, simulations). Image adopted with permission from Kruss *et al*.^625^ Copyright 2017 National Academy of Sciences.

**Figure 26:**
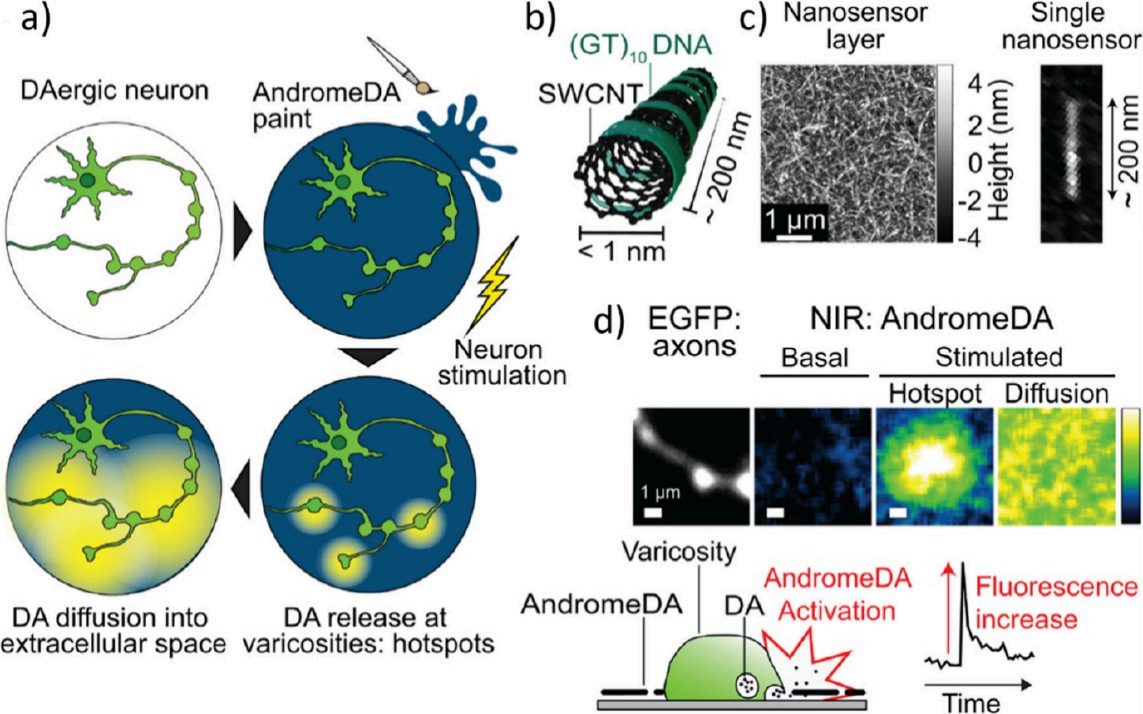
Adsorbed nanosensors detecting release of dopamine (AndromeDA) functions as a dopamine (DA) sensor. (a) A cultured DAergic neuron is coated with AndromeDA paint, which detects DA released upon neuronal stimulation. The interaction of DA with the paint leads to an elevation in nanosensor fluorescence, enabling the detection of the spatiotemporal pattern of DA release and diffusion. (b) Each of the nanosensors utilized in AndromeDA comprises a (5,6)-SWCNT-(GT)_10_ complex (SWCNT = single-walled carbon nanotube, GT = guanine thymine). (c) Left: AndromeDA is composed of a dense layer of individual nanosensors, as visualized through atomic force microscopy (AFM). Right: A magnified view of a single nanosensor is displayed from a lower density nanosensor preparation. (d) Left: A magnified view of an endogenous green fluorescence protein (EGFP)-positive axon with a single varicosity is shown. Right: AndromeDA fluorescence is observed at different time points in the same field of view. Initially, the near-infrared (NIR) fluorescence is low, reflecting the absence of extracellular DA (labeled as Basal). Upon neuronal stimulation, a transient AndromeDA hotspot emerges adjacent to the varicosity (labeled as Hotspot). As DA diffuses, AndromeDA becomes activated over a broader area, leading to a more generalized increase in NIR fluorescence (labeled as Diffusion). Below: A side-view schematic illustrates a DAergic varicosity surrounded by AndromeDA on the glass coverslip (left), and a fluorescence trace (right) illustrates the NIR fluorescence change associated with the hotspot image above it. Taken with permission from Elizarova *et al*.^627^ Copyright 2022 National Academy of Sciences.

Kruss *et al*. used SWCNTs functionalized with single-stranded DNA to generate a dopamine sensor.^625^ Based on this approach, SWCNTs have been used to design (1) a nonphotobleaching fluorescent nanosensor array to map the dopamine release sites in a single secretory cell with high spatial resolution^625^ (Figure 26) and (2) a NIR fluorescent dopamine (DA) nanosensor paint to detect dopamine secretion at varicosities of cultured murine dopaminergic neurons with high spatial and temporal resolution (Figure 25).^627^ Kruss *et al*. have developed a nanosensor array with more than 20,000 sensors to monitor dopamine release with high spatial resolution in one single cell cultured on top of the SWCNTs (Figure 25a). In normal conditions, SWCNTs emit infrared fluorescence that significantly increases upon exposure to dopamine (Figure 25b). In the absence of dopamine, the SWCNTs fluorescence is quenched by ssDNA phosphate groups docked at the SWCNT surface. Apparently, dopamine-mediated increases in fluorescence occur because dopamine attracts phosphate groups to the SWCNT surface, removing quenching sites (Figure 25c).

This approach has turned out to be useful to investigate the relationship between the release sites and features of the cell membrane, such as the local curvature and the directionality of the sequential exocytotic events taking place along the cell surface. In contrast to other neurotransmitters, the organization of dopamine-mediated transmission in space and time has specific features that are now being explored.^637^ Further insight into this phenomenon have been possible thanks to the high spatial and temporal resolution measurements obtained with adsorbed nanosensors detecting release of dopamine (AndromeDA) (see Figure 26).

**Figure 27:**
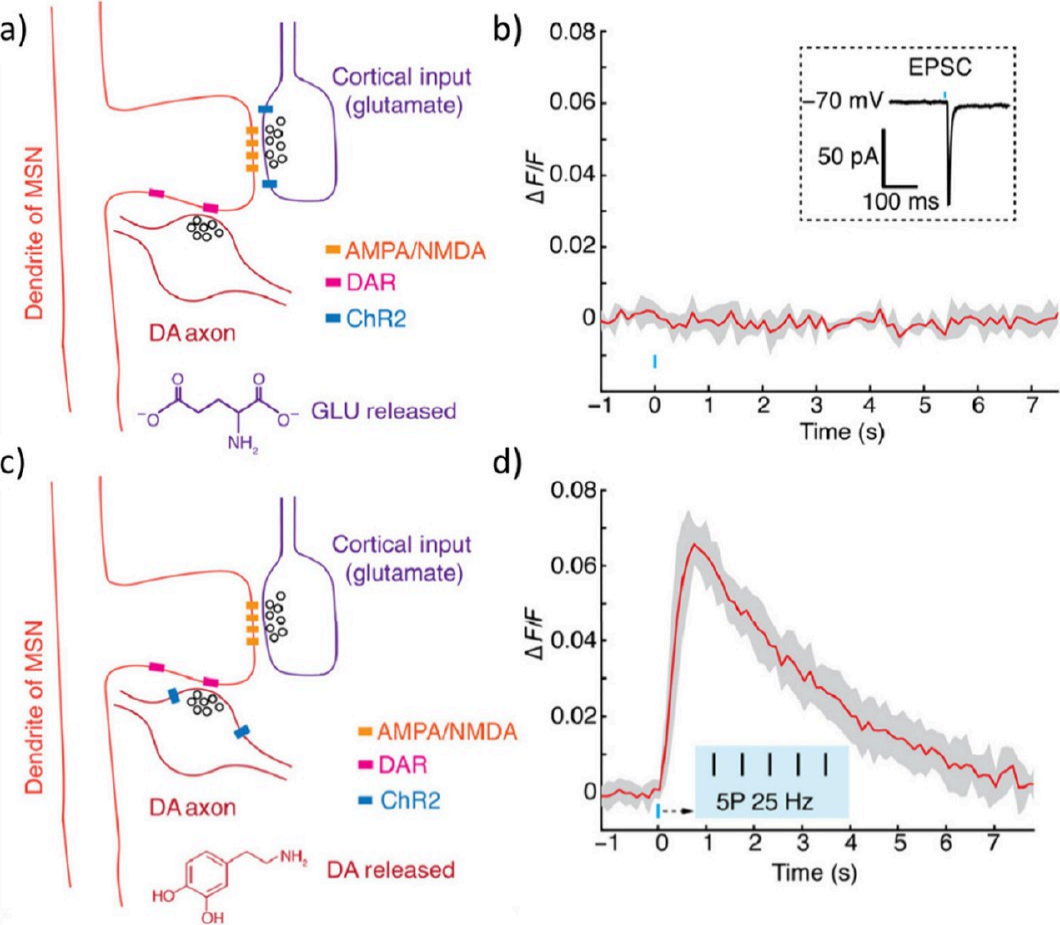
Near-infrared (NIR) catecholamine (nIRCat) sensor method is employed to detect striatal dopamine (DA) release induced by optogenetic stimulation. (a) A schematic illustrates channelrhodopsin-2 (ChR2) expression in cortical glutamatergic terminals forming synaptic contacts in the dorsal striatum. The abbreviations AMPA (α-amino-3-hydroxyl-5-methyl-4-isoxazolepropionate), NMDA (*N*-methyl-d-aspartate), and DAR (DA receptor) are used. (b) Stimulation of glutamatergic terminals did not result in any nIRCat fluorescence modulation. Confirmation of glutamate (GLU) release was achieved through excitatory postsynaptic current (EPSC) recordings on MSN (medium spiny neurons). (c) ChR2 expression is schematically illustrated in nigrostriatal dopaminergic terminals of the dorsal striatum. (d) Stimulation of dopaminergic terminals led to nIRCat fluorescence modulation. The specific stimulation protocol in (b) involved five pulses (5P) at 25 Hz with a power flux of 1 mW/mm^2^, and each pulse lasted for 5 ms. Image reproduced with permission from Beyene *et al*.^626^ Copyright 2019 The Authors.

Primary neuronal cultures require suitable substrates to grow.^625^ Although cell lines such as PC12 grow well on SWCNTs,^625^ neuronal cultures from mouse striatum failed to be maintained directly on SWCNTs. To solve that problem, Elizarova *et al*.^627^ generated a 2D nanosensor layer by applying, immediately before imaging, a concentrated solution of SWCNT-(GT)_10_-ssDNA nanosensors to striatal neurons cultured for several weeks on poly-l-lysine (PLL)–coated coverslips, resulting in the AndromeDA nanosensor on the glass but not on the neurons (Figure 26).^627^ This strategy allowed for the detection of dopamine with spatial resolution high enough to correlate the release of dopamine with subcellular structures called axonal varicosities that store and release the neurotransmitter. Interestingly, the affinity of dopamine for AndromeDA is in the same range as the affinity for dopamine for its receptors. The simultaneous measurement of multiple varicosities in parallel (*ca*. 100 at a time) has revealed interesting properties such as the high functional heterogeneity among varicosities and the fact that most of them do not release dopamine. These are inherent properties of varicosities that likely reflect the heterogeneity of the molecular composition of presynaptic terminals, which are properties suitable to for further analyses.^627^ This approach is sufficiently sensitive for studies of neuronal cultures in which the density of varicosities is low and therefore may be suitable to study human neurons derived from patients such as Parkinson’s disease patients. A future optimization step would be to apply AndromeDA to the analysis of dopamine release in brain tissue in which the densities of varicosities is much higher than in neuronal cultures. Indeed, Beyene *et al*. have used SWCNTs noncovalently functionalized with single-strand (GT)_6_ oligonucleotides to form a NIR catecholamine nanosensor (nIRCat) that is suitable for measuring dopamine release *in situ* in brain slices.^626,638^ Delivery and diffusion of nIRCats into brain tissue occurs through the incubation of brain slices in artificial cerebrospinal fluid (ACSF) containing nIRCats for a few minutes. After rinsing, the slices retain only nIRCats in the extracellular space.^639^ The nIRCats are sensitive to catecholamines, such as noradrenaline and dopamine, but not to other neurotransmitters such as GABA or glutamate. At the dorsal striatum, where the only detectable transmitter with nIRCats is dopamine, Beyene *et al*. monitored dopamine release upon electrical or optogenetical stimulation (see Figure 27) with high spatial resolution monitoring release in small (1 to 15 μm) regions of interest (ROIs).^626^ As expected, in contrast to dopamine, glutamate was not detected (see Figure 27). In the brain of the fruitfly *Drosophila*, dopamine is the only existing catecholamine, so nIRCats can be used without any anatomical restriction to study dopaminergic circuits with subsecond temporal and micrometer spatial resolution.^626^

**Figure 28:**
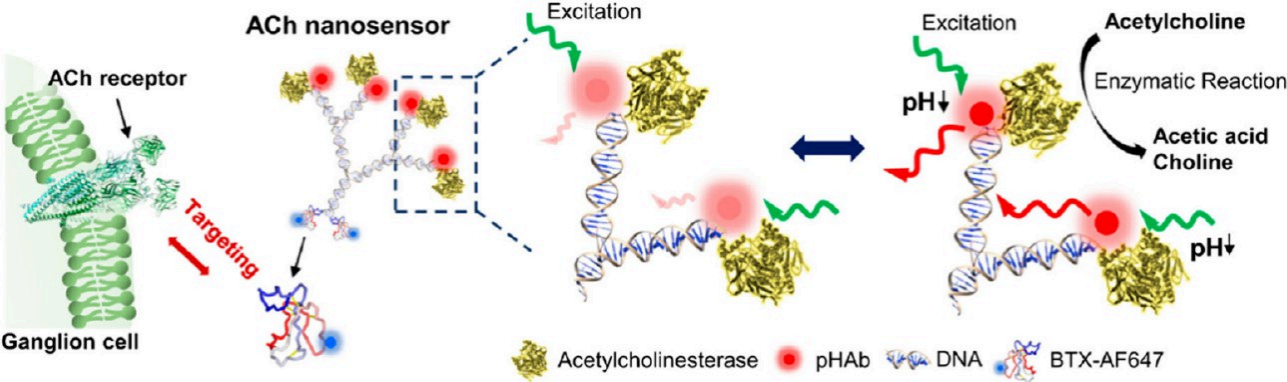
Acetylcholine (ACh) nanosensors’ structure and detection mechanism. The nanosensors are directed to ACh receptors by conjugation with fluorescent alpha-bungarotoxin (BTX). AChE, connected to the DNA scaffold, catalyzes the hydrolysis of ACh, leading to reduction in the local pH due to the production of acetic acid. Four pH-sensitive pHAb fluorophores are located near AChE, causing an increase in fluorescence emission when ACh is hydrolyzed. Alexa fluorophore 647 (AF647) attached to the BTX serves as an internal fluorescence standard, facilitating quantitative measurements. Taken with permission from ref (628). Copyright 2021 National Academy of Sciences.

In addition to aptamer FET- and SWCNT-based sensors to monitor catecholamines, a different approach has been developed to study the synaptic release of acetylcholine (ACh) in the peripheral nervous system of living mice.^628^ ACh nanosensors use double-stranded DNA (dsDNA) as a scaffold, acetylcholinesterase mediates the recognition of the substrate, pH-sensitive fluorophores act as signal generators, and α-bungarotoxin targets the nanosensor to the ACh-receptor (see Figure 28). The dsDNA as a sensor scaffold is efficient and precise to position pH-sensitive fluorophores in close proximity to AChE to detect the decrease in pH produced by the increase of acetic acid associated with ACh hydrolysis at the synaptic cleft (see Figure 28). This method can be used to quantify endogenously released ACh through an *ex vivo* calibration curve.

The overall size (10–20 nm) of ACh nanosensors makes them suitable for *in vivo* delivery through microinjection in ganglia of the peripheral nervous system such as the submandibular ganglion (SMG). The mouse SMG has been used for *in vivo* imaging of presynaptic axonal terminals labeled with genetically encoded fluorescent reporters and postsynaptic sites labeled with fluorescent α-bungarotoxin (BTX).^640^ At the SMG, Xia *et al*. have shown that the electrical stimulation of axonal terminals elicited dose-dependent and reversible responses from the ACh nanosensors, demonstrating the suitability of these nanosensors for monitoring endogenous ACh release within the SMG.^628^ Xia *et al*. have shown that pHAb fluorophores are also compatible with two-photon imaging. In perspective, the versatility of dsDNA scaffold facilitates using ACh nanosensors for brain imaging *in vivo*, especially because pHAb fluorophores are suitable for two-photon imaging, which is the best approach for imaging the activity of neuronal circuits with high-spatial resolution in awake animals.

Beyond fluorescence, other optical signals can be recorded. A combination of light and plasmonic NPs can be used to classify or even to monitor the neuronal behavior, both at the metabolic and functional regimes. Surface-enhanced Raman scattering (SERS)^641^ is a spectroscopic technique that can increase the inherent low Raman sensitivity to molecular vibrations by orders of magnitude. This technique utilizes the strongly enhanced near-fields due to localized surface plasmon resonances (LSPRs) of plasmonic NPs (mainly of gold and/or silver) which lead to a strongly enhanced intensity of the Raman scattering, since the latter scales with the fourth power of the field enhancement factor. SERS can be used in multiple scenarios ranging from the *ex vivo* study of single neurons or neuronal tissues to the *in vivo* quantification of neurochemicals.

**Figure 29:**
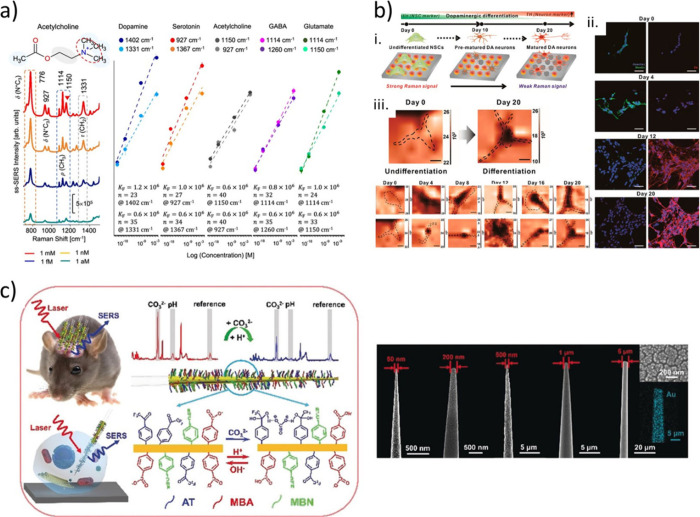
(a) Surface-enhanced Raman spectroscopy (SERS) of acetylcholine at different concentrations and calibration curves for the SERS quantification, down to the attomolar regime, of diverse neurotransmitters. Reproduced with permission from Lee *et al*.^642^ Copyright 2021 The Author(s). (b) Schematic diagram illustrating the method to detect dopamine (DA) release from single live undifferentiated/differentiated neuronal stem cells (NSCs) using graphene oxide (GO)–hybrid SERS. (i) Schematic diagram depicting a strategy to detect DA released from single neuronal stem cells, which were differentiating to neurons for 20 days on a composite consisting of gold nanostructures coated with graphene oxide. (ii) Representative immunofluorescence images of the undifferentiated/differentiated NSCs from day 0 to 20 after induction of differentiation. Scale 50 μm. (iii) Representative SERS images corresponding to (ii) at 830 cm^–1^ (malachite green). The dotted lines indicate the boundary of the cells. Scale bar 5 μm. Reproduced with permission from Choi *et al*.^646^ Copyright 2020 American Chemical Society. (c) The principle of the SERS probe for the simultaneous biosensing of carbonate concentration and pH in live brains and single neurons. Scanning electron microscope (SEM) images of the functionalized gold-coated tips introduced in the cortex of mice. Reproduced with permission from Wang *et al*.^647^ Copyright 2019 Wiley-VCH Verlag GmbH.

The most straightforward application of SERS in the field of neuroscience is the determination of neurotransmitters in external samples (Figure 29a).^642^ This operation can be achieved from a variety of strategies, either by using purified samples containing a single marker^643^ or by coupling the traditional SERS experiment to chemometric techniques for the multiplex quantification of several markers in biological samples.^642^ These determinations can be extended for the quantification of neurotransmitters generated by live neurons on the top of hybrid plasmonic materials. The rationale for these experiments relies on the study of the behavior of these cells as exposed to different physical or chemical triggers. Essentially, these hybrid materials are constituted of a porous top layer of a biocompatible material (*e.g.*, silica, polymers, etc.) that acts as a molecular sieve allowing only the passage of small molecules to the optically active material.^644^ The detection scheme in the optical material can be direct SERS on the plasmonic surface without any further functionalization.^645^ Alternatively, the basic hybrid platform can be modified even to investigate subcellular mechanisms of neurotransmitter secretion.^646^ For example, as shown in Figure 29b, dopamine (DA) released from single live cells has been detected using composites consisting in gold nanostructures coated with graphene oxide. At the same time, the gold structures are functionalized with a dye (malachite green) coupled with an aptamer. As the aptamer presents larger affinity for the for DA than for malachite green, when segregated by the cell, DA displaces malachite green, which results in the decrease of the SERS intensity on the Raman-mapping images (which inversely correlates to the amount of DA).

Finally, plasmonic substrates can be also introduced in the brain to gain information about brain function (Figure 29c). Gold-coated nanotips can be functionalized with three SERS active molecules, 1-(4-aminophenyl)-2,2,2-trifluoroethanone (AT), 4-mercaptobenzoic acid (MBA) and 4-mercaptobenzonitrile (MBN). This molecular mixture generates a complex SERS spectrum, which is sensitive to carbonate concentration (due to AT) and pH (due to MBA). MBN is used as an internal control. The tip can be then introduced into the cortex to monitor the variation of carbonates and pH in a dynamic regime upon illumination with a NIR laser.^647^

### Traversing the Blood–Brain Barrier

There are many opportunities for NPs and nanomaterials to serve as signal transducers. However, apart from the functionality of the signal transducer, its positioning is essential. Delivery of nanomaterials to the brain is not trivial and is a major obstacle. The most straightforward way is to use surgery,^648^ thereby circumventing the need to cross the BBB. Nanostructured devices can be implanted into the brain or the spine by surgery or *via* spinal needle punctuation. Sometimes, the treatable target is located inside the blood circulation system of the brain, as in the case of stroke, and is therefore accessible to NP-based therapies.^649^ If that is not the case, *e.g.*, as the target cells are diffusely distributed in the whole brain, NPs could be applied intravenously. The first challenge is the avoidance of the blood clearance by macrophages^650^ as then the NPs would end up in the liver or spleen. Avoiding clearance has been demonstrated by surface coatings, *e.g.*, with PEG or functional proteins.^651−654^ The biggest hurdle is the undisturbed BBB. As the brain needs to be protected from unwanted molecules, which would interact with neurons or other cells in the brain, endothelial cells serve as the main gatekeepers and form a tight and highly selective barrier to molecules.^655^ They form especially tight connections between themselves (tight junctions) in order to prevent paracellular diffusion. Nevertheless, specific proteins of the tight junctions like the protein claudin-1 can be targeted to enable the paracellular transport.^656^ The BBB also controls the active transport of small molecules by specific transmembrane transporters through the endothelial cells, even with the possibility to “pump out” unwanted small molecules by multidrug resistance pathways, *e.g.*, by P-gp.^657^ Macromolecules or NPs can be transported by transcytosis or receptor-mediated transcytosis (*e.g.*, insulin^658^ or transferrin^659,660^). Other cells involved are the pericytes and microglial cells, which determine what molecules and NPs can enter the brain or get in contact with other cells inside the brain. For inflammatory processes, in situations where gross damage has occurred, such as after stroke or when a tumor is growing, the BBB can be less tight and even NPs can then nonspecifically enter the brain.^661^ Particle size plays a role; ultrasmall gold NPs have been shown to cross the BBB in a 3D brain organoid model.^662,663^

Poly(butylcyanoacrylate) (PBCA) NPs have been found to cross the BBB^664,665^ by the effects of the polymers themselves or by the surfactants (mostly polysorbate-80) used. The specific adsorption of ApoE, B100, or ApoA1 on these NPs was found to be crucial.^666^ Other materials traditionally used by groups coming from the pharmaceutical field are poly(lactic-*co*-glycolic acid) and poly(lactic acid).^667^ Note that when traversing the BBB, adsorbed protein compositions change considerably.^668^

While controlled crossing the BBB by NPs has not been solved, the above examples show that there are strategies that have potential. There are several methods known to open the BBB nonspecifically with chemical or physical stimuli.^669−673^ Heating by localized IR illumination up to 42 °C for less than 10 min^674^ led to a spatially confined opening, as did confined superparamagnetic iron oxide nanoparticle (SPION) heating in the arbitrarily positioned field-free spot of a magnetic particle imaging (MPI) system.^675^ Thus, crossing the BBB is not the ultimate hurdle and in fact, many interfacing applications with NPs used as transducers need to be located selectively in different regions of the brain, at specific cells, and even at specific ions channels, which appears feasible by different means.

### Challenge of Specific Interfacing

To make any NP probe work in the intact brain, it is better to decorate only specific subsets of neurons with NPs. While antibodies against extracellular epitopes work well in cell culture systems, this strategy may not be sufficiently selective for *in vivo* applications. In some cases, one would like to measure signals only from neurons that project to a specific brain region. A major advantage of specific sensor targeting is that the readout does not require any spatial resolution. Fiber photometry can then be used to record signals simultaneously from several deep brain regions in behaving animals.^676^ To reach this level of specificity, we envision hybrid targeting systems in which a genetically encoded designer ligand is selectively expressed on the surfaces of specific neurons, *e.g.*, by retrograde transsynaptic labeling.^677^ The NPs would then be conjugated to molecules with high affinity binding, effectively labeling the genetically modified neurons of interest.^678^ Other strategies for increased specificity include dual receptor targeting.^679^ These considerations also apply to the use of NPs for neuronal stimulation: structuring light in 3D is difficult in scattering tissue, although several holographic methods are under development.^680^ If NPs are made to adhere to selected populations of neurons, diffuse illumination would still generate specific and well-defined effects. For both fundamental neuroscience research and medical applications, hybrid interfacing strategies with a genetic component could be the key to success and widespread application of NPs.

## Advanced Test Platforms to Model Aspects of the Brain

Cell culture systems play important roles in developing strategies for neuronal stimulation, as experiments in live animals require special approvals and have to be kept to a minimum (3Rs principle).^681^ Neuronal cell and tissue culture systems offer many experimental advantages: the level of neuronal activity is easily controlled by the constituents of the recording medium, electrophysiological or optical recording of neuronal activity is straightforward, the intensity of light or electromagnetic fields can be precisely calibrated to generate dose–response curves, and genetic manipulation of individual neurons is possible. Rather than listing the many cell culture systems that have been developed, we will highlight a few approaches that we consider particularly useful for evaluating neuronal interfaces. Such interfaces are not meant to replace *in vivo* experiments, but rather to supplement them when higher throughput recordings are needed.

### Organotypic Slice Culture of Rodent Hippocampus

As a step toward applications in live animals, using thin slices (0.3–0.4 mm) of brain tissue is an attractive way to test the efficacy of new stimulation methods in the presence of all relevant cell types (neurons, astrocytes, microglia). Brain slices can be used immediately after the cutting process (acute slices), or maintained in a tissue culture incubator for several weeks (organotypic slice cultures). Acute brain slices are typically used when genetic manipulation of neurons is not necessary (*e.g.*, dye- or NP-based strategies) and only short-term effects (<3 h) are to be investigated. Alternatively, slices can be prepared from the brains of transgenic animals expressing optogenetic actuators or reporters, *e.g.*, genetically encoded calcium (Ca^2+^)^682^ or voltage^683,684^ or neurotransmitter sensors.^685,686^ For strategies involving the transient or acute expression of modified proteins (optogenetic, chemogenetic, thermogenetic, magnetogenetic), long-term culture systems are required for transfection and expression of the genetic component. Slices of rodent brain tissue can be cultured for many weeks under sterile conditions.^687^ To maintain the appropriate pH of the cell culture medium, cultures are grown in incubators with 5% CO_2_, 37 °C, and high humidity on tissue culture inserts with semipermeable membranes (*e.g.*, Millipore, Nunc). Slice cultures are maintained at the interface between the culture medium and the humidified incubator air^688^ for optimal gas exchange (O_2_/CO_2_).

**Figure 30:**
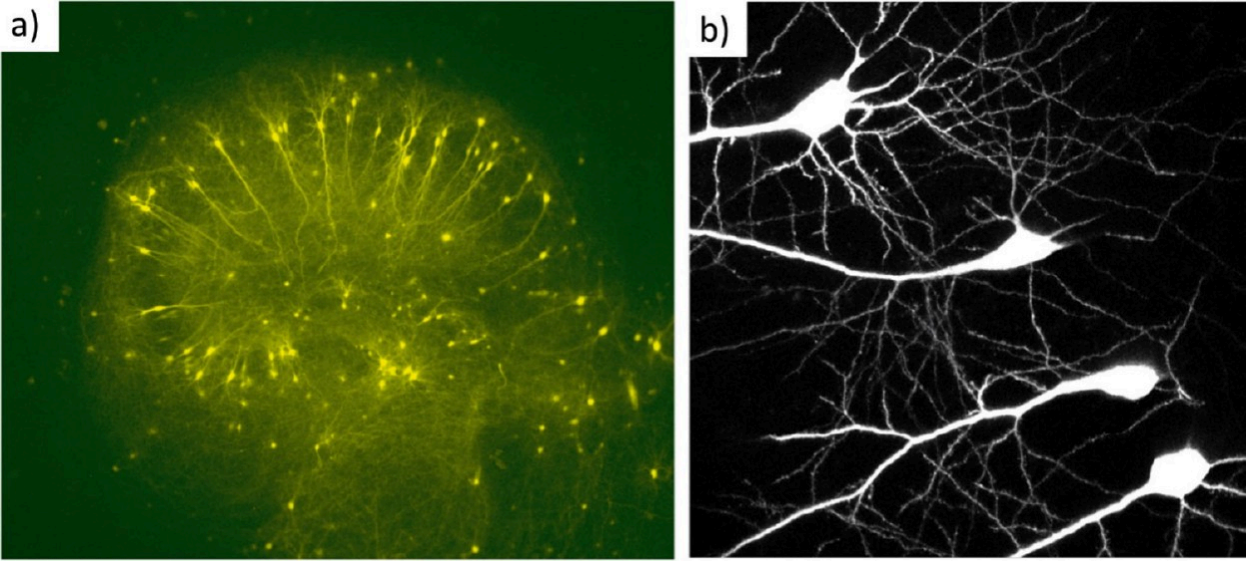
Organotypic slice culture of rat hippocampus. (a) Result of gene gun transfection with endogenous green fluorescence protein (EGFP). No scale bars available. Adapted with permission from Holbro *et al*.^694^ Copyright 2009 National Academy of Sciences. (b) Single-cell electroporation of CA1 pyramidal cells with a genetically encoded calcium sensor. No scale bars available. Adopted with permission from Wiegert *et al*.^695^ Copyright 2013 National Academy of Sciences.

Hippocampal slice cultures establish a pattern of connectivity that resembles the cytoarchitecture *in vivo*, including synaptic connections.^689^ The different neuronal circuits of the hippocampus (CA1, CA2, CA3, DG) are maintained and can be readily identified under the microscope. Organotypic cultures have proven to be useful testbeds for the characterization of optogenetic tools^690,691^ and NPs.^692^ To transfect a random set of neurons with the construct of choice, biolistic transfection (*e.g.*, gene gun, Figure 30) or viral transduction can be used. Single-cell electroporation allows for targeted transfection of a defined set of neurons (Figure 30). Combining good optical and electrophysiological accessibility with a lifetime of several weeks, both acute effects of stimulation and potential long-term effects on cell health and network connectivity can be studied in organotypic slice cultures.^693^

**Figure 31:**
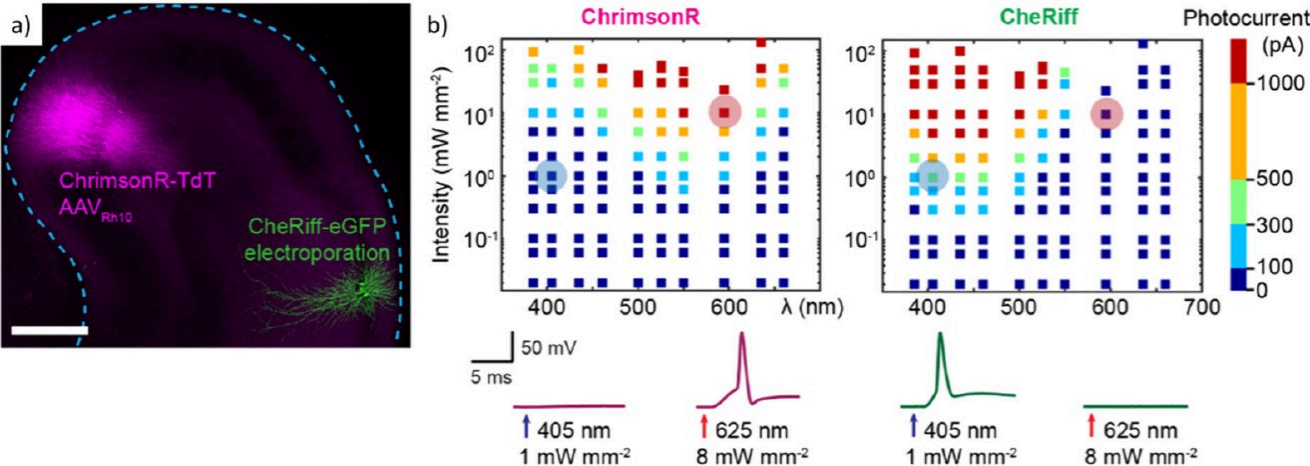
Time-controlled spiking of two neuronal populations in organotypic culture. (a) Hippocampal slice culture with CA3 neurons expressing ChrimsonR (magenta) and CA1 neurons expressing CheRiff (green). Scale bar: 500 μm. (b) Photocurrent amplitudes measured at different wavelengths and intensities (1 ms light pulses). Typical responses to 405 and 625 nm light pulses (current clamp) are plotted below. Figure adopted from Anisimova *et al*.^696^ Copyright 2022 The Author(s).

Full optical access from both sides of the organotypic culture allows for computer-controlled illumination patterns inside the incubator with precisely calibrated intensity and timing (see Figure 31). Activation of different channelrhodopsins in specific neuronal populations was used to investigate spike-timing-dependent plasticity.^696^ This form of synaptic plasticity was discovered by electrode-based spike induction in dissociated neuronal culture,^697^ with a total recording time of 20–30 min. Optogenetic stimulation in organotypic culture allows following the fate of potentiated synapses for several days, revealing different underlying mechanisms for learning. Effective optical access is also desirable for optical recording or photoconversion experiments.^698^ An additional layer of genetic control can be introduced by using specific driver lines of mice for the production of organotypic slice cultures. For example, a mouse that expresses Cre recombinase specifically in microglia can be used to restrict expression of a channelrhodopsin to this particular cell type.^699^ Diverse transfection methods, defined neuronal and glia populations, and excellent availability of this model make the organotypic slice culture an ideal test bed for the development of neuronal interface approaches before experiments on live animals are considered.

Note that it is not straightforward to jump the species barrier and to utilize organotypic brain slices from human tissue due to ethical aspects and availability issues. However, Ravi *et al*. established human cortex OTC from mandatory resected tumor access tissue under strict ethical supervision and showed electrophysiological activity for several weeks in the context of research on neuro-oncological tissue microenvironment.^36,700,701^

### 3D Organoids as *in Vitro* Brain Models

For screening approaches and also for minimizing reliance on studies involving live animals, 3D cell cultures are highly useful. In the past decade, cell culture models have been developed to study the effects of drugs or NPs on the brain. Although these models do not fully mimic the complex structures and signal transduction pathways in living organisms, they have reached a stage where fundamental insight can be gained without the need for animal experiments. Thus, they form an important bridge between classical 2D cell cultures (which represent rather simplified models) and *in vivo* systems, *e.g.*, living animals or humans. As results from small animals, such as mice, are not always consistent with human results, an exciting perspective is to grow organoids from reprogrammed skin cells of human patients (induced pluripotent stem cells, iPSC) to test possible treatments directly. An alternative is to use primary human cells in organoids.^702^

Such organoid models have been developed for a number of organs.^703^ Typically, they consist of a 3D arrangement of tissue-specific cells, either immortalized cell lines or (preferably) primary cells. The complex structure of an organ is mimicked by using a number of cells that need to be arranged similarly to their original structure. Brain organoids consist of different cell types that, after suitable preparation steps, self-arrange in a spherical structure, where neural cells reside at the core. Toward the surface, endothelial cells form so-called tight-junctions that act as BBB, preventing the penetration of drugs or NPs from the outside.^704−707^ For six-cell organoids, astrocytes, pericytes, endothelial cells, microglia, oligodendrocytes, and neurons have been employed.^663,702^ Such 3D organoids can act as *in vitro* models to test the ability of drugs, salts, and NPs to cross the BBB.^702,703,706−711^ Besides assessment for toxic effects of exogeneous compounds, they have potential in devising and testing strategies against neurological conditions, including brain tumors, neurodegenerative diseases, and stroke.^39,706,712−715^

The structure and function of a brain organoid have to be validated to ensure that the *in vitro* results correspond to living systems. The internal arrangements of the constituent cells can be analyzed by two-photon^716^ or confocal microscopy after staining the different cell types (CellTracker and similar compounds).^702^ The integrity of the BBB can be evaluated by transepithelial electrical resistance (TEER) and biomolecular transport methods. The application of histamine^717^ or mannitol^718^ can increase BBB permeability *in vivo* and also *in vitro*. Electronics can be efficiently interfaced at the surface of the organoid or synergistically develop within the 3D cellular structures for electrophysiology studies.^719−722^

**Figure 32:**
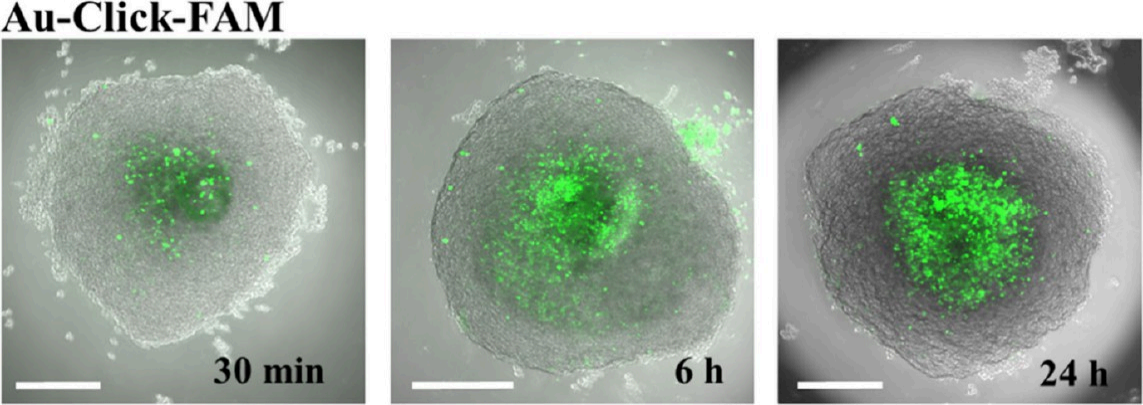
Uptake of ultrasmall carboxyfluorescein (FAM)-labeled gold nanoparticles (Au-Click-FAM; green fluorescence) and dissolved FAM-alkyne by six-cell brain organoids over 30 min, 6 h, and 24 h. Scale bars 200 μm. Reproduced with permission from Sokolova *et al*.^663^ Copyright 2020 The Author(s).

Although there are conflicting reports as to whether NPs are able to penetrate the BBB, it is generally difficult for NPs to reach deeper brain tissue, especially if they are larger than a few nanometers (see Figure 32).^662,711,723−725^ This limit constrains nanomedical approaches where NPs are used for tumor imaging and treatment, drug delivery, to name just a few applications, and using NPs as signal transducers as discussed in the section “Colloidal Nanoparticles as Transducers”.^726−728^ Suitable surface treatments can facilitate NPs crossing the BBB.^729^ For magnetic resonance tomography (MRT) imaging with magnetic iron oxide NPs, damaged regions of the brain vascular system, *e.g.*, around a tumor, can be more easily penetrated by NPs, and exploited to localize NPs in brain tumors. Ultrasmall gold NPs (∼2 nm) penetrate the BBB in an *in vitro* organoid model with high efficiency.^662,663^ Sokolova *et al*. demonstrated the transport of small molecules across the BBB that are not readily able to cross it in dissolved form. This result underscores the potential for NPs to target the brain, provided that they are sufficiently small and possess a suitable surface chemistry.

### Brain-on-a-Chip to Model Information Exchange between Brain Regions

In neuroscience, understanding the intricate communication between different brain regions is crucial for unraveling the complexities of normal brain function and identifying potential abnormalities associated with psychopathologies. There has been increasing recognition of psychopathological states as brain disorders, providing a foundation for neuroscience research to advance clinical practices in psychopathology.^730^ However, translating basic knowledge about the neural mechanisms underlying psychiatric conditions into practical clinical applications poses challenges.^730^ Thus, there is a strong need to develop models for study that can faithfully reproduce the features of information processing in healthy brains and also in those with psychopathologies. Traditional methods, such as *in vitro* culture of single brain structures or *in vivo* animal models, are limited in accurately reproducing the complex interactions between multiple brain regions at physiologically relevant spatiotemporal resolution. However, as discussed in the section “Improving Cell-Electrode interfaces: Materials and Coatings”, there are ongoing efforts for interfacing different brain regions, see for example Figure 9.^221^

*In vivo* studies have already revealed correlations in activity between neocortical and hippocampal neurons from these two brain regions.^731^ However, there is not yet a complete connectivity map, partly due to the complexity of connections between different brain regions. An unbalanced ratio between excitation and inhibition in neuronal activity is critical in schizophrenia-like cognitive dysfunctions, particularly in two brain structures fundamental for cognitive processing: the prefrontal cortex (PFC) and the hippocampus (HPC).^732^ Coordination between different cellular assemblies in PFC and HPC is orchestrated by coordinated excitation/inhibition (E/I) of neurons. It has been hypothesized that pathological thought processes are due to improper associations between stimuli. When two or more cell assemblies are uncoordinated between brain regions, they result in loss of outgoing information.^733^ Optogenetic stimulation of NMDA receptor/parvalbumin-positive (PV+) interneurons putatively rescue the excitatory/inhibitory balance, recovering cognitive abilities.^732^ By replicating the communication patterns between brain regions, such as the PFC and the HPC, researchers could elucidate how abnormalities in brain communication contribute to the development and manifestation of psychopathologies.^734,735^

As outlined here, microfluidics-enabled platforms can emulate neural tissue with BoC devices.^736,737^ These models are powerful tools to replicate and to study the coordinated activity among various brain regions by integrating different brain areas within one chip, enabling systematic investigation of the information exchange.

Thus, BoC models have great potential for providing understanding of brain communication and its role in psychopathology, by creating models that can provide insight into the intricate mechanisms underlying neurological diseases such as schizophrenia.^738^ By integrating different brain regions in controlled environments in BoC models, coordinated activity among various regions is replicated for study. Considering the modular structure of the brain, different attempts have been made to recreate *in vitro* interconnected neuronal networks using geometrical constraints and wells in the microfluidic chips. These microfluidic wells mimic a brain region’s physiological specificity to contain specific cell types.^739^ Following this method, individual cells can be grown on BoC chips, either *ex-vivo* or derived from stem cells.

One approach to developing BoC models to study brain communication consists of using 2D platforms with well-defined geometries that create compartmentalized areas with controlled environments, to coculture different subtypes of neuronal cells that self-organize into microstructures.^740^ Then, microfluidic structures are used to control and to guide the growth of neuronal networks through geometrical and surface cues, sometimes combined with chemical cues.^111,134,741,742^ One interesting proof-of-concept was shown by Kamudzandu,^739^ where the authors built a five-port microfluidic device, intended to mimic a complex neuronal circuit with neurons from five different brain regions (cortex, striatum, globus pallidal, and substantia nigra compacta and reticulata). Each port was seeded by primary neurons specifically isolated from the various brain regions, and axonal outgrowth was directed from one port to an adjacent port *via* tapered microchannels. The neurons extended their axons from the cortical port into the striatal port, similar to their activity *in vivo*. Functional connectivity of the neuronal circuitry was studied using calcium imaging, and the observed activity mimicked that expected from *in vivo* circuitry. This work aimed at studying neurodegenerative diseases and potential treatments, but the general approach can be extended to other BoC models. For example, these 2D BoC models can reproduce and compare the features of communication between neuronal assemblies, in a healthy brain *versus* in one with psychopathologies.^736^ Other studies focused on engineering brain regions such as the cortical-striatal^743^ or the cortical-hippocampal-amygdala.^744^ There are reports of modeling neuronal disorders using *in vitro* compartmentalized microfluidic devices. For example, Taylor *et al*. used a design with two separated compartments connected with microchannels, where only neural processes but not cell bodies can enter the channels.^745^ They reported that axons from cortical and hippocampal neurons spontaneously traverse the microchannels without applying neurotrophic factors during a two-week experimental window.

In addition to cellular compartmentalization, microfluidic chips can be further integrated with electromechanical actuators,^746^ electrodes,^747^ and optical readout,^748^ which can then be used for real-time excitation, monitoring, and detection of neuronal activity even at the single cell level.^152,747,749^ Combined with optogenetics (see section “Colloidal Nanoparticles as Transducers for Communication with Different Ion Channels or Neurotransmitters”) and chemogenetics, integrated microfluidic chips and BoC platforms offer powerful tools for simulating and analyzing cell-to-cell signaling in real time and under controlled conditions. We anticipate that these platforms will be used for therapeutic compound discovery to treat neurological disorders. In addition, personalized immunotherapies for cancer treatment could be developed by extracting multi- or pluripotent cells from the patient and differentiating such cells into specific (neuronal) cells, directly in the BoCs, and optogenetically modifying them to express the appropriate membrane proteins (in the opsin family). Furthermore, developing physiologically relevant reconstructions of the BBB integrated within BoC devices would enable alternative possibilities for studying drug-delivery mechanisms and evaluating neurotoxicity.^750^

Overall, using BoCs represents a methodology to control the temporal and spatial behavior of cells and cell assemblies with high fidelity. They represent a practical example of methodologies aligned with the 3R’s principle (Replace, Reduce, Refine). Thus, with ethics and the lack of models in mind, BoC technologies can bring us closer to understanding the neurophysiological activity and the neurological disorders connected to it. We note important limitations of BoCs, such as not fully recapitulating systemic effects on neural tissue; therefore, the scientific questions pursued with these platforms need to be carefully considered to prevent artifactual results.

### Nano- and Microfluidics with Tailored Porous Materials for Brain Interfacing

Nanostructured materials in combination with nano- and microfluidics have made significant impact on the development of multifunctional electrodes for neurological applications.^751−753^ For example, nanoporous gold electrodes, produced by a microfabrication-compatible dealloying-based self-assembly process,^754^ reduce the electrical impedance of recording electrodes due to increased effective surface area, which diminishes the baseline noise in extracellular recordings.^755^ The nanoporous matrix also sustains electrochemical functionality in biofouling conditions (*e.g.*, fetal bovine serum) *via* a size exclusion mechanism, where large biomolecules cannot permeate the electrode, yet ionic transport in/out of the pores persists,^756,757^ thereby maintaining the low electrode impedance over chronic recordings. Finally, nanoporous electrodes can decrease astrocyte spreading while maintaining neuronal attachment *via* topographical cues, which improves the transduction efficiency in capturing ionic signals from the neurons.^758^

Titanium nitride (TiN)^759,760^ and iridium oxide (IrOx)^761,762^ have been well established as materials for neuroelectronic interfacing. Both materials offer low-impedance interfaces. In addition, IrOx is one of the best materials for neuronal stimulation due to its large charge injection capacity. Some of these features described above, especially the large effective surface areas, supplemented with conformal coatings (*e.g.*, TiN, IrO_*x*_) on the nanoporous Au electrodes increase charge injection capacity, which is critical for safe electrical stimulation of neuronal tissue at low potentials.^763,764^ Use of other noble metals, such as platinum, has also shown improvements in recording and stimulation performance.^765^

In tandem, the high effective surface area allows for small molecule retention and release from the porous electrodes with physiological relevance evidenced by reduced astrocyte proliferation on nanoporous Au electrodes loaded with an antimitotic small-molecule drug (cytosine arabinoside).^766^ Small molecules (*e.g.*, fluorescein) can also be ionophoretically released *via* modulating the surface charge, enabling time-varying dosing.^346,767^ In particular, polymer-based materials have been utilized for the fabrication of organic electronic ion pumps (OEIP), which can actively administer chemicals locally without microfluidic actuation.^768−770^ Graphene can also be utilized as active material of for OEIPs.^771^ To power OEIP devices, combining them with photovoltaic devices has been proposed.^772^ Other studies have demonstrated that fluid transport can be controlled *via* modulating surface wettability by varying the surface charge of nanoporous gold fibers,^773^ and small molecules can be transported large distances along thin nanoporous gold traces,^774^ enabling paths for drug delivery and replenishment of drug depots.

The biocompatible and biodegradable characteristics of nanostructured porous silicon and porous silicon hybrid materials have been attracting increasing attention for applications at the nanoneuro interface.^151,775−779^ The unusual mechanical,^569,574,780^ optical,^781,782^ optofluidic,^783^ and electrical properties, as well as their complex multiphysical couplings^573^ along with the biochemical-stimuli sensitive biodegradation^777^ and the plethora of routes to control the structure, size, and nanoporosity enables the realization of a diverse set of static and (fluid-) dynamic biofunctionalities into porous silicon.^776,784^ Increasingly better fundamental understanding of nanoconfinement effects on thermal equilibrium and nonequilibrium transport properties of molecular systems in nanoporous media^785,786^ has contributed to the rational design of porous silicon applications in physiological environments, in particular at the brain interface.^776^

**Figure 33:**
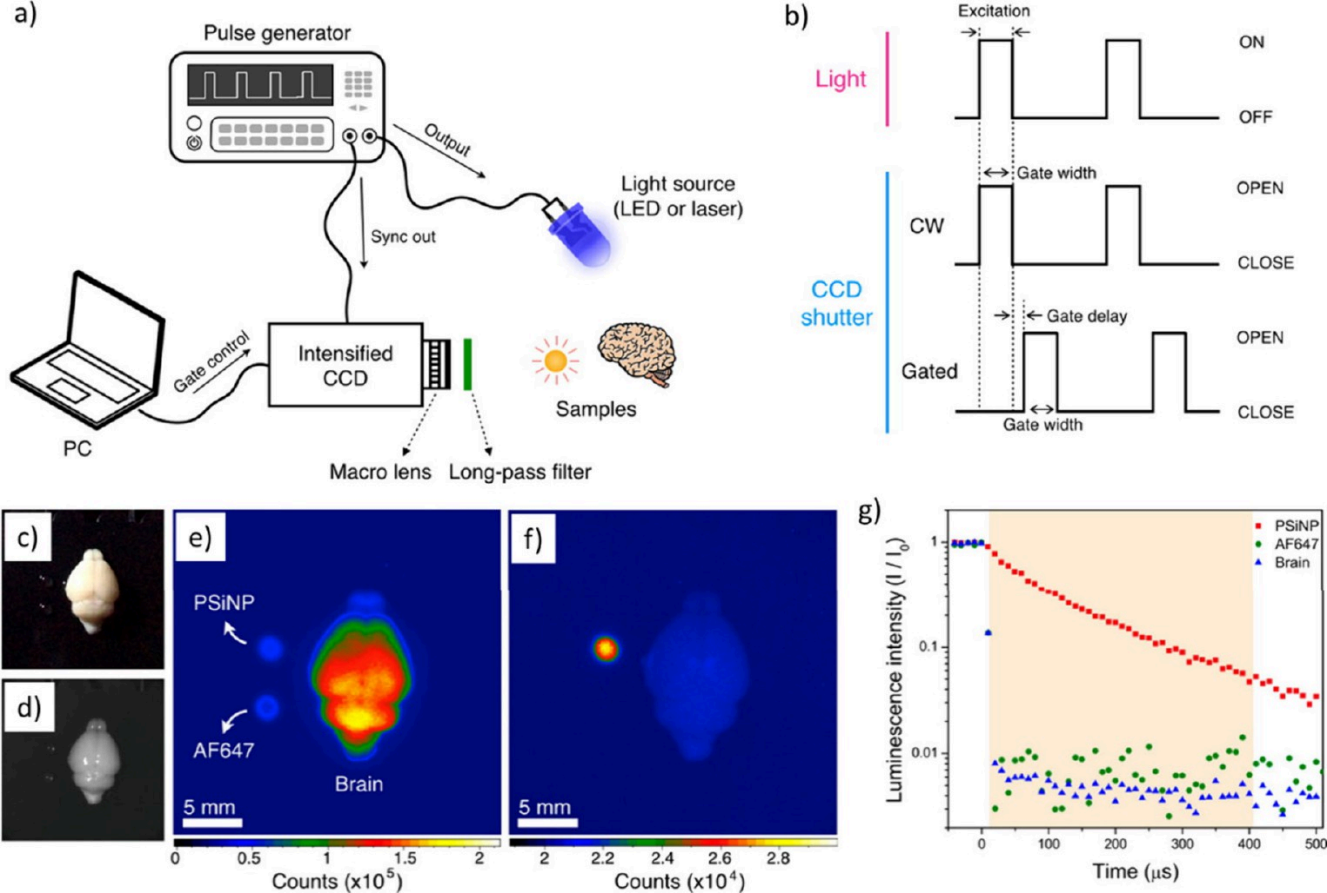
Methodology and examples of data obtained for gated luminescence imaging of Si nanoparticles (GLISiN) in mouse brain tissue compared with steady-state imaging. (a) Schematics showing the instrumental setup. The intensified charge-coupled device (iCCD) camera and the light source were controlled by an external pulse generator. In the case of laser illumination, the laser fired under control of the laser’s internal pulse generator, and the camera was configured to slave to it *via* transistor–transistor logic (TTL) digital trigger. (b) Notional waveforms for illumination and camera gating used to acquire images. The light emitting diode (LED) was triggered “ON” by the pulse generator, maintained in the “ON” position for the duration of “Gate width,” and then image acquisition terminated (“CLOSE”) at the end of the “Gate width” period. For the laser experiments, the laser fired at the beginning of “Gate width” but was only “ON” for the duration of the natural pulse width of the laser (∼8 ns). For GLISiN imaging (“Gated”), the camera was preprogrammed to energize the intensifier screen (“OPEN”) at a time delayed by “Gate delay” relative to the end of the excitation pulse. For continuous wave imaging, the camera was again programmed to be “OPEN” for the “Gate width” period, but the “Gate width” period overlapped with the laser or LED excitation pulse to generate a pseudosteady-state measurement. (c) Digital color photograph (from an iPhone 5, Apple Inc.) and (d) grayscale image (from an Andor iCCD) of mouse brain obtained under ambient light. (e) Continuous wave and (f) GLISiN images of the same brain under UV LED excitation (λ_ex_= 365 nm, λ_em_= 460 nm long-pass filter; gate width, 400 μs, 40 accumulations; gate delay for continuous wave = 0 μs, gate delay for GLISiN = 5 μs). Phantom samples corresponding to 150 ng of porous Si NPs (PSiNP) and 2.5 ng of the molecular dye Alexa Fluor 647 (“AF647”) were added next to the brain for comparison, as indicated. Note that the signals from the AF647 sample (fluorescence) and the brain tissue (autofluorescence), readily visible at steady state (e), almost disappear in the GLISiN image (f), whereas the longer-lived luminescence from PSiNP is much stronger in the GLISiN image. (g) Normalized intensity decay of the photoluminescence/fluorescence signals from the samples in (c–f) as a function of time after excitation pulse (gate width, 10 μs; gate step increase, 10 μs; accumulation, 20 times). Note the nanosecond decay times of the organic dye and tissue autofluorescence are too short to be resolved at the measurement time scale. The orange box depicts the “Gate width” window used to obtain GLISiN images in (f). Reprinted (adapted) with permission from Joo *et al*.^789^ Copyright 2015 American Chemical Society.

Luminescence in mesoporous silica has been demonstrated for *in vivo* imaging by the Sailor group.^787^ Of particular interest is that the luminescence lifetime of nanocrystalline silicon is typically on the order of microseconds, significantly longer than the nanosecond lifetimes exhibited by fluorescent molecules naturally present in cells and tissues. Time-gated imaging employing mesoporous silica, where the image is acquired at a time after termination of an excitation pulse, allows the discrimination of a silicon NP probe from relatively high background signals arising from tissue autofluorescence.^788,789^ For example, this discrimination enables tracking the fate of mesoporous silicon NPs containing tumor-targeting peptides with high efficiency and with contrast improvements of >100× (relative to steady-state imaging) in brain tissue, see Figure 33.

Photoacoustic (PA) imaging can benefit from the use of nanostructured porous silicon and silica particles to visualize the physiology and pathology of tissues with good spatial resolution and relatively deep tissue penetration.^790^ The method converts NIR laser excitation into thermal expansion, generating pressure transients that are detected with an acoustic transducer. The response of the PA contrast agent indocyanine green (ICG) can be enhanced 17× when it is sealed within a rigid NP. ICG encapsulated in particles composed of porous silica show greater PA contrast relative to equivalent quantities of free ICG. The improved response of the NP formulations is attributed to the low thermal conductivity of the porous inorganic hosts and their ability to protect the ICG payload from photolytic and/or thermal degradation. The translational potential of ICG-loaded porous silica NPs as photoacoustic probes was demonstrated *via* imaging of a whole mouse brain.^790^

Mesoporous silica carrier NPs can be specifically tailored to obtain a high nerve growth factor loading efficacy and continuous diffusion-driven release for a period of 4 weeks while preserving its biological activity. This result documents the therapeutic potential of mesoporous silicon materials for treating neurodegenerative diseases.^791,792^ Generation of new neurons by utilizing the regenerative potential of adult neuronal stem cells and neuroblasts is a therapeutic strategy to treat various neurodegenerative diseases, including neuronal loss after stroke. Biofunctionalized porous silicon NPs were able to increase the activity of neuroblasts both in cultured cells and *in vivo* in the rat brain.^793^ This strategy outlines possibilities to target drug effects to migrating neuroblasts and to facilitate differentiation, maturation, and survival of developing neurons. In this respect, we note that there are claims that porous silicon NPs are able to penetrate deep in brain tissues.^778^

### 3D Printing toward Brain-on-a-Chip Structures

Neurons can be artificially guided, moving toward BoC circuits. Compared to integrated circuits, the human brain processes a huge amount of data with significantly lower energy consumption.^794^ Although the speed of signal propagation between two individual cells is much lower compared to two circuit elements, processing complex data in a parallel fashion is one of the brain’s strengths.^795^ A key element for the brain’s data processing capacity and efficiency is the 3D nature of the cellular network. In 3D, the number of interconnections can be massively higher than in a 2D circuit. With a total number of *ca*. 86 billion neurons,^101,102^ an estimated 10^14^–10^15^ interconnects (synapses) in the human brain can only be achieved with a 3D-wiring scheme. Based on the general idea of *organs-on-a-chip*,^796,797^ the next step is to realize BoC circuits for emulating brain functionality and testing models of neurodegenerative diseases.^737^ Consequently, this leads to the development of 3D nanoprinted scaffolds as test-beds for tracing intercellular communication. Only with such model systems, which connect tens to thousands of cells, can deeper understanding of neuronal circuits be established.

**Figure 34:**
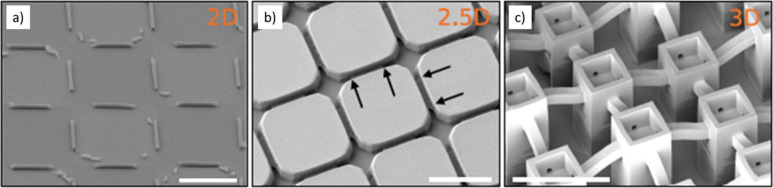
Scanning electron microscopy (SEM) images of devices utilizing spatial restriction in 2D, 2.5D, and 3D during cell culturing to control neuronal growth. (a) 2D pathways are defined by rolled-up GaAs/InGaAs microtubes. (b) Cavities and grooves with steps (arrows) are prepared by photolithography and reactive ion etching, defining 2.5D pathways for neuronal guiding. (c) Direct laser writing (DLW)-prepared scaffold structure with towerlike cavities connected by free-standing tunnels. The scale bars represent 50 μm. Original images are modified with permission from (a) Bausch *et al*.^804^ Copyright 2013 AIP Publishing; (b) Fendler *et al*.^105^ Copyright 2019 Wiley VCH Verlag; (c) Fendler *et al*.^112^ Copyright 2020 Royal Society of Chemistry.

**Figure 35:**
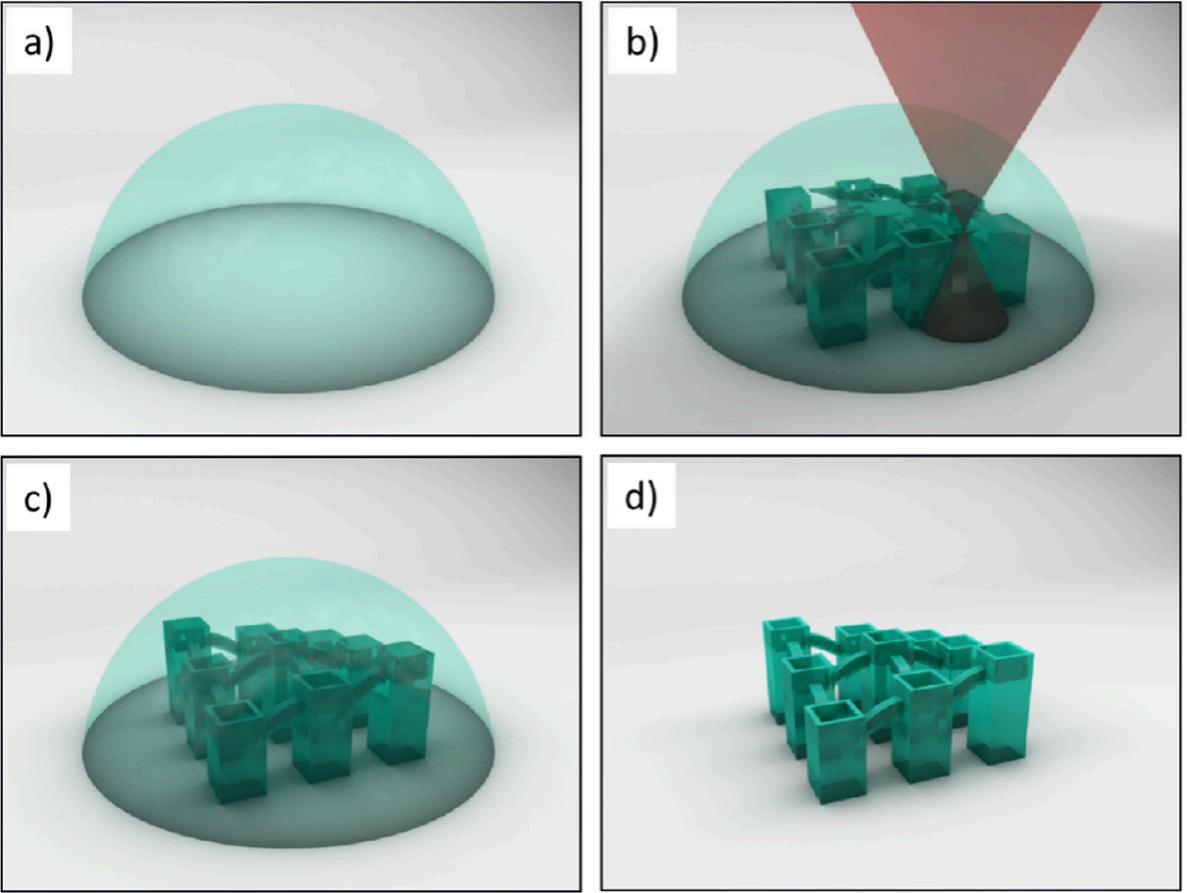
Schematic of the direct laser writing (DLW) process for fabricating a 3D scaffold structure for neuronal guidance. (a) A substrate, here a glass coverslip, with a droplet of liquid resin on top is loaded into the DLW setup. (b) Within the focal spot of a pulsed fs-laser, the resin is polymerized. The laser focus point can be moved in all dimensions through the polymeric resin leading to a 3D-defined object, (c) which is still covered with the liquid resin. (d) Developing and cleaning leads to a free-standing object on the carrier substrate. This image has been taken with permission from the Ph.D. thesis of C. Fendler, 2019.^820^

A number of 3D-printing techniques are currently revolutionizing the industry in general and medical applications in particular.^798,799^ By means of direct laser writing (DLW), which employs a multi- or (at least) two-photon-polymerization process, it is possible to fabricate structures with lateral sizes of tens of cm, heights up to several mm, with resolutions at the scale of 100 nm, see Figure 35.^800−803^ Here, the resolution is typically quoted as a volume element, *i.e.*, the voxel (ΔxΔyΔz) for which the industry standard is *ca*. 100 nm × 100 nm × 500 nm. This limit is typically determined by the pulsed laser wavelength applicable to the DLW process. This technique forms the basis for designing truly 3D scaffolds for cellular outgrowth, such as the one shown in Figure 34c. The fundamental idea in this approach is to cultivate stem cells (*e.g.*, differentiated into neurons) in a designed artificial network rather than relying on the naturally occurring outgrowth supported by astrocytes. In such structures, cell–cell interactions can be studied in 3D *in vitro.* This is a crucial step forward for the field of electrophysiology, since other platforms for measuring cell–cell interactions by microscopy or by direct transport measurements, are based on 2D support structures.

Progress in lithography and photoresists has led to steady advances in research on directed neuronal growth by contact guidance. However, most of this work consisted of building obstacles and/or channels for guided neuronal outgrowth on essentially 2D or 2.5D surfaces, highlighted in Figure 34a,b, respectively, such as tailored surface topography,^805,806^ barriers,^807^ confinement in cages, grooves or channels,^808−813^ and microtube or pillar arrays.^814−818^ In one approach, 2D layers with overgrown cells have been stacked to build quasi-3D networks.^819^ However, these stacked layers lack the flexibility of a real 3D mesh, since there are only a few *via* points connecting the different trays with 2D cellular networks. Consequently, connectivity, a crucial factor for cellular networks, cannot reach the number of connections in a real 3D cellular network such as the brain. Hence, the ability to build artificial 3D networks with a given geometry and connectivity is highly desirable. By using DLW, one is now able to design interconnects for the network and to compare them to the naturally formed web of dendrites and axons, as shown in Figure 35.

**Figure 36:**
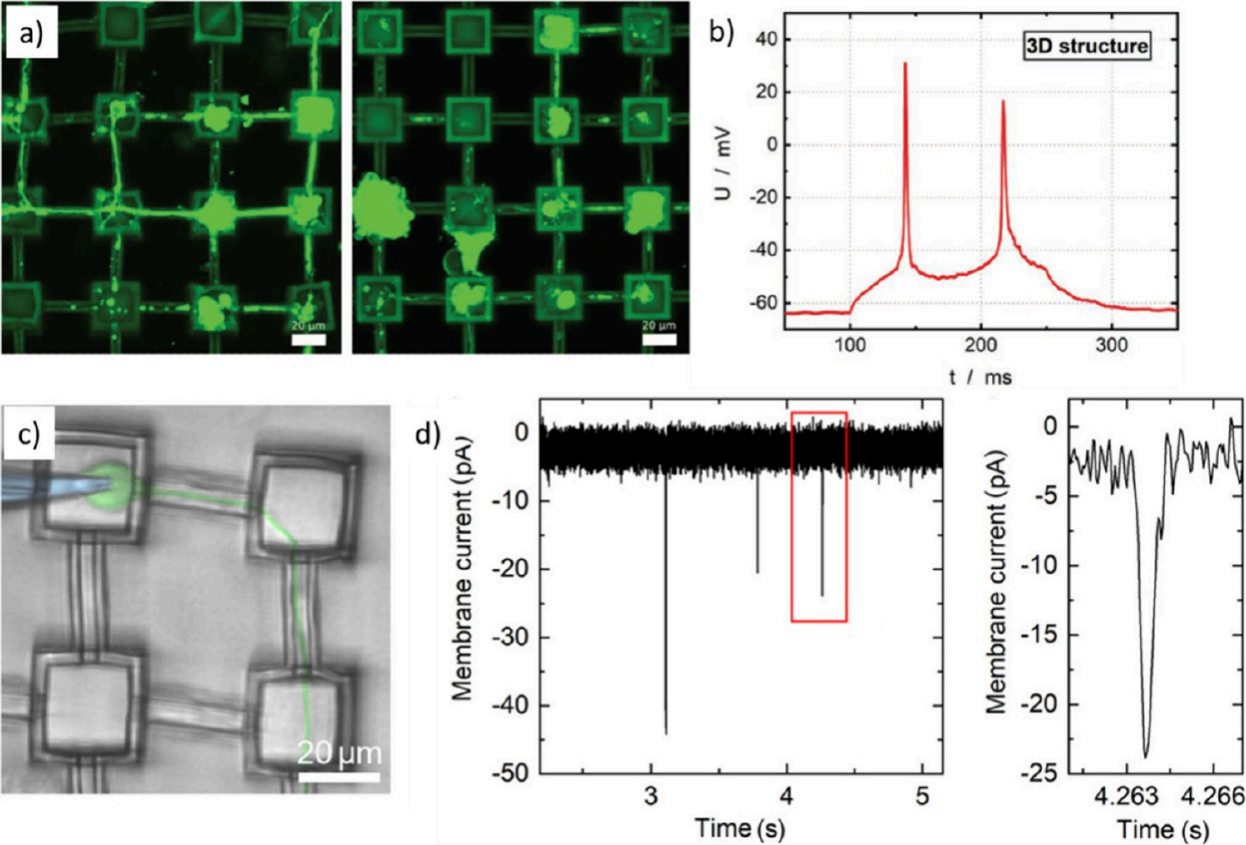
(a) Confocal microscopy images of murine cerebellar granule neurons at 10 DIV in a direct laser writing (DLW)-printed scaffold overcoated with Al_2_O_3_ and internally functionalized with poly-d-lysine. (b) Recorded action potential (AP) of a murine cerebellar granule neuron at 10 DIV inside the scaffold. (c) An example of a patch-clamping experiment on a human-induced pluripotent stem cell-derived neuron grown inside the scaffold. The pipet (blue) is approaching the cell (green) from the left. (d) Trace of excitatory postsynaptic current (EPSC) events and magnified image of a single event. The scale bars represent 20 μm. These images have been taken and modified with permission from Fendler *et al*.^105^ (Copyright 2019 Wiley VCH Verlag) and Harberts *et al*.^106^ (Copyright 2020 American Chemical Society).

Within these 3D-nanoprinted scaffolds, guided cultivation and characterization of neuronal cell networks, as highlighted in Figure 36, are now possible.^105,821^ In measurements based on conventional patch-clamping, the integrity of these artificial neuronal networks was verified by measuring APs and spontaneous excitatory postsynaptic currents, which are prerequisites for proper network signaling.^106^ The underpinning aim for realizing these circuits is to test information processing in the 3D arrays. These structures help define and then probe the size of neuronal nodes, the number of connections of a variety of nodes, and the critical distances of different nodes. In addition, the 3D scaffolds will help to resolve the influences, *e.g.*, of astrocytes, on natural 3D networks and their effects on the capabilities of such networks.^822^

## Interfacing the Human Brain: Technical Implementations

### Brain–Machine Interfaces (BMIs)—From State-of-the-Art to the Future

**Figure 37:**
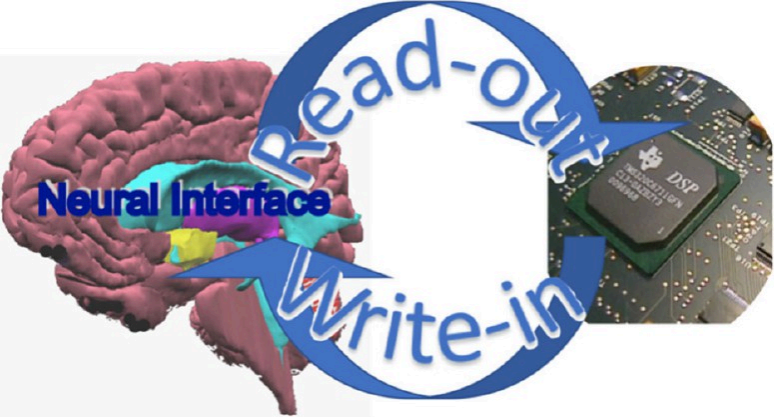
Sketch of the concept of a human–machine interface (HMI).

We have all learned to use and to control devices intuitively in our daily lives: cell phones by touch, screens by pointing, and tools by speaking to them. However, current human-machine interface technologies often need one or more layers between the mind and the device. Directly connecting the brain to engineered tools - be they computers or game or motion controllers - is a common trope in science fiction literature and movies. It appears that current progress in microfabrication technology is pushing this dream closer to becoming a reality.^823,824^ These mind-only human-machine-interfaces (HMI, see Figure 37; note that in different scientific communities, different words are used, such as HMI instead of BMI or brain–computer interface, BCI)^825^ come in at least two different flavors, depending on their invasiveness to the wearer. Given that every neuron generates a unique output signal that is distinct from all its neighbors, proximity of the recording system to the source neurons results in higher signal-to-noise ratios, better spatiotemporal resolution, and consequently information transfer rate (ITR).^826^

**Figure 38:**
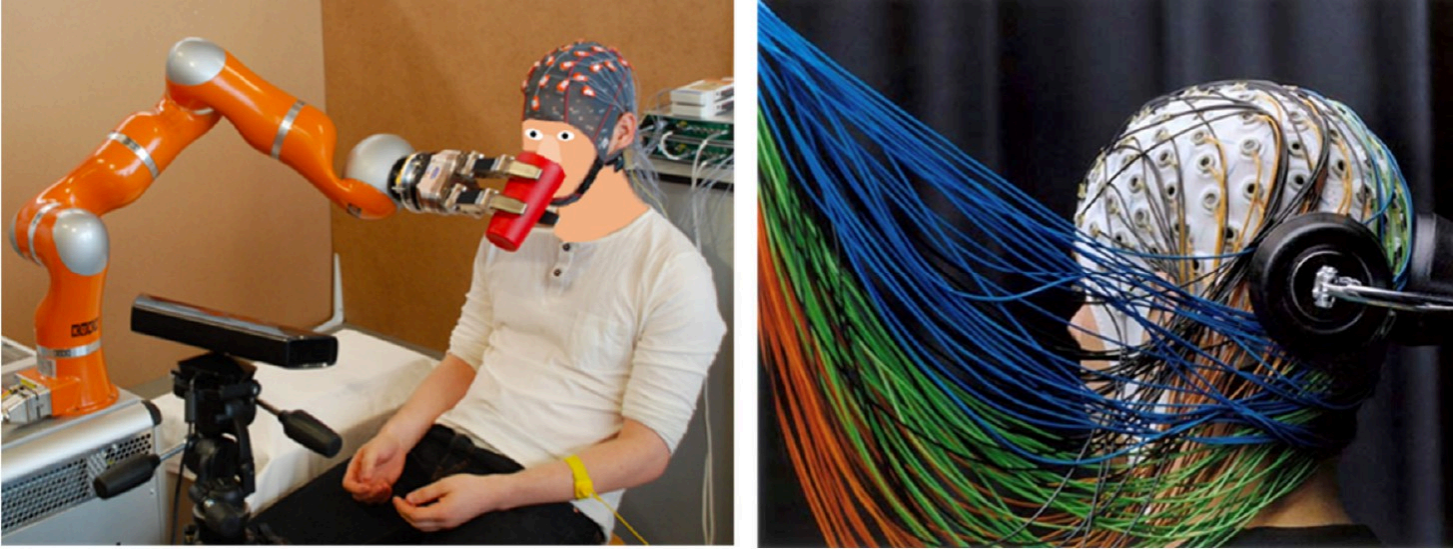
Electroencephalography (EEG) setup to control external devices showing a test person controlling a roboter arm *via* EEG communication (left) and the noninvasive high-density EEG-montage (right). Note the additional red–green–blue/corresponding depth (RGB-D) camera helping in interpreting the EEG signals. The left image is taken from Schröer *et al*.^845^ Copyright 2015 IEEE. The right image is Copyright 2024 Enker, Uniklinik Düsseldorf.

Today’s noninvasive systems mainly rely on setups based on scalp-mounted electroencephalography (EEG) measurements (see Figure 38) or complementary functional NIR spectroscopy (fNIRS) sensors, which are often called brain–computer interfaces (BCI).^827−831^ Quite some effort has been made recognizing and utilizing certain characteristic cortical potentials produced by conscious efforts of the wearer.^827,832−839^ Their activity is recorded by scalp-mounted macroscopic sensor arrays and represents the synchronized activity of millions of neurons somewhere below the electrode position or even the nourishing blood flow, only. Volumetric conduction,^840−842^ functional spectroscopy,^830^ and in rare cases, functional magnetic resonance imaging (fMRI),^843,844^ provide bird’s eye views of a large number of neurons. There is a debate about the expected maximal transfer rate, which is of fundamental importance for practical implementation. In case the transfer rate is not high enough, controlling external tools (*e.g.*, robotics) by noninvasive means would need to rely on sparse signals and thus would need to delegate true control to semiautonomous devices. Sufficient transfer rates on the other hand would allow for controlling external devices *via* noninvasive interfaces.

Higher ITR, and thus better-internalized control, can be expected by bringing the recording system into proximity of the brain’s signal sources, subdurally or intracortically. Such devices are often then called BMIs.^343,846−849^ In fact, close eavesdropping on more than a score of neurons was shown to suffice to control reward contraptions in animal experiments albeit with a low degree of freedom.^850^ More degrees of freedom may be added with increasing numbers of cortical probes, potentially leading to thousands of microwires to be implanted in superficial brain regions of subjects with paralysis. Recently, these implants provided sufficient bandwidth to support an implant, called “Brain Bridge”, enabling a patient with spinal cord injury to regain some walking ability.^7,8^ The brain displays a strong immune response to such implants and results in fibrotic encapsulation of implanted electrodes. This foreign body response may be partially reduced by using flexible implants to match the stiffness of brain tissue^851^ and by significantly reducing the form factor of the implant.^80,852^ The state-of-the-art has 1 μm-wide microelectrode sites on threads of polymer shanks that have cross sections on the order of a few microns by a few microns. Even though these flexible brain implants^336^ are bound to reach the realm of nanotechnologies, they still suffer one significant shortcoming: they need to be implanted with the help of a support structure. Wireless communication on the other hand has been demonstrated,^853^ avoiding of having the electrodes to be tethered to the outside world by wires and transcutaneous feedthroughs.

Deployed electrodes can also be used for electrical stimulation to attain bidirectional communication. When located in the correct functional, receiving brain area (cellular specificity remains hard to achieve), they can serve as conduits to enable perceptions interpreted like rudimentary touch,^854,855^ vision,^856−859^ or hearing.^860−862^ They can even mobilize paralyzed muscles *via* functional electro-stimulation,^863,864^ making the paralyzed subject walk again or control other motion.^7,8^ In order to avoid the foreign body reaction, structures are designed to minimize immune responses,^853^ which then, in turn, results in strict limits on charge injection for successful neuronal recruitment.^865−867^

We note that the language used for such interfaces is not unified in the literature and depends on the scientific background and field of the authors. Engineers sometimes call such electrical stimulation “write-in”, analogous to computer memories. But the expression “write-in” implies a lasting modification of the brain, *i.e.*, long-term synaptic changes. This is electrophysiologically different than mere electrical stimulation, which ends when the current is switched off. Realistically, it is not yet possible to “write-in” memories or specific skills into the brain. Long-lasting potentiation of specific synaptic connections has recently been achieved by optogenetic stimulation in tissue culture.^696^ However, it is unknown how complex memories are or could be coded into the brain.

There remain shortcomings in any current neuronal interface: (i) Rigid microprobes are sufficiently stiff to allow unsupported implantation,^868,869^ but are more likely to cause an immune response and to cause deterioration of the device and neuronal function.^870^ On the other hand, compliant probes are either very short,^259,262^ or need some type of support^80,871,872^ or insertion method,^171,263,873^ causing implantation trauma. Avoiding shuttles or rigid structures is desirable, but not mandatory.^851^ (ii) All currently implanted microprobe arrays run through one centralized root connecting them to the outside world by centralized signal transfer with or without processing - decentralized, *ad hoc* networking should be supported.^874^ (iii) In order to avoid a foreign body reaction, structures should stay below the micrometer size in all dimensions at any time - one future vision for this would be to use self-propelled,^875,876^ self-organized deployed, or *in situ* assembled structures,^877^ an area where nanoscience could contribute significantly. (iii) Besides macroscopic stimulation electrodes for therapeutic deep-brain stimulation in the basal ganglia, state-of-the-art recording devices do not operate in the full volume of the brain but rather stay within the superficial layers of the cortex - any point within the brain should be reliably reached by an ideal transducer.^876,878^ (iv) Even though efforts are underway to remove the transdermal wire feedthrough by inductive links (such as in cochlear implants),^861^ truly wireless operation through the skull is difficult to achieve.^879^ There are promising examples for fully untethered operation, as in ultrasound-operated so-called “Neuronal Dust” transducers^880,881^ or light-operated microscale optoelectronically transduced electrodes, spearheaded by intracellular devices in single-cell organisms *in vitro*.^882^

Given that a true BMI is supposed to function in both directions (*i.e.*, read-out and write-in), which modalities, even on a cellular scale, can be targeted and utilized in the depth of the brain?^873,883^ In the section “Colloidal Nanoparticles as Transducers...”, interactions with neurons at the level of ion channels are discussed, and how different signals can be transduced into other readouts/stimuli. Here, we revisit this topic, but now at the level of the entire brain with a macroscopic approach, as recently proposed.^884^ While the general mechanisms remain, details of the methods are different going from single-cell to whole brain interfaces. We first discuss read-out, then external stimulation, and then the possibility of a bidirectional interface.

**Figure 39:**
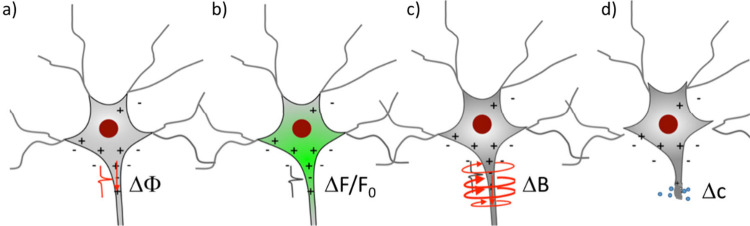
Modes of interception of neuronal activity. (a) Extracellular potential ΔΦ, (b) change in fluorescence (of genetically transfected neurons) Δ*F*/*F*_0_, (c) magnetic fields Δ*B*, and (d) concentration changes Δ*c* due to metabolic activity.

As read-outs of neuronal activity, the prime target to monitor is the electrical activity along axons. These action potentials are highly specific but difficult to discern when the distance to the recording structure exceeds 50–100 μm.^885^ As neurons process input through their cell bodies and their dendrites, there exist local field potentials (LFPs), representing synaptic background activity.^886^ These LFPs from well-ordered or layered structures, like the cortex, are the main contributors for potentials measured on the brain’s surface (electrocorticogram, ECoG) or on the scalp (EEG).^887,888^ Large numbers of neurons need to show synchronized activity in order to yield macroscopic signals, and large parts of the brain (*e.g.*, striatum, thalamus) do not have layered architectures and thus do not contribute to EEG signals. As action potentials force currents to run inside the confined spaces of axons, they cause minute magnetic fields that contribute to a measurable effect spatially complementary to EEGs. Sensitive quantum sensors are able to detect the concerted activity of huge neuronal groups in magneto-encephalographic setups (MEG).^889−891^ Optical methods are popular for monitoring the activity of many single neurons.^892,893^ Transfected or genetically modified neurons express fluorescent proteins that are highly sensitive to intracellular calcium changes and thus display their neuronal activity.^894−896^ Other sensors, such as membrane potential indicators or changes in intrinsic optical properties can be used. The development of miniature fluorescence microscopes or head-fixation for awake animals^897^ on 3D treadmills^898,899^ enables ever more sophisticated multineuron “recordings” in freely moving animals. Metabolic changes, for example, changes in oxygen consumption due to neuronal activity can be recorded, such as with fMRI, as discussed below. A summary of readouts is given in Figure 39. Note that not all mechanisms demonstrated *in vitro* are relevant for a BMI. For example, action potentials do coincide with mechanical displacements along the membrane, although some have only nanometer changes.^900−904^ This effect has only been measured in dissociated cultures with interferometric techniques and thus is not appropriate for *in vivo* interfacing.

**Figure 40:**
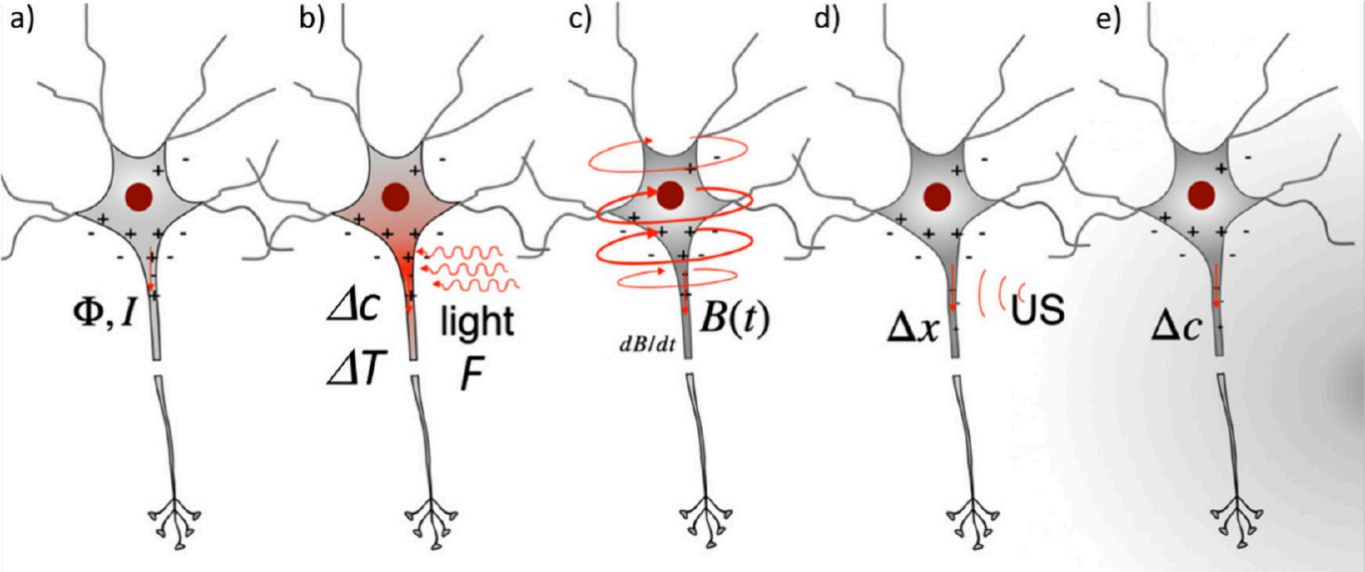
Neuronal activity can be stimulated by (a) electrical means (applied voltages Φ or currents *I*); (b) optical illumination *F*, leading to membrane depolarization, heating, or direct opening of light-gated ion channels; (c) inductive activation with oscillating magnetic fields *B*(*t*) or magnetothermal heating with NPs; (d) ultrasound activation leading to mechanical displacement Δ*x*; and (e) chemical stimulation Δ*cvia* perfused neurotransmitters.

Even when conducting extracellular stimulation of neuronal tissue by electric means, it is hard to confine the effect to few neurons or only one neuron.^855^ In extra-corporeal transcranial magnetic stimulation (TMS) fast changing and strong magnetic fields from a hand-held device induce currents inside the skull (based on Maxwells’ equations, a changing magnetic field induces an electric potential, leading to a current), which in turn influence neurons adequately oriented to the electric field. While TMS is a macroscopic and poorly targeted stimulation method, it is popular for clinical applications due its relative simplicity.^905−907^ A recent addition to the stimulation quiver originates from the observation that relatively low-energy transcranial ultrasound is able to activate neuronal tissue.^908−911^ Similarly, infrared illumination was shown to elicit compound muscle action potentials in the peripheral nerves,^912−915^ caused by either a transient change in membrane capacity ΔC^916^ or by utilizing the temperature dependence of Na channels.^917^ Infrared nerve stimulation in the CNS is still in an experimental state contrary to oscillating field-based heating of magnetic NPs.^401,439^ Upon genetic modification, light-gated ion-channels can be stimulated in whole brains, which is one of the important achievements of optogenetics (see the section “Colloidal Nanoparticles as Transducers...” for a detailed discussion). A summary of stimuli is presented in Figure 40.

One of the most promising effects in the context of bidirectional interfacing with the brain is in the linear coupling coefficient α of magneto-electric substances. In essence, due to the coupling of the electric to the magnetic fields in Landau’s description of the free energy found in a subtype of multiferroic materials, it appears possible to affect the NPs’ polarization by their surrounding magnetic field and *vice versa*.^918^ “The time-integrated current per unit area directly represents the magnetically induced change of polarization [···], that is, α = ∂*P*/∂*H*, ignoring higher-order terms”.^919^ Magneto-electric-materials are thus macroscopically used^920−922^ to satisfy both directions required for BMIs by *reading-out* external electrical potentials based on a probe’s magnetization or by *writing in* stimulating currents with variations in external magnetic fields. What is needed to satisfy both directions in one setup and *in vivo* is a localized method to detect the magnetization of deployed probes and forcing those probes to inject stimulating currents.^918,923−926^ Read-out of local field potentials enables monitoring brain states and current injection enables neuromodulation.^566,927^ Crucially, high local specificity (< ∼mm^3^) and fast temporal resolution (<20 ms) has to be achieved to interact with the brain on a functional level (in comparison: single neurons are in the range of 3000 μm^3^ and act on millisecond time scales). This specificity may be potentially achieved within the boundary conditions provided by magnetic particle imaging (MPI), a recent imaging method based on superparamagnetic NPs.^928,929^ Current preclinical MPI setups promise to provide voxel sizes—as defined by their field-free spot size—of less than a cubic millimeter. Within this field-free spot, a modern MPI is able to detect subtle changes in the magnetization of deployed superparamagnetic NPs influenced by their physicochemical environment (multicolor imaging).^930,931^ By deploying magneto-electric NPs into the brain parenchyma and utilizing fast MPI scanning, intracranial LFP recordings may become feasible. As the MPI hardware enables fast switching of local magnetic conditions (∼15 mT), manipulation and stimulation of magnetoelectric NPs become another opportunity.^925,927,932^ Without external machinery, some specificity may arise from deploying different, well-defined NPs showing discernible reactions to different activating magnetic waveforms.^933^ Extending that idea even further and adding an externally (MPI-) provided readout specificity, some type of combined barcoding of regions and environmental parameters might be conceivable. In any case, deployment of NPs might be achieved by large-scale and unspecific magnetic gradients, forcing them through the brain’s BBB^925^ or by locally heating the brain, thus opening the BBB.^934−936^ Despite some preliminary experiments in this direction, crossing the BBB, however, has not yet been established.

Even with current devices, nanomaterials are capable of improving performance. One improvement aims at the actual surface area of implanted microelectrodes by depositing nanomaterials as an intermediary between classical metal layers and the electrogenic tissue, as discussed above. This strategy might both reduce the otherwise (size-dependent) high impedance of microelectrodes and increase the charge injection capacity of such modified electrodes.^46,47,49,64,352,937−939^ Similar effects can be achieved using conductive polymers, such as PEDOT:PSS, which provide the benefit of an all-organic microelectrode assembly and are potentially better suited for close contact with the wet and soft tissue environment.^252,940−944^ The PEDOT matrix can also be utilized as a carrier for carbon nanotubes, thus further improving coupling to cells and tissue.^58,193,254,255^ The PEDOT matrix can even be used to distribute pharmacological agents actively at the point of interest.^346,943^ In the context of electrical stimulation of neurons, a two-photon photoelectric effect has been employed to produce activating potentials *via* untethered, implanted carbon fiber electrodes with tissue heating reported to staying within safe limits.^945^

### Going beyond Classical Head-Mounted EEG Recording Devices

Conventional skin-mounted EEG sensors (Figure 38) consist of rigid metal electrodes. To minimize the contact impedance and maximize the SNR, a conductive gel is applied between the electrode and the scalp. However, the conductive gel dries out in hours, and the measurement quality deteriorates rapidly. For the widespread application of HMI, including its applications to mobile electronics and human-friendly robotics, long-term reliable interfacing is critical, which requires breakthroughs in interfacing electrodes. An ideal electrode could be in the form of skin-like dry electrodes, *i.e.*, stretchable, ultrathin, and conductive. Then, the electrode can make conformal contact with the rough skin surface, which in turn leads to low contact impedance and high SNR even without conductive gel. Stretchable and ultrathin electrodes can minimize motion artifacts, even under dynamic environments. To achieve this overarching goal, stretchable conductive nanocomposites as materials for skin-mounted electrodes have been studied.^77^ For example, to maximize the softness and stretchability of the nanocomposite, hydrogels and elastomers have been used as elastic media.^77,946^ To maximize the conductivity of the percolated network between nanomaterials, metal nanomaterials with high aspect ratios such as ultralong silver nanowires have been used.^947^ For long-term biocompatibility to human skin, a nanocomposite based on core–shell (silver–gold) nanowires was developed.^948^ Recently, an extremely conductive, elastic, and ultrathin nanomembrane based on a monolayer of nanowires and poly(styrene-*b*-(ethylene-*co*-butylene)-*b*-styrene) (SEBS)^949^ was developed using a float assembly method, which features metal-like conductivity (>100,000 S/cm), high stretchability (∼1000%), ultrathin (<1 μm), and high-resolution patternability (<20 μm resolution).^950^ Such advances in materials will enable the fabrication and application of high-quality long-term HMIs.

Besides using the electrical signals from neurons to realize the connection between humans and machines, there are many interactive devices or systems capable of perceiving physical signals from humans, such as pressure, strain, motion, light, and heat. For example, to endow haptic/motion perception on BMIs, Chen *et al*. reported sensor memory devices that integrated resistive pressure/strain sensors with resistive switching memory devices.^951,952^ To implement learning at the device/system level, they further developed a neuromorphic tactile processing system using synaptic transistors. Such systems mimic perceptual learning - the process whereby sensing abilities improve through experience, which can differentiate the spatiotemporal features of touched patterns for recognition.^953^ To enable active feedback on BMI interfaces, they reported an artificial somatic reflex arc capable of self-optimizing motion feedback by introducing stimuli-triggered electrochemical actuators.^954^ Such an artificial system can also be broadened to optical stimuli by photoreceptors that produce optically mediated motion learning.^955^ Besides exploiting memristors or transistors to implement the neuromorphic intelligence on BMI interfaces, Bao *et al*. reported an artificial mechano-receptor system that can sensitively perceive pressure stimuli and transduces pressure into digital frequency signals using ring oscillators.^956^ Such frequency signals mimic the action potentials in biological system, which can be further integrated with a synaptic transistor and trigger the motion of motor nerves.^957^

As noted above, current BMIs mostly respond to physical signals or rely on electrical signals to realize the bidirectional connection.^958^ In biological systema, neurons use neurotransmitters as the chemical messengers for communication, which also play important roles in shaping high-level functions such as learning and emotion.^959^ As current BMIs cannot respond to chemical messengers, there is a communication modality mismatch issue when electrical BMIs directly connect with biological systems, especial neurons. In addition to the aptamer-FETs discussed above, which could serve this function, researchers also tried to bridge this gap by constructing BMIs capable of chemical communication *via* neurotransmitters. Santoro *et al*. reported a neurotransmitter-responsive synapse using PEDOT:PSS-based transistors where the redox potential of dopamine tunes the conductance of the PEDOT:PSS neuromorphic channel.^960^ To match the mechanical properties of tissue, Bao reported a tissue-like soft neurotransmitter sensor called NeuroString based on a stretchable interconnected graphene/NP network. This NeuroString allows chronic *in vivo* real-time, multichannel, and multiplexed monoamine sensing in the brains of freely moving mice.^961^ Besides neurotransmitter recognition, interneurons can also adaptively release neurotransmitters to trigger neighboring neurons. Adaption or plasticity of interneurons, referring to their ability to tune the connection weight dynamically, is believed to be the basis of memory formation. Hence, to communicate seamlessly with the interneurons, Chen *et al*. reported a chemically mediated artificial neuron that can both receive and release the neurotransmitter dopamine (DA) adaptively. The artificial neuron is an integrated system that detects DA *via* a DA sensor, processes the sensory signals with synaptic plasticity using a memristor device, and stimulates the DA release through a heat-responsive hydrogel. They demonstrated that the system responds to DA exocytosis from rat pheochromocytoma (PC12) cells and releases DA to activate PC12 cells, forming a chemical communication loop with the interneuron.^962^ Such chemical BMI techniques, as a complement to current electrical BMI, can better interpret brain neurotransmitter-related information, providing avenues for neuro-rehabilitation and cyborg construction, and empower techniques for brain disease diagnosis and even cognitive control. This research direction is multidisciplinary and still in its infancy, which calls for continuous and deep collaborations of researchers from electrical engineering, materials science, chemistry, biology, medical engineering, neuroscience, and computer science.

### Flexible Nanomaterial-Based Neural Interfaces

Flexible electrode arrays are useful in recording and stimulating the brain both for neuroscience and for clinical medicine. Their geometry can be adopted toward the target structure.^963^ Based on this, here has been enormous progress from sophisticated electrodes outside the body^964^ toward implantable electrodes.^965^ Viventi *et al*. developed and demonstrated a flexible, foldable, multiplexed high-density electrode array for use *in vivo* in mapping brain activity.^966^ Park *et al*. developed a graphene-based array for similar purposes.^967^

Such electrode arrays could enable neurosurgeons to discriminate accurately between tumor-infiltrated tissue based on electrophysiological signatures and recordings. The major challenges are being able to fold properly around brain tissue crevices and uneven surfaces, which are required to maximize tissue contact and therefore signal detection capabilities with minimal tissue damage. Electrodes must also be able to detect localized signals. For example, in a brain cancer surgical resection setting, tumor infiltration areas can vary significantly between adjacent zones, so the ability to detect signals with high spatial resolution is critical to achieve effective removal of tumor tissue.

**Figure 41:**
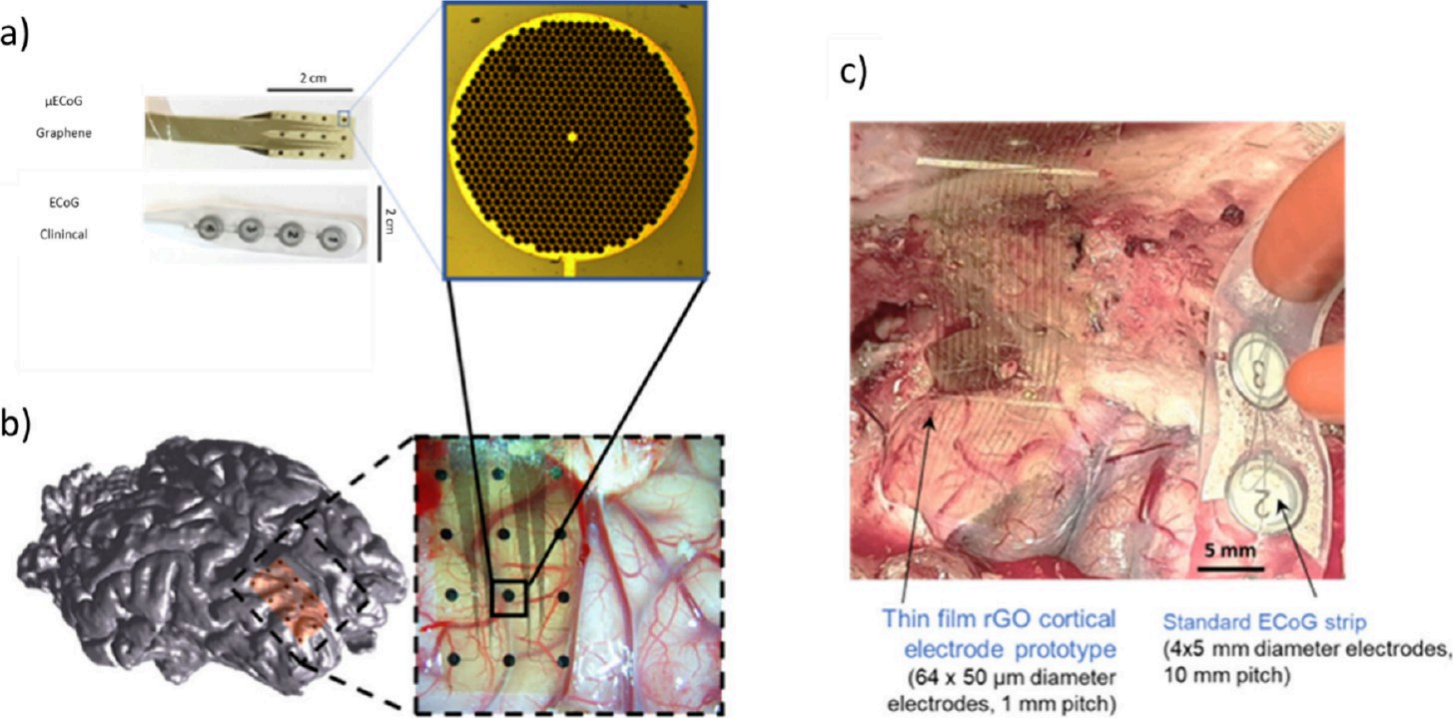
Flexible, human-scale graphene-based microelectrocorticography (μ-ECoG) device for clinical investigations. (a) Digital photograph of the graphene-based device developed by INBRAIN (top) in comparison to the clinically used ECoG metal-based strip. On the right, high magnification of one stimulating electrode contact consisting of hundreds of 25 μm graphene membranes. (b) Schematic representation of the device position on the magnetic resonance imaging (MRI)-generated image of the motor and somatosensory cortex of ovine (sheep) brain. On the right is a digital photograph of the transparent and thin-film device placed epicortically on a sheep brain. (c) Illustration of the difference in conformity between reduced graphene oxide (rGO) cortical electrodes on thin film technology (20 μm thick polyimide) compared to a silicone-based, clinically used ECoG strip. Copyright 2024 INBRAIN Neuroelectronics.

In the following, one such system is described as an example. A flexible, thin electrode array based on reduced graphene oxide electrodes has been designed, developed, and fabricated as a Class III medical device by INBRAIN Neuroelectronics (Figure 41). The device, due to its thin carrier substrate (15–20 μm thick), can adapt to surgical surfaces and cavities to ensure improved contact with tissue. The use of graphene allows the fabrication of small contacts (down to a few μm in diameter) with low impedance, so high SNR can be obtained. The small electrodes allow detection of signals from low (1–100 Hz) to high frequencies (>300 Hz), providing a significantly expanded bandwidth for monitoring neural activity. The technology uses rGO porous membranes and wafer-scale fabrication processes. These graphene-based devices are now being used in a first-in-human (FIH) clinical investigation in the acute, on-table glioblastoma resection surgical setting. This work is an example of clinical translation of graphene-based technology to be used in humans, directly interfacing with the human brain.

### Toward High-Throughput Recording Approaches

In the quest of understanding how the brain works, a commonly used approach is first to study and to understand neurons and synapses, the fundamental units of the brain. To study neurons or excitability and cell–cell communications, the commonly used technique is the so-called patch-clamp technique, which was developed by Neher and Sakmann in their Nobel Prize-winning work.^42,968^ Patch-clamp enables the direct measurement of ion channel currents and membrane voltages of individual cells and even individual ion channel proteins. The technique provides a high-quality, low-noise electrical connection to living cells in tissue by establishing high-resistance (gigaohm) seals between the pulled recording glass pipet and the cell membrane. Since the currents associated with ions that cross the membrane are in the picoampere range, this high resistance is essential for the measurements. This technique, with its diversity of manipulative (inside-out, outside-out, whole cell, single channel patch, perforated patch) and measurement methods (voltage clamp, current clamp), has become the gold standard to study cellular electrophysiology.

While the conventional patch-clamp technique is labor intensive, time-consuming, and requires a skilled operator, it is not suited for high-throughput measurements typically needed for drug discovery and drug safety assessment efforts by biotech and pharma. The development of the planar patch-clamp method^969−972^ has overcome these limitations, by employing microstructured patch-clamp chips to enable automation^973^ as well as parallelization (up to 384 well plate format) of the patch-clamp process.^41,974^ Besides these benefits in ion channel screening and drug development, chip-based approaches to patch-clamping have the potential for further miniaturization and integration of amplifiers on-chip, based on CMOS and related technologies. One can envision such fully integrated patch-clamp systems on miniaturized chips of a few square millimeters in size to be used autonomously, for example, even transmitting the recorded signals wirelessly. Such an approach could revolutionize *in vivo* applications of the patch-clamp technique. It would be a valuable and versatile tool not only for recording individual cells, but even for designing a distributed interface for the brain, enabling multifaceted connections and analyses of complex brain functions.

### Functional Magnetic Resonance Imaging (fMRI)

Among the most widely used methods adopted for profiling the functional neuroanatomy of cognitive processing is fMRI. This technology *per se* is not related to nanoscience, but we mention it, as currently it sets the benchmark. Furthermore, nanotechology in the future might contribute by developing fMRI labels.

The fMRI methodological origins of this method can be traced back to the nuclear magnetic resonance (NMR) technology.^975^ An important step forward for the study of cognitive processing was made by the discovery of the blood oxygenation level-dependent (BOLD) response.^976,977^ Oxygen represents the primary energy carrier in all metabolic cycles and is transported *via* the blood with help of hemoglobin.^977^ Considering that the brain is not good at energy storage, increases and decreases in oxygen consumption can be used to infer the involvement of specific brain regions in cognitive processes.^978^ Functional MRI indirectly measures blood flow as it detects changes in magnetic susceptibility associated with the relative concentration of both oxy- and deoxyhemoglobin.^975,979^ In this way, fMRI has enriched the way we perceive function in the brain, by enabling noninvasive assessments of neuronal activity at the full brain scale, in health and disease. Thus, fMRI has become an essential tool to assess brain function, both in animal models and in humans. Three decades after its introduction by Ogawa,^980^ the use of the BOLD signal in MRI is still the basis of a vast majority of fMRI studies, depicting the structure and activity of different neuronal networks.^981^

Investigations by means of fMRI predominantly rely on at least two test conditions (*i.e.*, experimental and control conditions). However, analyzing resting state data, for instance, does not require two conditions, and tracking continuous variations rather than categorical distinction obviously also works. There, neural activation (note that in the fMRI community “neural” rather than “neuronal” is used as preferred wording, since the signal does not come from neurons, but is more indirect, as described above) following the presentation of a stimulus (*e.g.*, a word/sentence/picture/gesture) is indirectly measured as the difference in the blood oxygenation level between experimental and control conditions.^979,982^ The activation usually shown on the contrast maps is obtained through a subtraction approach, in which the activation in the control condition is contrasted with the activation of interest (*i.e.*, experimental condition) The control condition is intrinsic to the research question being examined. It can vary from the experimental condition with respect to one single feature. Considering a typical language processing experiment, one might for instance compare differences between simple (control) and complex (experimental) syntactic sentences. On the contrary, taking the motor domain into consideration one can compare the neuronal activity, following a finger-tapping task and compare it with a rest phase. During a scanning session, images^983,984^ are acquired from many slices of the brain in all test conditions. Voxels in the brain that are involved in processing the stimulus produce a sequence of data points for which the intensity of the signal undergoes alternation synchronously with the conditions. The identification of the activation of a specific brain area during a cognitive task is given by the detection of which voxels exhibit this alternating pattern.^985^ Commonly used experimental paradigms are block and event-related designs. In the former, stimuli are presented in blocks lasting several seconds and alternating the control and the experimental condition. Since the BOLD signal has a relatively low amplitude (because of the indirectness of the acquired signal), the aggregation of events into blocks will increase the changes in hemodynamic response relative to the presented stimulus.^986^ In an event-related design, stimuli duration is usually in the range of a few seconds and stimuli are randomly presented with changing interstimulus intervals. This paradigm allows the segregation of studied cognitive processes into discrete events (*i.e.*, time points).^986−988^ Similar to the methodological approach and analyses of EEG data, this latter paradigm offers the possibility to epoch events (classify them according to category) and then average similar events in order to increase the SNR. Through the averaging approach, voxels that exhibit transient changes in fMRI signal can be isolated.^986^ The typical settings for such clinically used activity measurements of the brain offer good spatial resolution (typically around 2–3 mm; there has been, however, demonstration of <1 mm with a 7T scanner^989^), but temporal resolution remains low due to the timing of the BOLD response. Following stimulation, the peak response of the BOLD signal is usually delayed by 4–6 s and is propagated over time.^979,984,985^ Spatial resolution thus is much better than the one achieved with EEG; however, for resolving actual neuronal firing patterns, better resolution (below 1 mm) would be required. On the other hand, high spatial resolution in fMRI brings challenges related to participant motion and SNR with it, which has not yet been fully resolved.

Concerning technical feasibility, this is not yet the limit, and technically better performance is possible. Functional MRI is a burgeoning methodology in constant evolution, with important advances in the last decades that further the visualization of the brain at work, a condition that makes this technique relevant for brain–machine interfacing, as we discuss below. Improvements in the design and engineering of MRI machines have claimed the development of *in vivo* imaging techniques that measure neural signals directly instead of the BOLD signal, thereby reaching temporal resolutions down to 5 ms, while retaining high spatial resolution (we note that this study is controversial and debated).^990^ At this spatiotemporal regime, detection of sequential propagation of neural activity through functional networks full brain scale is thus possible. Improvements in fMRI readouts come not only from the technological side, but from the refinement of experimental paradigms and data processing used for fMRI studies. Major advances have been made by using machine learning techniques to decode multivariate fMRI data,^991,992^ or to infer the structure of congnitive representations.^993^ Such approaches have, for instance, been used to decode signals from submillimeter orientation columns,^994^ and overlapping representations that are coencoded in the same sets of voxels during decision making.^994^ Improvements in analysis techniques of conventional BOLD fMRI have also yielded ways to detect subsecond activation sequences.^995,996^ Recently, researchers have made impressive advances in decoding continuous language from fMRI data, using a combination of encoding and language models.^997^ In the past, user-driven statistical analysis of task-evoked BOLD-fMRI data for specifically stimulated brain networks has been the main experimental procedure; currently, most studies are based on the user-unbiased and task-free simultaneous description of multiple brain networks, achievable through resting-state (rs-fMRI) or low-frequency fluctuations (LFF-fMRI) functional MRI studies, introduced in 1995 by Biswal *et al*.^998^ This technique has bloomed in the past decade as machine learning techniques have been introduced to analyze rs-fMRI data,^999^ and sparked interest in spontaneous neuronal signals.^1000^

The fact that the BOLD effect is based on the indirect measurement of brain activity, through the recording of MRI signal changes caused by variations of blood flow and volume in response to neural activity (hemodynamic coupling), is the most well-known limitation of fMRI. Despite the intensive study of the true nature of neurovascular coupling,^1001−1003^ the role of the different cells of the neurovascular unit (particularly neurons and glial cells) in the hemodynamic response, and the recruitment of vasculature at a long distance from the local neural activity,^1004^ complicate and limit the interpretation of fMRI data. Recent advances in functional imaging have attempted to overcome this issue demonstrating that fast diffusion MRI imaging is highly sensitive for the detection of (sub)cellular neuromorphological alterations, occurring in brain tissue during activation, and corroborating previous studies that demonstrate that such neuromorphological alterations are coupled to neural activity.^1005^ Alternatives to this approach include the combination of fMRI with optogenetics,^1006,1007^ and the use of calcium-responsive contrast agents.^1008,1009^

Thanks to recent advances, functional magnetic resonance has become one of the most promising methods for noninvasive brain–computer interfaces, being currently the only method that provides full-brain function coverage at high spatial resolution. The benefits of this methodology allow linking mental activities to particular functional networks and brain regions providing a clear way to encode information for BCI users and *vice versa* (for the BCI system to decode the mental processing of the user in real time). Thus, fMRI BCIs have been used in multiple applications that include high-order cognitive tasks (thanks to the capability of fMRI to reach deep brain structures noninvasively), imagery activity, selective attention, and surreptitious language-related tasks.^1010^ Although real-time fMRI-based brain–computer interfaces have been tested to enhance performance on brain activity in healthy subjects,^1011^ the introduction of virtual environments to present high-quality stimuli to subjects in an immersive setting, presents great potential for therapeutic applications in paralyzed or impaired patients, neurological diseases and for the treatment of mental conditions that range from major depression, schizophrenia, chronic pain, addictions, and others (see Sorger and Goebels for an extended review).^1010^ The real potential of neuro-feedback-based therapies (neuromodulation and self-regulation of pathologic brain processed in a particular direction) through the use of fMRI-based BCIs is yet to be demonstrated and normalized in the clinical setting.

### Mapping Brain Neuronal Structure from the Nanoscale to the Whole Brain

There are additional direct imaging methods of the brain. While far from being used for “interfacing” the brain, such technologies contribute to understanding of the brain. The nanoscale structure and function of neurons together with their 3D distributions and connections are of key importance for understanding the brain. Related investigations of neuronal structure involve: (i) the detailed structure of the neuronal bilayer including its protein channels and receptors on the nanoscale, (ii) the next length scale involving the myelin sheath that is necessary for fast saltatory signal transduction, and (iii) the larger length scales of neuron fiber bundles and their 3D arrangements and connectivity across the whole brain on scales of tens of centimeters, *i.e.*, in total length scales over eight orders of magnitude.

**Figure 42:**
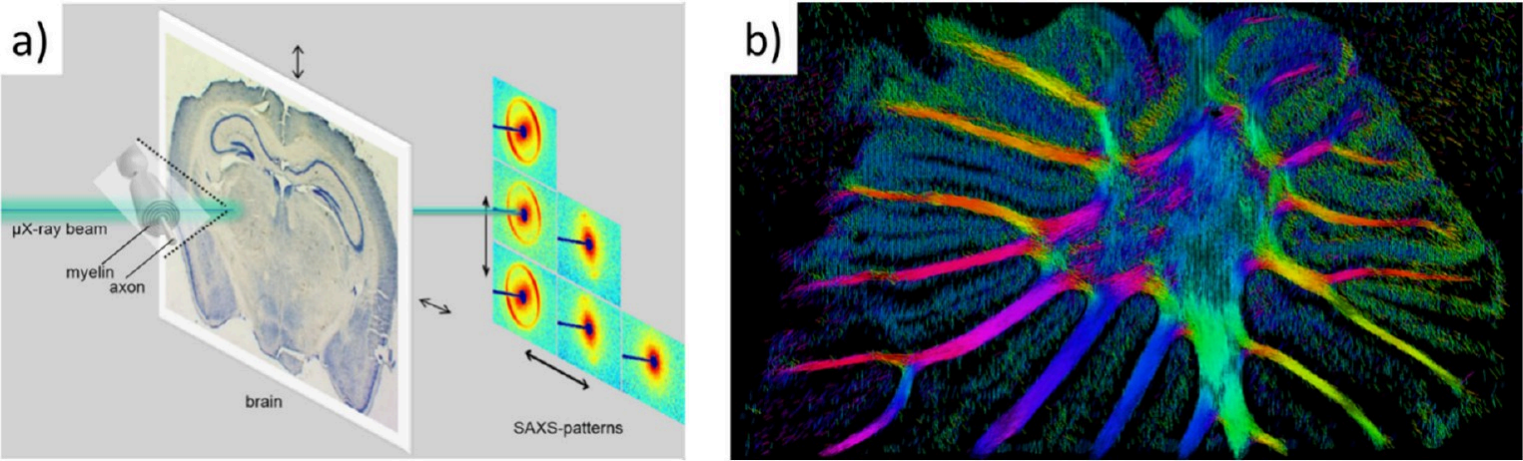
Investigation of axonal, myelin, and brain structure with small-angle X-ray scattering (SAXS) tensor tomography. (a) Experimental setup with an X-ray microbeam that is scanned across a brain section. (b) False-color-coded orientation of the measured local 3D orientation of the nerve fibers with the color representing the in-plane orientation.

The development of third- and fourth-generation synchrotrons in the past decade has provided experimental tools that now enable the study of brain structures from the nanoscale to the scale of the whole brain. These facilities provide high-intensity X-ray microbeams that can be used for high-resolution tensor tomography of the brain.^1012,1013^ In these experiments, the X-ray beam is scanned over a thin brain section while the small- and wide-angle X-ray diffraction patterns are collected as shown in Figure 42a. The multilayer structure of the myelin sheath of the axons gives rise to a set of characteristic Bragg peaks in the recorded small- and wide-angle X-ray patterns. The analysis of peak positions, intensities, and widths provides important information on the local structure and integrity of the myelin sheath. In addition, the azimuthal angle of the Bragg peaks indicates the local 3D orientation of the axon. By scanning across the brain section, the orientation of nerve fiber bundles across brain sections can be precisely mapped. Figure 42b shows the 3D fiber orientation map of an Ara brain section collected at the SAXSMAT beamline, PETRA III, Hamburg, Germany.

The method provides local structural information about the myelin sheath from the diffraction patterns length scales from 0.1–100 nm, while scanning with a resolution of 1–10 μm across the whole brain section. The maximum area that can be scanned per day is in the range of 1–4 cm^2^ depending on the spatial resolution, so whole human sections are currently out of reach. The method can be used to distinguish differences in the neuronal structure of healthy brains and brains with neurodegenerative diseases, such as multiple sclerosis, which particularly affects the structure of the myelin sheaths. The method can also be applied to neutron scattering^1014^ and has the potential to detect damage in the local neuronal structure due to neurodegenerative diseases related to ordered fiber assemblies of proteins such as tau, amyloid-β, and α-synuclein relevant for Alzheimer’s and Parkinson’s diseases.

## Ethical, Philosophical, and Legal Considerations: Do We Want to Interface Our Brain and How Far Can We Go?

While this topic is important, it has the greatest level of uncertainty. Within the framework of this article, no comprehensive answer to any of the major ethical questions that have been raised about neurotechnology will be possible. Given the high number of different forms that advanced neurotechnological systems can take, many statements about the ethics of neurotechnology must, of practical necessity, consist of considerations about any form of risky new technology. Because of these limitations, this discussion is a collection of thoughts from different perspectives about the merits of designing neurotechnological interfaces with the brain.

### How We Develop Approaches for “Neuroethics”

Discussions of the ethics governing the use of fire before its domestication by civilization would have been unhelpfully speculative, because guidelines defining the ethical use of any technology are just so many well-intentioned words unless they are paired with social institutions capable of ensuring that the guidelines are followed in practice, and the guidelines are written to as to not preclude the social benefits that the technology affords. It took centuries of cooperation between engineers, regulators, urban planners, and ordinary citizens to develop not just modern fire codes and fire suppression systems, but also tax systems capable of supporting fire brigades as public goods, analytical approaches for assessing the propagation of fire through different kinds of building materials, behavioral studies of human egress patterns, and universities and other schools capable of training enough people to have the skills and the foresight to be able to implement fire prevention best practices cooperatively. Safe use of any technology often depends upon well-designed and well-implemented social institutions.

This approach is likely to hold for any neuroengineering breakthroughs that create smooth, bidirectional interfaces between mind and machine. That is, it may take several generations of collective effort to discover how to situate the technology in the institutions of society so that the technology’s social benefit is maximized, and the relevant social risks of the technology appropriately mitigated.

The task of neuroethics is thus to develop and then to employ methods that shorten the time between breakthroughs in neuroengineering and a comprehensive understanding of how to socialize the relevant technology ethically.^1015^ Here, we discuss three contemporary approaches for undertaking this work, and offer a brief summary of some of their costs and benefits.

The first is the “Ethics by Design” (EBD) approach favored by the European Union.^1016,1017^ This approach seeks to minimize the gap between technological discovery and ethical socialization by requiring or encouraging that engineers “embed” certain general ethical and legal principles (such as fairness and respect for human agency) into the design of the technology. Proponents of this approach also frequently suggest that consultative processes be used by engineering teams to give stakeholders (usually members of populations likely end-users) control over the design process.

Another common approach, “Ethics by Implication” (EBI), seeks to forecast the potential social implications of technology, which can be used to steer engineering toward socially beneficial outcomes. Research and design efforts that have no obviously positive social implications are deprioritized, while engineering projects that appear to have immediate and obvious social benefit are prioritized. This approach is characterized by the IEEE Neuroethics Framework,^1018^ and The Royal Society’s “Brain Waves” reports.^1019^

Between these two approaches is a less discussed middle ground, “Ethics by Discovery” (EBDI).^1020^ Part of the reason that both the EBD and EBI frameworks have appeal is that they take into account a fundamental truth: no matter what its eventual form, any successful neurotechnology with real-world end-use applications will have several inherently ethical and moral properties that are neither imposed upon the technology by regulators nor simple causes of good (or bad) future social effects. The EBDI approach seeks to capitalize on the existence of these properties. As any engineering process develops, the neurotechnological systems investigated by the process, whether at the research, testing, analysis, commercialization, or any other stage, will have inherent ethical or moral characteristics that are visible even at the earliest stages of research and design, but which cannot be anticipated completely *a priori*. For example, future digital signal processors (DSPs) may be slightly safer because they run at cooler temperatures, or the latest CNTs may be preferable to other kinds of neuronal interface technology because they induce less microinflammation. Efforts to combine these technologies could then lead to a breakthrough, enabling relatively high-speed magnetothermal stimulation, which may allow clinicians to target a particular kind of depression. EBDI defines ethical neuroengineering as solving for how to stabilize and to conserve clusters of such ethical or moral effects that are “discovered” to be inherent in a particular neurotechnological design. In our hypothetical example, EBDI is practiced by discovering how to combine DSPs with the CNTs and high-speed magnetothermal stimulation, because this design increases safety and medical benefit. By practicing EBDI, as the technology further matures, its ethical and moral effects become both increasingly well-understood and increasingly easy to stabilize. Then, over longer periods of terms, the ability to conserve a cluster of mutually integrated ethical design elements can provide reasons to prefer not just certain future design and research pathways over others, but also to prioritize certain translation, socialization, and applications over others. Ultimately, when the relevant neurotechnology has matured to the point of having end-user applications, it will have a well-understood ethical profile. Namely, the cluster of features of the technology that, when integrated, yield low-cost, practical levels of end-user safety, important medical benefits, elegant failure or end-of-life behavior, and whatever other benefits can be discovered and conserved by practicing EBDI.

What are the comparative costs and benefits of the three options for determining how to socialize neuroengineering technology ethically? Some of the most obvious primary costs and benefits are summarized by Table 2.

**Table 2:** Comparison of Different Approaches for Neuroethics

	ethics by discovery	ethics by implication	ethics by design
Costs	Keeps open questions about the social impact of neurotechnology until relevant technology is relatively mature.	No known analytical techniques can be used to make accurate, precise, and generally applicable predictions about social implications of existing neurotechnology.	Not generally compatible with the difficulty and complexity of real-world engineering research and design processes.
Benefits	Easiest to integrate with engineering research and design process. Provides an objective “map” of the ethical benefits of any emerging neurotechnology, which evolves as the technology matures.	Provides direct insights into what kinds of regulations and compliance regimes may be effective for ensuring that neurotechnology is used in safe, responsible, and social well-welfare generating ways.	Easy to integrate with existing compliance and regulatory approaches. Especially compatible with the use of neurotechnology in small-scale medical applications.

Note that these three approaches are complementary, not mutually exlusive. So, it is possible to pursue all three simultaneously. Indeed, investing in how best to implement a “three fronts” approach in neuroethics may be the most efficient and effective way of defining the ethical borders for all forms and applications of neurotechnology.

### Good Scientific Practice: What Rules and Limits Should Be Considered?

The development of neurotechnology and the creation of neuronal interfaces raise significant ethical concerns.^1021^ On a broader level, the use of BCIs raises questions about privacy and autonomy, as well as data protection challenges concerning the collection and processing of brain data.^1022^ If access to neuronal interfaces is limited to affluent socioeconomic groups, there is a risk of exacerbating socio-economic inequalities.^1022^ Additionally, the long-term consequences of implantable devices on brain function and cognition are not fully understood and require further research. With respect to nanotechnology-based approaches, specific ethical considerations include the potential for unintended harm from nanoscale devices, the possibility of nanodevices causing chronic inflammation or other biological responses, and the limited ability to remove nanoscale devices once implanted. Furthermore, the development and use of nanotechnology in this field raises questions about the appropriate allocation of resources and the potential misuse of these technologies.^1016^ It is crucial for the scientific community to consider and to address these ethical issues carefully as neurotechnology and neuronal interfaces advance.

To minimize these risks, researchers working on developing nanotechnology-based neuronal interfaces can implement several best practices. First, they can engage in transparent and ongoing dialogues with relevant stakeholders, including patients, ethicists, and policymakers, to ensure that the development of these technologies is aligned with societal values and ethical considerations. Second, they can conduct thorough preclinical testing and long-term follow-up studies to assess the safety and effectiveness of these technologies. Third, they can establish clear guidelines and regulations for the development and use of these technologies to ensure that they are used in a responsible and ethical manner. Finally, they can encourage interdisciplinary collaborations and engage in ethical training programs to raise awareness and to build capacity within the field. By implementing these best practices, researchers can help to ensure that the development of neurotechnology and nanotechnology-based neuronal interfaces is aligned with ethical principles and meets the needs of society.

### Some Thoughts about the Exploitation of Machine Learning in Bidirectional Brain–Computer Interfaces

The section above, entitled “Interfacing the Human Brain”, reports on BMI, and it describes the current state of the technology as well as future advances and long-term visions for the technology. It reveals that there are many technical developments that are ethically relevant. For example, readout, external stimulation, and bidirectional interfaces have been distinguished, as well as noninvasive and *in vivo* approaches; each raises distinct ethical questions. Likewise, going beyond the limitations of classical head-mounted EEG devices is increasingly the subject of research. Also gaining momentum is research into the ethical challenges shared by computer science and neuroengineering, specifically those related to the exploitation of artificial intelligence (AI) and especially machine learning (ML) technologies. Finally, recent advances enable the construction of artificial (not just simulated, in software) neuronal networks; the potential capabilities and applications of these artificial neuronal networks (in comparison to the human brain) are discussed below.

BMI/BCI research and development has focused on medical applications. However, recently, applications have emerged, comprising entertainment/gaming as well as military use. These applications further complicate the problem of developing ethics for BMI/BCI because guidance goes beyond established principles in medical ethics.^1022^ While medical applications usually have humanitarian intentions in their design, this cannot be said about various possible future applications of nonmedical BMI/BCI technology. Possibilities include cognitive enhancement for hedonistic purposes, BMI-enhanced soldiers, performance monitoring of employees, mental surveillance for insurance purposes, memory implantation or extraction, brain jacking, merging brain and AI (see corresponding studies by Geneva Science and Diplomacy Anticipator’s, GESDA^1023,1024^). Should any of these possibilities be realized, they would create a cascade of pressing individual and societal threats and risks.

**Figure 43:**
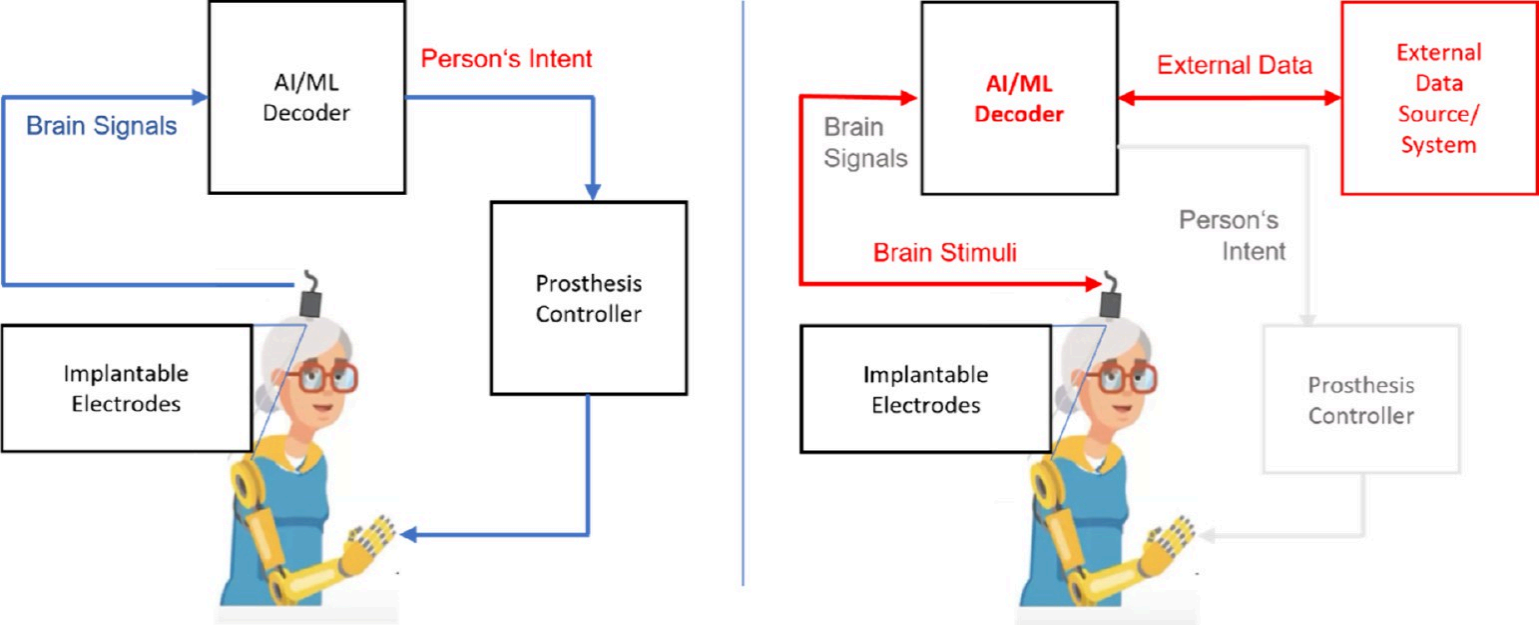
Signal flow for AI/ML-controlled neuronal prostheses.

Gallego *et al*. describe and illustrate the operation of a sample (medical, prosthesis control) BCI as follows: implantable electrodes send brain signals to an AI/ML decoder, which is supposed to learn the corresponding person’s intents by decoding the brain signals into input data for a prosthesis controller (Figure 43, left).^1025^ This sequential and unidirectional scenario can be generalized toward a bidirectional BCI (Figure 43, right) by changing the connections between the implantable electrodes and the AI/ML-decoder into a bidirectional connection allowing the AI/ML decoder not only to feed the prosthesis controller but also to stimulate the person’s brain, and additionally involve external data for this purpose. Furthermore, this architecture would also enable arbitrary interfaces between the AI/ML decoder and external systems.

Such bidirectional BCIs might have the potential to go far beyond (beneficial) medical applications; once this technology matures, it risks causing the cascading individual and social risks and threats discussed above. Such possibilities reflect the need for proper neurotechnology governance comprising good practices, standards, clinical/consumer regulations, national law, ethical guidelines, international recommendations, and international law and treaties, governance structures that should be in place well before the technology matures. Organizations and institutions currently working on that are (among others) GESDA,^1024^ the Geneva Centre for Security Policy (GCSP),^1026^ the Confederation of Laboratories for Artificial Intelligence Research in Europe (CLAIRE),^1027^ Brain Mind,^1028^ and Women’s Brain Project.^1029^

### Will the Human Brain Be Outperformed by Artificial Neuronal Networks?

As outlined above, the sheer amount of data to handle when implementing BCIs will require alternative approaches, such as artificial intelligence/machine learning, and therefore it is of interest to look into the methodology of such data processing approaches.

An interesting parallel exists between machine learning and biological systems. Deep learning describes the use of deep, i.e., possessing many internal layers of abstractions, artificial neuronal networks. As the name implies, such artificial networks were initially inspired by biological neuronal networks.

A common basis is the first mathematical model of an artificial neuron.^1030^ It describes an artificial neuron as a function with multiple inputs and a single, binary output. If the sum of excitatory inputs exceeds a threshold and the inhibitory input is not active, the neuron fires. The later perception^1031^ relaxed some of these restrictions (*e.g.*, by removing the distinction between excitatory and inhibitory) and allowed the successful learning of weights (*e.g.*, the importance of different inputs) for simple binary classification tasks from data. Another example that highlights the similarity between artificial and biological neuronal networks is especially relevant to deep learning.^1032^ Convolutional architectures^1033^ are based on the principles of locality and translation invariance of structures in an image. Over subsequent network layers, an increasingly abstract representation of the input is learned by the network. The components of this architecture correspond to known cell-types in visual neuroscience,^1034^ and the hierarchical learning of information mimics the visual cortex.^1035^ Neuronal networks have been used successfully to model other brain areas,^1036^ for example, related to memory^1037^ as well as several aspects of cognition ranging from context-dependent decision making^1038^ to insights.^1039^ Neuronal networks have also started to play important roles in the analysis of brain-imaging data,^992^ discussed below.

State-of-the-art mathematical models for neuronal networks are designed to obtain quantifiable information, *e.g.*, for predictions or classifications, from digitized input data, such as from audio or image data. Machine learning methods build models that conceptually rely on initial sets of sampled training data, from which machines are “trained” to let them “learn” intrinsic structural properties of target phenomena and processes. Machine learning algorithms provide high-performance computational methods for mathematical data analysis. The key mathematical ingredients to “teach” machine learning methods are from classical disciplines of applied mathematics, including numerical approximation, inverse problems, statistics, graph theory, and optimization. More recently, concepts from differential geometry and algebraic topology (*e.g.*, persistent homology^1040^) have been utilized to develop more powerful machine learning methods.

Despite their rapidly increasing popularity, one critical aspect of learning-based algorithms is the close dependency of their performance on the quality of their input sample (training) data. From the perspective of numerical analysis, the performance of numerical algorithms is in general evaluated by their computational complexity (efficiency), their numerical stability (robustness) and their approximation accuracy (reliability). The reliability of an approximation scheme is usually measured on the grounds of rigorous error estimates. From the underlying error analysis, the geometry of the input samples makes a direct impact on approximation errors.

To be more concrete, critical aspects concerning the input data’s properties are not only due to their huge number of data points, but also due to their very high dimension, their heterogeneous spatial distribution (*i.e.*, their varying density), and their noise content (*i.e.*, the uncertainty of their acquisition). Each of these critical aspects take a direct impact on the performance of learning-based methods of data analysis. In fact, although machine learning algorithms are classified as fast, their reliability remains to be improved. This in turn requires mathematical research along the lines of stabilization methods and data reduction schemes.

We remark that there are further limits to the correspondence between biological and artificial neuronal networks. While simple activation functions of artificial neurons mimicked the turn-on potential of biological cells, recent improvements are less biologically motivated but algorithmically superior (due to avoiding the so-called vanishing gradient problem) and computationally cheaper. Similarly, more recent developments like the widely used attention^1041^ mechanism less obviously mimic the human brain.

Machine learning is highly relevant for interfacing the human brain. Implants of electrode devices in human patients are advertised by the company Neuralink,^1042^ with neither details nor discussion of ethical issues. We note that there are many approaches from different teams in parallel, but we refer here to Neuralink as it has been most popularized in regular newspapers. Devices of this sort will produce large amounts of data, and these data will most likely be analyzed using artificial intelligence and machine learning approaches. These data should sooner or later allow us to design bidirectional BCI technology, which could lead to the situation where a computer gives control signals to a human, based on what the computer-based algorithm predicts being the intent of the human, based on signals recorded from the brain. As noted above, it is important to have legal frameworks in place, in advance of the further development of the technology.

### Some Speculative (and Provocative) Thoughts about Interfacing Neurons with Traditional Medicine

While many people would fear inserting electrodes for electrically interfacing their body, one can argue that such medical interventions are regularly performed in alternative medicines and accepted by significant numbers of people. One of the oldest interfacial “technologies” is acupuncture. This Chinese medicine was developed empirically over thousands of years. Gold or silver needles interrogating nerves or ensembles of nerves in one place of the body are claimed to affect systematic pain in the back or the heart, or to restore hearing or other functions of the body lost as a result of infection or physical damage. We note that the effects of acupuncture are disputed in several studies, but that is not the point we address here. The point is that a significant number of people, the ones who use acupuncture, do not fear such interfaces. There is not an explanation of why some special needle positions might help parts of the body, remote from the needle. Supportive data are purely empirical and have been accumulated over centuries. Our understanding of physics, however, tells us that the needle changes an electrostatic potential of the nerve locally, and this potentially could change the spiking that this nerve produces (*NB*- even this phenomenon has not demonstrated). How does the needle affect spiking? Does it depend on the place where we put the needle? Does the needle’s material play a role? All these questions could be answered if we had NPs or proteins for transducing electrical signals of the neurons into optical readout signals with high temporal-spatial resolution, which would allow us to investigate the direct effects of the needle *in situ*.

## Conclusions and Prospects

The enormous complexity of the human brain remains an obstacle to building bidirectional BCI technologies. Nanotechnology alone will not be able to solve the problems associated with building such technologies. But nanotechnology is already offering materials and platforms, which help in designing functional interface layers. As the advances and ideas described above show, these contributions will undoubtedly increase in the future. Specifically, we identify three major fields where nanotechnology can have innovative impacts. First, due to their high surface-to-volume ratios, nanomaterials are beneficial for forming intimate and close contacts between neurons and electrodes for electrical interfacing. Smaller and more biocompatible devices are envisaged, with carbon materials being of particular importance. Second, NPs are excellent transducers to convert neuronal signals into other readouts and *vice versa*. Nanomaterials generally have larger interaction cross sections with optical, magnetic, and electrical signals than organic molecules. Nanomaterials have already been highly developed as signal transducers. The practical challenges remain in decorating specific ion channels with these transducers *in vivo*, with long-term stability. Third, nanotechnology helps in the development of model systems, such as brain-on-a-chip approaches. Though this work overlaps with other technologies, such as 3D printing, the toolkit of nanotechnology will have significant impact. While not being able to emulate the brain completely, such test platforms will enable high-throughput screening approaches that would not be able to be carried out at the same scale in *in vivo* models. These technologies could already help improve some medical treatments.^1042^

## Vocabulary

patch-clamp electrophysiology: a technique used to measure the ionic currents that flow through individual ion channels, crucial for understanding neuronal function.
nanoelectrode: an electrode at the nanoscale, often employed in neurophysiology for precise measurements and stimulation of neurons.
optogenetics: a technique that utilizes light to control genetically modified neurons, allowing investigations of neuronal circuits with high precision.
conductive hydrogel-based neuronal interfaces: hydrogel materials that incorporate conductive elements to facilitate electrical communication with neurons while maintaining a hydrated environment.
brain-on-a-chip (BoC): microfluidic devices that replicate brain functions and interactions, enabling high-throughput analyses of neuronal activity.
brain–machine interface (BMI): technologies that create direct communication pathways between the brain and external devices, enabling control and interaction. Also often referred to as human–machine interface (HMI) or brain–computer interface (BCI).
neuroethics: the study of ethical, legal, and social implications of neuroscience and neurotechnology, particularly regarding brain interfacing.
